# Gene expression of peripheral blood mononuclear cells and CD8^+^ T cells from gilts after PRRSV infection

**DOI:** 10.3389/fimmu.2023.1159970

**Published:** 2023-06-20

**Authors:** Emil Lagumdzic, Clara P. S. Pernold, Reinhard Ertl, Nicola Palmieri, Maria Stadler, Spencer Sawyer, Melissa R. Stas, Heinrich Kreutzmann, Till Rümenapf, Andrea Ladinig, Armin Saalmüller

**Affiliations:** ^1^ Institute of Immunology, Department of Pathobiology, University of Veterinary Medicine, Vienna, Austria; ^2^ VetCore Facility for Research, University of Veterinary Medicine, Vienna, Austria; ^3^ University Clinic for Poultry and Fish Medicine, Department for Farm Animals and Veterinary Public Health, University of Veterinary Medicine, Vienna, Austria; ^4^ University Clinic for Swine, Department for Farm Animals and Veterinary Public Health, University of Veterinary Medicine, Vienna, Austria; ^5^ Institute of Virology, Department of Pathobiology, University of Veterinary Medicine, Vienna, Austria

**Keywords:** PRRSV, CD8^+^ T cells, PBMCs, RNA-Seq, transcriptome, swine

## Abstract

Porcine reproductive and respiratory syndrome virus (PRRSV) is a positive-stranded RNA virus, which emerged in Europe and U.S.A. in the late 1980s and has since caused huge economic losses. Infection with PRRSV causes mild to severe respiratory and reproductive clinical symptoms in pigs. Alteration of the host immune response by PRRSV is associated with the increased susceptibility to secondary viral and bacterial infections resulting in more serious and chronic disease. However, the expression profiles underlying innate and adaptive immune responses to PRRSV infection are yet to be further elucidated. In this study, we investigated gene expression profiles of PBMCs and CD8^+^ T cells after PRRSV AUT15-33 infection. We identified the highest number of differentially expressed genes in PBMCs and CD8^+^ T cells at 7 dpi and 21 dpi, respectively. The gene expression profile of PBMCs from infected animals was dominated by a strong innate immune response at 7 dpi which persisted through 14 dpi and 21 dpi and was accompanied by involvement of adaptive immunity. The gene expression pattern of CD8^+^ T cells showed a strong adaptive immune response to PRRSV, leading to the formation of highly differentiated CD8^+^ T cells starting from 14 dpi. The hallmark of the CD8^+^ T-cell response was the increased expression of effector and cytolytic genes (*PRF1*, *GZMA*, *GZMB*, *GZMK*, *KLRK1*, *KLRD1*, *FASL*, *NKG7*), with the highest levels observed at 21 dpi. Temporal clustering analysis of DEGs of PBMCs and CD8^+^ T cells from PRRSV-infected animals revealed three and four clusters, respectively, suggesting tight transcriptional regulation of both the innate and the adaptive immune response to PRRSV. The main cluster of PBMCs was related to the innate immune response to PRRSV, while the main clusters of CD8^+^ T cells represented the initial transformation and differentiation of these cells in response to the PRRSV infection. Together, we provided extensive transcriptomics data explaining gene signatures of the immune response of PBMCs and CD8^+^ T cells after PRRSV infection. Additionally, our study provides potential biomarker targets useful for vaccine and therapeutics development.

## 1 Introduction

Porcine reproductive and respiratory syndrome (PRRS) is a widespread disease responsible for leading economic losses in the swine industry worldwide (1). Only in U.S.A. estimated losses due to PRRS are approximately $664 million per year (2). One of the most remarkable features of PRRSV is the ability of the virus to suppress the host immune system which increases the susceptibility to other viral and bacterial pathogens leading to secondary infections and higher mortality rates (3–6). Recent findings showed that PRRSV causes poor innate immune response by suppressing the cytotoxic activity of NK cells and the production of cytokines. Consequently, this weakens and delays the activation of the adaptive immunity (7–9).

Despite its general success, PRRSV vaccine development has been slow and unable to prevent numerous outbreaks over the past 30 years. The root cause is attributed to the high variability of PRRSV strains, which facilitates constant evolution and sustains their virulence (10, 11). Previous research has found that modified live virus (MLV) vaccines effectively elicit humoral and cell-mediated immune responses against genetically homologous wild-type PRRSV strains, but provide only partial protection against heterologous strains (12–19). Nevertheless, compared to unvaccinated, MLV-vaccinated animals demonstrated improved immune response, with an early onset and efficient control of inflammation as well as effective cell-mediated immunity in case of infection with heterologous field strain (20, 21). However, a great source of concern is the safety of PRRSV-MLV since reports show possible reversion to virulence and recombination between MLVs and wild-type PRRSV strains (22–26). Contrary to MLV, inactivated PRRSV vaccines show better safety, but also unsatisfactory efficacy since they are unable to induce an effective cell-mediated immune response or increase production of PRRSV-specific neutralizing antibodies to reduce viral load (14, 26). The role of inactivated PRRSV vaccines in the induction of an MHC-class I restricted CD8^+^ T-cell response is also not clear and would in contrast to live attenuated vaccines postulate cross-presentation of the viral antigens. Therefore, the induction of an MHC class I restricted T-cell response, which leads to CD8 T-cell activation and effector functions such as initiation of apoptosis in virus-infected target cells, antiviral cytokine secretion and generation of vaccine induced CD8^+^ memory T cells needs to be further elucidated (27). Thus, a better understanding of the CD8^+^ T-cell response could be a key for better vaccine development and PRRSV control.

In the traditional approach, adaptive immunity and virus elimination is facilitated primarily through antigen-specific cytotoxic T lymphocytes (CTLs); however, the role of CTLs in PRRSV infection remains poorly understood. Several scientists have highlighted the necessity of new approaches in experimental analysis of CTLs in PRRSV-infected swine (28). An innovative solution to this problem is transcriptional profiling, which has the capacity to describe the underlying mechanisms of the immune response of CTLs to PRRSV infection. In the past, the focus of PRRSV research has relied on the quantification of viral load using PCR and an immunological assessment via ELISpot assay, flow cytometry, immunohistochemistry, and ELISA. Few researchers have addressed the question of transcriptional profiling following PRRSV infection. Preliminary work in this field has focused primarily on gene set enrichment of peripheral blood mononuclear cells (PBMCs) at fewer time points (29). Transcriptional profiling has been used to monitor expression changes in other swine tissues after PRRSV infection (30–32). However, the characteristics of gene expression changes in PBMCs as well as in CD8^+^ T cells upon PRRSV infection over the course of time have not been investigated in-depth.

Emerging in Lower Austria in 2015, the highly pathogenic AUT15-33 strain caused a severe clinical outbreaks (33) and has since been confirmed by studies to induce clinical signs and lung lesions (34), highlighting its virulence and potential advantageous properties. In this study we investigated the transcriptomes of PBMCs and CD8^+^ T cells in PRRSV-infected gilts at different time points after PRRSV AUT15-33 inoculation to better understand PRRS pathogenesis. Our approach combined time-series clustering analysis, protein-protein interaction (PPI) networks, extensive gene ontology (GO) enrichment, pathway analysis, and gene set enrichment analysis (GSEA) to define the innate and adaptive immunity against PRRSV more accurately. To complement our analysis, we also measured viral loads in serum and conducted flow cytometry analyses. Our aim was to identify specific gene profiles in PBMCs and CD8^+^ T cells which will provide biomarker targets useful for the development of vaccines and therapeutics. Collectively, our findings improve our understanding of gene expression profiles and kinetics of the host immune response during PRRSV infection.

## 2 Materials and methods

### 2.1 Animals and cell isolation

A total of 64 samples from eight one-year old gilts were included in the study. PBMCs and MACS-sorted CD8^+^ T cells were derived from four PRRSV-infected gilts (infected group, from an infection experiment with PRRSV strain AUT15-33, GenBank Acc. No. MT000052.1) as well as from four non-infected gilts (negative control group). At gestation day 85 (± 1), the experimental infection was induced via intranasal administration of AUT15-33 (5x10^5^ TCID_50_ per animal in approximately 5 mL into both nostrils). Blood was collected at four different time points, starting at day 0 just prior to experimental infection of the infection group, followed by blood sampling at days 7, 14 and approximately 21 (termination day, 21 ± 2) post infection. Prior to euthanasia, animals were anesthetized by intravenous injection of Ketamine (Narketan^®^ 100 mg/mL, Vetoquinol Österreich GmbH, Vienna Austria, 10 mg/kg body weight) and Azaperone (Stresnil^®^ 40 mg/mL, Elanco GmbH, Cuxhaven, Germany, 1.5 mg/kg body weight) and subsequently euthanized via intracardial injection of T61^®^ (Intervet GesmbH, Vienna, Austria, 1 mL/10 kg body weight). PBMCs were isolated from fresh heparinized blood of eight animals by density gradient centrifugation (Pancoll human, density: 1.077 g/mL, PAN-Biotech, Aidenbach, Germany; 30 min at 920 x g). Subsequently, isolated PBMCs were stored at -150°C in a freezing medium (50% (v/v) RPMI 1640 with stable glutamine (PAN-Biotech), 100 IU/mL penicillin and 0.1 mg/mL streptomycin (PAN-Biotech), 40% (v/v) fetal calf serum (FCS, Gibco™, Thermo Fisher Scientific), and 10% (v/v) DMSO (Sigma-Aldrich). All experiments were approved by institutional ethics and animal welfare committee (Vetmeduni Vienna) and the national authority according to §§26ff. of Animal Experiments Act, Tierversuchsgesetz in Austria – TVG 2012 (BMWFW-2021-0.117.108).

### 2.2 Magnetic-activated cell sorting

Enriched CD8^+^ T cells were prepared by positive selection of CD8β-labeled PBMCs using magnetic-activated cell sorting (MACS, Miltenyi Biotec, Bergisch Gladbach, Germany). For purification of CD8β^+^ T cells, thawed PBMCs (1 x 10^8^) were incubated with a primary monoclonal anti-CD8β antibody (clone PPT23, IgG1, in-house) for 20 min on ice. In a next step, cells were washed with MACS buffer (PBS w/o Ca/Mg + 2% (v/v) FCS (both Gibco™, Thermo Fisher Scientific) + 2 mM EDTA (Carl Roth GmbH, Karlsruhe, Germany), resuspended in 1.5 mL MACS buffer and incubated with magnetically labeled secondary antibody (rat-anti mouse IgG1, Miltenyi Biotec) for 30 min on ice. After a washing step, cells were resuspended in 3 mL MACS buffer and transferred onto LS columns (Miltenyi Biotec) pre-wetted with buffer. The columns were applied to a magnetic field and the negative fraction was removed by extensive washing. Afterwards columns were removed from the magnetic field and the positive fraction containing CD8β^+^ T cells was eluted in 5 mL MACS buffer. Lastly, sorted cells were resuspended in cold culture medium (RPMI 1640 + 100 IU/mL penicillin + 0.1 mg/mL streptomycin (all PAN Biotech) + 10% (v/v) FCS) and counted using a Cell Counter (XP-300 Hematology Analyzer, Sysmex Europe GmbH, Norderstedt, Germany). The purity of the positively sorted cells was above 90% (FACSCanto™II, BD Biosciences, San Jose, CA, U.S.A.).

### 2.3 RNA extraction, library preparation and sequencing

Total RNA was isolated from the samples mentioned above using RNeasy Mini Kit with on-column DNase treatment using the RNAse-Free DNase Set (both Qiagen, Hilden, Germany), following manufacturer’s protocol. Quantification and quality control of isolated RNA were assessed with Agilent 2100 Bioanalyzer (Agilent RNA 6000 Pico Kit, Agilent Technologies, Palo Alto, CA, U.S.A.). Samples with both a final yield comprised between 3.4 – 36.5 ng/µL and an average RIN value of 8.5 were prepared for sequencing with Lexogen’s Poly(A)RNA Selection Kit V1.5 and CORALL™ Total RNA-Seq Kit with UDIs (Lexogen GmbH, Vienna, Austria) to generate Illumina-compatible libraries according to the manufacturer’s guidance. Libraries were validated using the Agilent 4200 TapeStation (High Sensitivity D1000 ScreenTape Assay, Agilent Technologies) and the Qubit 3.0 fluorometer (DNA HS assay kit, ThermoFisher, Massachusetts, MA, U.S.A.). Libraries were sequenced on a S4 XP flow cell on a NovaSeq 6000 system (Illumina Inc., San Diego, CA, U.S.A.) implementing paired-end 150-bp reads. The sequencing was performed by the Next Generation Sequencing Facility at Vienna BioCenter Core Facilities (VBCF), member of the Vienna BioCenter (VBC), Austria.

### 2.4 Mapping and differential gene expression analysis

Standard raw sequencing data in BCL format were converted to FASTQ files using the bcl2fastq2 Conversion Software v2.20 (Illumina Inc.). Afterwards, the FASTQ files were imported into CLC Genomics Workbench 22.0.1 (Qiagen, Aarhus, Denmark) and the reads were subjected to adapter and quality trimming. Only reads with a Phred score of at least 25, a read length between 35 and 75 nucleotides, and no more than two ambiguous nucleotides were retained. Finally, all trimmed reads were mapped to the reference genome of *Sus scrofa* 11.1 from NCBI database (GCA_000003025.6) using default parameters of CLC Genomics RNA-Seq Analysis tool (mismatch cost = 2, insertion cost = 3, deletion cost = 3, length fraction = 0.8 and similarity fraction = 0.8). Principal component analysis (PCA) was performed using TMM adjusted mapped reads after log CPM transformation and z-score normalization. For PBMCs and CD8^+^ T cells separately, differential gene expression test for differences between all pairs of samples specified for the condition (infected vs. negative control group) using Wald test was performed. Therefore, four pairwise comparisons were made: (i) infected vs. negative control group at 0 dpi, (ii) infected vs. negative control group at 7 dpi, (iii) infected vs. negative control group at 14 dpi, and (iv) infected vs. negative control group at 21 dpi. To define differentially expressed genes (DEGs), the following set of criteria was used: fold-change > |2|, maximum of the average reads per kilobase per million mapped reads (RPKM) > 1.5 and a false discovery rate (FDR) corrected p-value < 0.01. Marker genes were identified by comparing infected to negative control group and selecting genes with non-overlapping expression ranges at time points starting from 7 dpi. The R packages ggplot2 (version 3.4.0), ggvenn (version 0.1.9) and pheatmap (version 1.0.12) were used for plotting, Venn diagrams and heatmaps visualization, respectively (R software version 4.2.2, R Core Team, GNU General Public License).

### 2.5 Time-series clustering of gene expression data

The Mfuzz R package (version 4.2) was employed for noise-robust soft clustering of gene expression data of two group samples (PBMCs and CD8^+^ T cells) along time series. For that purpose, DEGs were preselected that were differentially expressed in at least one pairwise comparison between infected and negative control groups at one time point. For the time-series clustering, the average of gene expression at each time point was used. The genes belonging to the core clusters were defined with membership value over 0.7 (α-threshold).

### 2.6 Functional enrichment analysis

Gene ontology – Biological processes (GO-BP) and Kyoto Encyclopedia of Genes and Genomes (KEGG) enrichment analysis were conducted for genes involved in time-series clustering using the ClueGO v.2.5.9 plug-in in the software environment Cytoscape 3.9.1. version (https://cytoscape.org) (35). The analysis was performed based on GO data for *Sus scrofa*. For the selection of significant GO terms and KEGG pathways, the following cut-off thresholds were used: gene count ≥ 2 genes per term, two-sided hypergeometric statistical testing corrected with the Bonferroni step-down method (p < 0.05) and a Kappa score of 0.4.

### 2.7 Genetic network analysis

Protein-protein interaction networks were generated using the Search Tool for the Retrieval of Interacting Genes (STRING Version 11.5: http://string-db.org) to find direct or indirect associations between proteins. To explore the potential protein relationships among DEGs, default settings of STRING (Network type: full STRING network; required score: medium confidence (0.400); FDR stringency: medium (5 percent)) were applied.

### 2.8 Gene set enrichment analysis

GSEA was performed to identify differentially regulated gene sets between experimental groups. Specifically, we analyzed the expression data of CD8^+^ T cells from the PRRSV-infected group at 21 dpi compared to the control group using the GSEA software version 4.3.2 (36, 37) from Broad Institute and gene sets obtained from the Molecular Signatures Database (MSigDB) (38). The analysis was performed using two “c7.immunesigdb.v2023.1.Hs.symbols.gmt” and “h.all.v2023.1. Hs.symbols.gmt” MsigDB gene sets, conducting 1000 permutations and the “gene_set” option as permutation type and a significance threshold of FDR of less than 0.05. To match the MSigDB gene set human symbols, we converted porcine gene names into their human orthologs using the HGNC Comparison of Orthology Predictions (HCOP) tool from HUGO Gene Nomenclature Committee (https://www.genenames.org/tools/hcop/) prior to analysis.

### 2.9 Quantification of PRRSV RNA

After thawing serum samples at room temperature, they were vortexed for 10 seconds and centrifuged at 16 000 x g for one minute. Hereafter 140 µL of supernatant was extracted employing the QIAamp Viral RNA Mini QIAcube Kit in a QIAcube (Qiagen, Hilden, Germany). The RT-qPCRs were performed using Luna^®^ Universal One-Step RT-qPCR Kit (New England BioLabs) on a qTower³ G Real-time PCR cycler (Analytik Jena GmbH, Jena, Germany). Primers (sense: 5´-TTTATTCTCGACTCCATCCAACC-3´, antisense: 5´-TTTATTCTCGACTCCATCCAACC-3´) and probe (FAM-5’-TCTTCTTGTGASCACGATTCGCCG-3’-BHQ1) were designed to amplify a 98 bp fragment of the PRRSV1´s conserved ORF1a region. PCR cycling conditions were 55˚C for 10 minutes, then 95˚C for 1 minute, followed by 45 cycles of 95˚C for 10 seconds and 60˚C for 30 seconds (data collection step). Moreover, 10^5^, 10^6^ and 10^7^ genomic equivalents (GE)/µL containing dilutions of a cloned AUT15-33 DNA standard were tested side by side with the samples for absolute quantification. The samples were considered positive if the RT-qPCR demonstrated more than 10^4^ copies/mL sample. Blanks consisting of sample-free extracts, which were produced simultaneously to each extraction process as well as no template controls served as negative controls. As a part of a multiplex approach beta-actin mRNA RT-qPCR described by Toussaint et al. (39) was performed for each sample extract to exclude PCR inhibiting substances.

### 2.10 Flow cytometry staining

Isolated PBMCs were transferred into a microtiter plate (Nerbe Plus, Winsen, Germany) and stained using a 4-step procedure. For each panel, primary monoclonal antibodies (mAbs) and secondary reagents used are listed in **Table 1**. Incubation steps were conducted at 4°C for 20 minutes, followed by two washes with cold PBS supplemented with 10% (v/v) porcine plasma (in-house preparation) for 4 min at 400 x g and 4°C. Surface antigens were stained with mAbs followed by incubation with secondary reagents. While blocking free binding sites of the isotype-specific secondary antibodies with whole mouse IgG molecules (2 μg per sample, ChromPure, Jackson ImmunoResearch, West Grove, PA, USA), we applied Fixable Viability Dye eFluor 506 (Thermo Fisher Scientific) diluted in PBS, and then washed with cold PBS. Cells were further fixed and permeabilized using a FoxP3 fixation/permeabilization kit (eBioscience) before performing an intracellular staining for perforin. Finally, staining for intracellular antigens was performed using directly conjugated mAbs. Following two wash steps, PBMCs were resuspended in permeabilization buffer. The cell measurements were conducted on a CytoFLEX LX (Beckman Coulter GmbH, Krefeld, Germany) and FACSAria (BD Biosciences). Cytotoxic T cells were quantified over time by evaluating the surface expression of CD3, CD8α, and perforin (CD3^+^CD8α^high^perforin^+^). On the termination day (21 dpi), CD8^+^ T-cell subsets were defined as naïve (T_n_; CD8β^+^CD27^+^perforin^-^), intermediate differentiated (T_inter_; CD8β^+^CD27^dim^perforin^+^), and terminally differentiated cells (T_term_; CD8β^+^CD27^-^perforin^high^). For the phenotyping a minimum of 300,000 and maximum of 500,000 lymphocytes was recorded for each sample. Data analysis was performed using FlowJo software version 10.8.1 (BD Biosciences).

**Table 1:** Antibodies and secondary reagents used for flow cytometry staining.

Marker	Clone	Isotype	Source	Labelling	Fluorophore
CD8 T cells					
CD3	BB23-8E6-8C8	IgG2b	BD biosciences	Direct	PerCP-Cy5.5
CD8α	11/295/33	IgG2a	In-house	Indirect^A^	BV421
Perforin	δ-G9	IgG2b	eBioscience	Direct	PE
CD8 T subsets					
CD8β	PPT23	IgG1	In-house	Indirect^A^	BV421
CD27	b30c7	IgG1	In-house	Direct	AlexaFluor647
Perforin	δ-G9	IgG2b	eBioscience	Direct	PE

^A^Streptavidin-BV421, Biolegend.

## 3 Results

### 3.1 PRRS viral load

**Figure 1:**
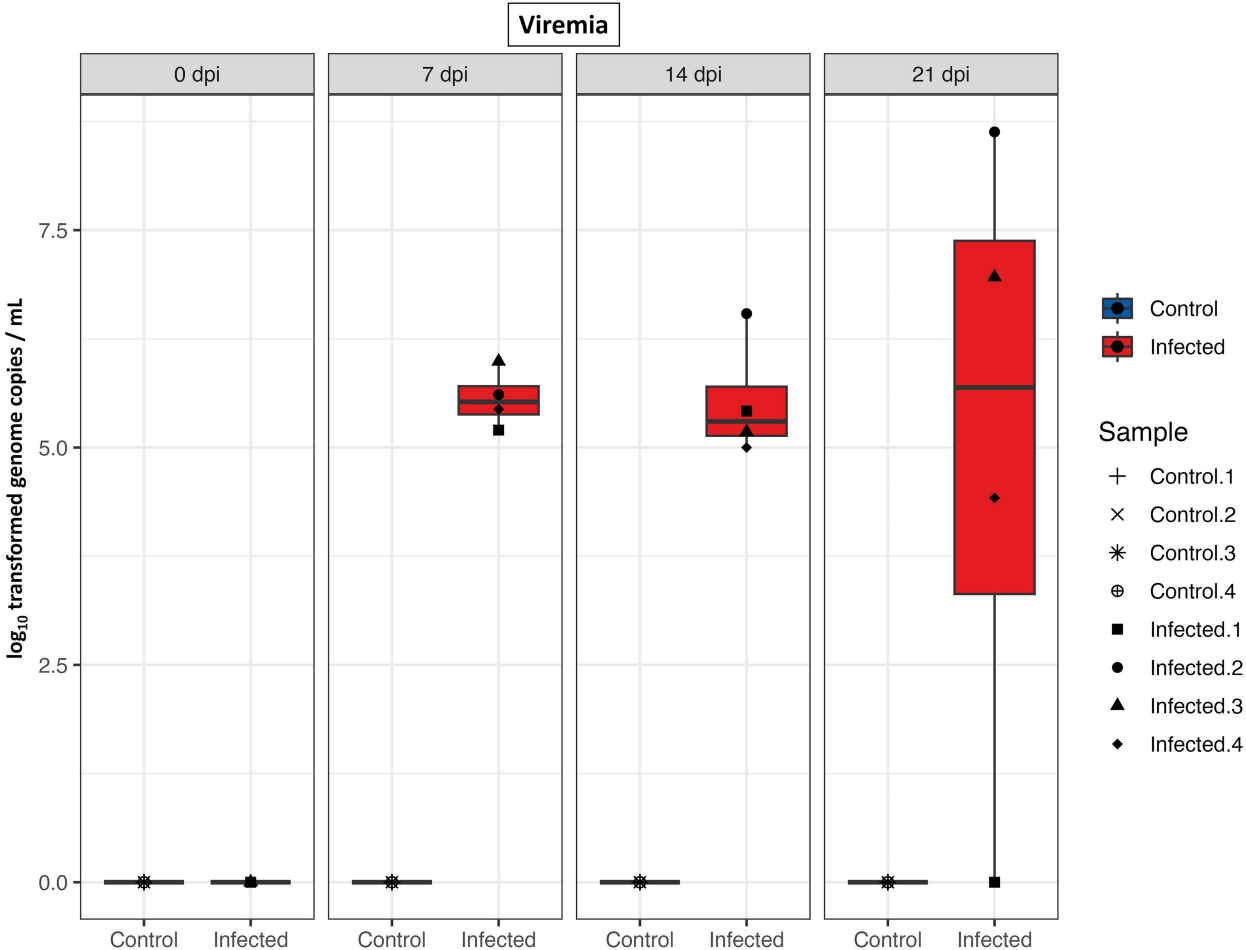
PRRS viral load in serum. Boxplots of qRT-PCR results from serum samples (log_10_ genome copies/mL) of control and infected groups at different time points after infection.

The study investigated viremia to confirm PRRS negative status of gilts prior to experimental infection and to monitor virus replication following infection. No PRRSV RNA was detected in any serum samples collected from the control group or from the infected group collected prior to inoculation on day 0, as determined by qRT-PCR analysis. On 7 dpi and 14 dpi, all infected gilts exhibited viremia. By 21 dpi, persistent viremia was observed in all but one of the infected gilts (**Figure 1**).

### 3.2 Data summary and global overview of gene expression

Sequencing 64 libraries generated over 6.02 billion paired-end reads. Each of PBMCs and CD8^+^ T cells were compared between infected and negative control group at four time points, with four replicates for each, resulting in 32 samples being sequenced for both PBMCs and CD8^+^ T cells. The percentage of mapping reads to the reference genome was between 92.08% and 94.71% (mean = 93.54%) with approximately 94 million paired-end reads per sample (**Supplementary Tables 1, 2**). Gene expression data from all samples revealed clear separation between PBMCs and CD8^+^ T cells. Given this high differentiation, we will describe each of them separately in the following sections.

### 3.3 Gene expression profile of PBMCs after PRRSV infection

**Figure 2:**
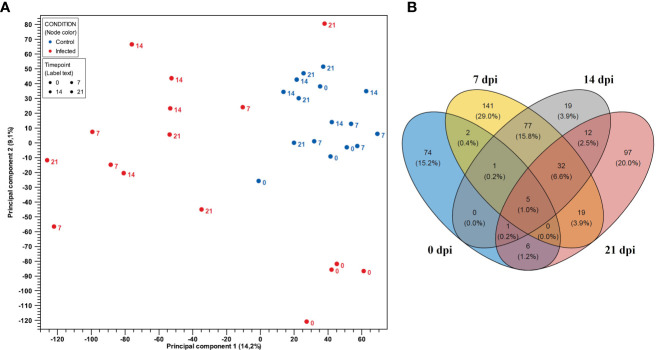
**(A)** PCA plot of expression data derived from 32 PBMCs samples of four PRRSV-infected gilts and four non-infected gilts at four time points. Red color indicates the infected samples, with blue indicating the negative control group. Numbers represent time points (0, 7, 14, and 21 dpi). PC1 explains 14.2% and PC2 explains 9.1% of the observed variance in data. **(B)** Venn diagram showing the overlap of DEGs in PBMCs between the control and PRRSV infected group from 0 dpi to 21 dpi.

Gene expressions of PBMCs from infected animals were clearly distinct from negative control groups as showed in PCA plot (**Figure 2A**). Notably, in the PCA plot (**Figure 2A**), the 0 dpi samples of the infected and negative control groups were observed to be positioned on the same side of the PC1 axis (X-axis), indicating similarities in gene expression at this time point. However, along the PC2 axis (Y-axis), there were subtle differences between samples of the infected and negative control groups at 0 dpi. Although they did not form distinct cluster together, they were the closest groups on the PC2 axis, suggesting shared underlying expression patterns or biological similarities. To define gene expression patterns of PBMCs after PRRSV infection we first looked for differentially expressed genes between infected and negative control group at the different time points (**Supplementary Table 3**). Using the Wald test for pairwise comparison we identified the highest number of DEGs (n = 277) between infected and negative control group at 7 dpi. The smallest number of DEGs was observed at 0 dpi between infected and negative control group (n = 89). Interestingly, similar numbers of DEGs were found between infected and negative control group at 14 dpi (n = 147) and 21 dpi (n = 172).

Venn diagrams were generated using DEGs to represent the overlapping and non-overlapping genes from 0 dpi to 21 dpi in PBMCs and CD8^+^ T cells, respectively. The Venn diagrams revealed that the largest overlap of DEGs was found between PBMCs from infected animals at 14 and 21 dpi (n = 77), while the intersection of PBMCs from infected animals at 7, 14, and 21 dpi exhibited a smaller overlap (n = 32) (**Figure 2B**). Additionally, a higher number of DEGs were only expressed in PBMCs from the infected group at 0 dpi (n = 74), 7 dpi (n = 141) and 21 dpi (n = 97). Notably, DEGs in PBMCs of the infected group at 0 dpi were rarely expressed at other time points. In contrast, DEGs in PBMCs at 14 dpi were mostly shared at other time points.

### 3.4 Gene expression profile of CD8^+^ T cells after PRRSV infection

**Figure 3:**
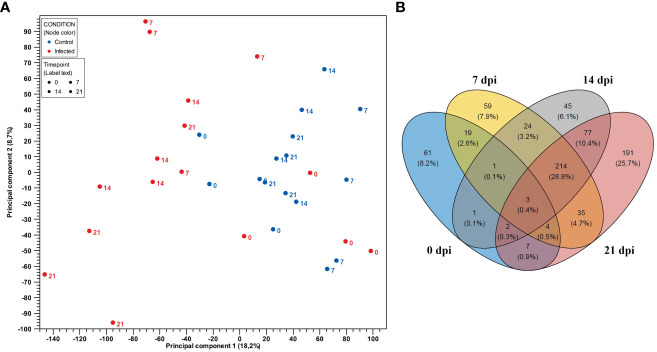
**(A)** PCA plot of expression data derived from 32 CD8^+^ T cell samples from four PRRSV-infected gilts and from four non-infected gilts. Red color indicates the infected samples, with blue indicating the negative control group. Numbers represent time points (0, 7, 14 and 21 dpi). PC1 explains 18.2% and PC2 explains 8.7% of the observed variance in data. **(B)** Venn diagram showing the overlap of DEGs in CD8^+^ T cells between the control and PRRSV-infected group from 0 dpi to 21 dpi.

PCA plot showed clear separation regarding gene expressions of CD8^+^ T cells from infected and negative control group for samples belonging to 7 dpi onwards (**Figure 3A**). We identified the highest number of DEGs (n = 533) between infected and negative control group on the last day after infection (21 dpi). In contrast, the smallest number of DEGs was observed on 0 dpi (n = 98). Interestingly, similar numbers of DEGs were discovered between infected and negative control group at 7 dpi (n = 359) and 14 dpi (n = 367). Furthermore, the Venn diagram (**Figure 3B**) revealed a high number of unique DEGs at 21 dpi (n = 191). However, we also found a high number of DEGs shared among CD8^+^ T cells from infected animals at 7 dpi, 14 dpi and 21 dpi (n = 214). In particular, the expression profile of CD8^+^ T cells of the infected group at 14 dpi shared three times more genes with the expression profile of CD8^+^ T cells from infected animals at 21 dpi (n = 77) than with the expression profile of CD8^+^ T cells from infected animals at 7 dpi (n = 24). The DEGs of CD8^+^ T cells at 0 dpi were mostly unique and intersected to some extent with DEGs of CD8^+^ T cells of the infected group at dpi 7, suggesting possible changes in the transcriptional profile of CD8^+^ T cells over time.

### 3.5 Genetic network analysis of PBMCs and CD8^+^ T cells during PRRSV infection

**Figure 4:**
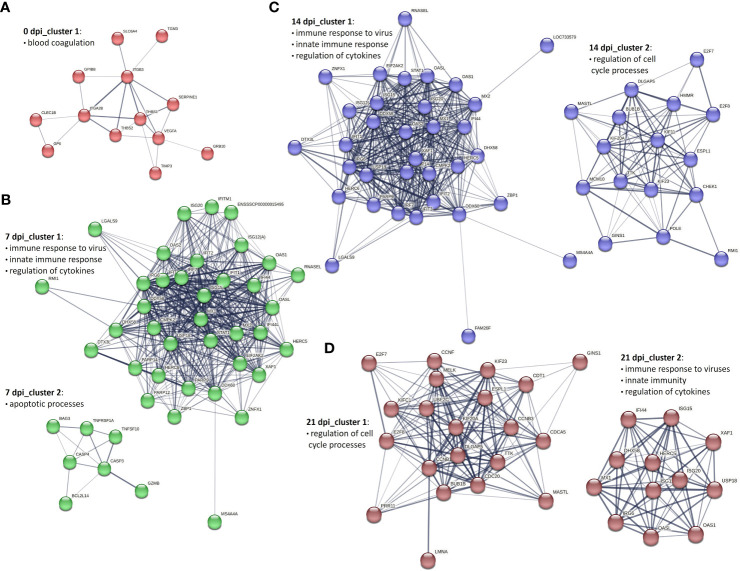
Key PPI networks of DEGs in PBMCs from PRRSV-infected animals **(A)** at 0 dpi, **(B)** clusters 1 and 2 at 7 dpi, **(C)** clusters 1 and 2 at 14 dpi, and **(D)** clusters 1 and 2 at 21 dpi.

To get an overview of the molecular processes involved in the transcriptional response of infected PBMCs and CD8^+^ T cells, we extracted information about the protein-protein interactions (PPI) of DEG genes from the STRING database and visualized them in the form of networks. After applying MCL clustering on each network, we identified one main cluster for 0 dpi. Cluster 1 contained 13 proteins associated with the blood coagulation and the smooth muscle cell migration. Two clusters were identified for 7 dpi: the first cluster included immune response to virus, innate immune response and regulation of cytokines; the second consisted of proteins enriched for apoptotic processes. Similarly, at 14 dpi, two main clusters were recorded: cluster 1 consisted of 34 proteins enriched for biological processes including immune response to virus, innate immune response and regulation of cytokines, and cluster 2 included 16 proteins linked to the regulation of cell cycle processes. A similar pattern was observed at 21 dpi, with two clusters identified but with different numbers of proteins. Specifically, cluster 1 consisted of 21 proteins associated with the regulation of cell cycle processes, and cluster 2 included 12 proteins involved in biological processes such as the immune response to viruses, innate immunity, and regulation of cytokines (**Figures 4A–D**).

**Figure 5:**
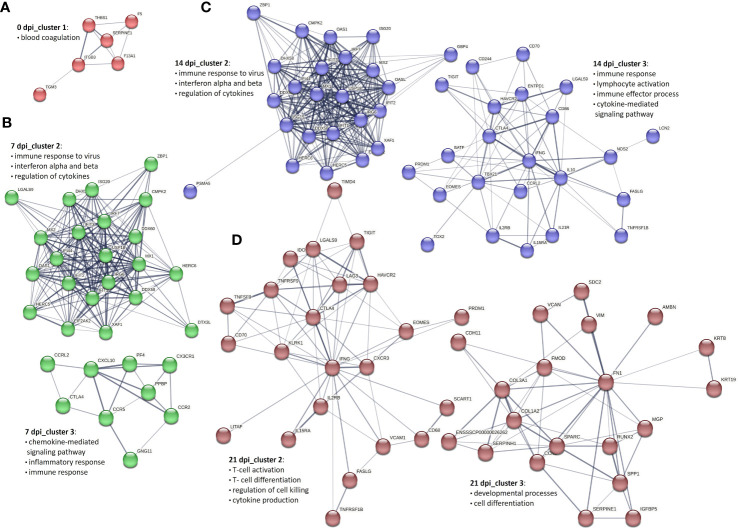
Selected PPI networks of DEGs in CD8^+^ T cells derived from PRRSV-infected animals **(A)** at 0 dpi, **(B)** clusters 2 and 3 at 7 dpi, **(C)** clusters 2 and 3 at 14 dpi, and **(D)** clusters 2 and 3 at 21 dpi.

To construct PPI networks of CD8^+^ T cells we used DEGs of CD8^+^ T cells of the infected group at the different time points of infection (**Figures 5A–D**). For DEGs of 0 dpi, similar to the PBMCs, the main cluster consisted of proteins associated with blood coagulation. PPI analysis of 359 DEGs of the infected group for 7 dpi revealed 5 clusters (**Supplementary Table 4**). Cluster 1 consisted of 149 proteins linked to regulation of cell cycle processes; cluster 2 was associated with immune response to virus, interferon alpha and beta, and cytokines; cluster 3 consisted of 9 proteins related to chemokine-mediated signaling pathway, inflammatory response, and immune response; cluster 4 and 5 involved 7 and 6 proteins correlated to cell redox homeostasis and chromatin processes, respectively. At 14 dpi, 5 clusters were identified. Similar to 7 dpi, clusters 1 and 2 included proteins related to regulation of cell cycle processes, immune response to virus, interferon alpha and beta, and cytokines; cluster 3 consisted of 23 proteins and was linked to immune response, lymphocyte activation, adaptive immune response, immune effector process, and cytokine-mediated signaling pathway. Both cluster 4 and 5 contained 9 proteins, but with different biological functions associated with it. Cluster 4 was associated with the cytoskeleton organization, whereas cluster 5 was associated with the regulation of oxidoreductase activity. Interestingly, the cluster 6 contained proteins related to the chemokine-mediated signaling pathway, chemotaxis, inflammatory response, and immune response. At 21 dpi, the largest cluster (cluster 1) consisted of 173 proteins involved in the regulation of cell cycle processes. Cluster 2 consisted of proteins associated with the T-cell activation, differentiation, adaptive immune response, regulation of cell killing, and cytokine production. Clusters 3 and 4 were linked to developmental processes and immune response, respectively. Cluster 5 was related to the chemokine-mediated signaling pathway, inflammatory response, immune response, T-cell chemotaxis, and response to cytokines. Both clusters 6 and 8 gathered proteins mostly related to the cell cycle processes. Finally, cluster 7 was specific for the immune response activation and cluster 9 for cytolysis activity (**Supplementary Table 4**).

### 3.6 Gene expression changes in PBMCs and CD8^+^ T cells during PRRSV infection

To gain a deeper understanding of the gene expression changes during PRRSV infection, we further analyzed DEGs at different time points after infection. To visualize these changes, we created heatmaps using DEGs from PBMCs and CD8^+^ T cells that were differentially expressed in at least one comparison between infected and negative control group at one time point.

**Figure 6:**
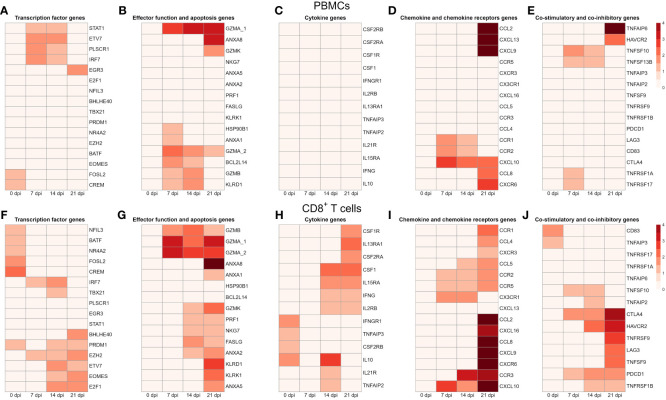
Transcription profiles of PBMCs and CD8^+^ T cells from four infected animals at four time points. All genes shown in the heat maps are differentially expressed genes at least in one material (PBMC or CD8^+^) and at least in one time point. The heatmap shows the log2 transformed expression data for these selected DEGs in PBMCs: **(A)** for transcription factor genes, **(B)** for genes associated with effector functions and apoptosis, **(C)** for cytokine genes **(D)** for chemokine and chemokine receptor genes, and **(E)** for genes of co-stimulatory and co-inhibitory molecules; in CD8^+^ T cells: **(F)** for transcription factor genes, **(G)** for genes associated with effector functions and apoptosis, **(H)** for cytokine genes **(I)** for chemokine and chemokine receptor genes, and **(J)** for genes of co-stimulatory and co-inhibitory molecules. A value of 0 indicates that there is no change in gene expression between the infected and negative control groups. As criteria to define DEGs, fold-change > |2| compared to negative control group, maximum of the average RPKM > 1.5 and a false discovery rate corrected p-value < 0.01 (FDR) were used.

For PBMCs from infected animals we found that transcription factor genes *FOSL2* and *CREM* were highly expressed at 0 dpi, while *STAT1*, *PLSCR1*, and *ETV7* were highly expressed at 14 dpi and 21 dpi (**Figures 6A–E**). It is known that *ETV7* acts as a negative regulator of the type I IFN response, in particular on antiviral interferon-stimulated genes (ISGs) (40). On the other side, the expression of *EGR3*, an early growth response 3 transcription factor that suppresses the T-cell activation and the expression of *IFNGR1* which contributes to the anti-inflammatory effects of type I INFs, was highly upregulated at 21 dpi only (41, 42).

Several genes encoding effector functions including granzymes (*GZMA*, *GZMB*, *GZMK*) and *KLRD1* were increased. Besides 0 dpi, expression of *GZMA* was increased at all other time points. Transcripts of *BCL2L14*, *GZMB* and *KLRD1* were highly upregulated at 7 dpi and 14 dpi. Notably, the apoptotic gene (*ANXA1*) and the heat-shock protein (*HSP90B1*) were markedly upregulated at 7 dpi only. In contrast, another apoptotic gene (*ANXA8*) and granzyme K (*GZMK*) were highly expressed at 21 dpi.

Looking at cytokine genes, we found that only the expression of *IRF7* was significantly upregulated at 7 dpi and 14 dpi. In case of chemokine genes, PBMCs from infected animals showed high expression of chemokine receptors (*CCR1*, *CCR2*), chemokine ligands (*CCL2*, *CCL8*, *CCL9, CXCR6*, *CXCL10*, *CXCL13*). Interestingly, *CCL2*, *CCL8*, *CXCL9*, *CXCL13* and *CXCR6* were highly upregulated at dpi 21 only. While chemokine receptors (*CCR1*, *CCR2*) were upregulated at 7 and 14 dpi, the expression of *CXCL10* was increased from 7 dpi to 21 dpi.

We identified a group of genes that encode co-stimulatory and co-inhibitory molecules, including members of the tumor necrosis factor superfamily (TNFSF) and their receptors (TNFRSF). Both *TNFSF10* (TRAIL) and *TNFSF13B* (APRIL) were highly expressed at 7 dpi and 14 dpi. Furthermore, the expression of *TNFAIP6* and *HAVCR2* (TIM3), later known as an inhibitory receptor responsible for CD8 T-cell exhaustion during chronic viral infection (43), were increased at 21 dpi only. An opposite expression pattern was observed for *TNFRSF1A* and *TNFRSF17* (BCMA) with expression levels being upregulated at 7 dpi only.

In contrast to PBMCs, we found a higher number of DEGs encoding transcription factors in CD8^+^ T cells. From transcription factor genes found in PBMCs, only *FOSL2*, *CREM*, and *ETV7* were also upregulated in CD8^+^ T cells (**Figures 6F–J**). Similarly to PBMCs, transcription levels of *FOSL2* and *CREM* were increased on 7 dpi only. On the other hand, several genes associated with the late stages of porcine CD8^+^ T-cell differentiation (44) including *TBX21*, *PRDM1* (Blimp-1) and *EOMES* were highly upregulated in CD8^+^ T cells upon PRRSV infection. Moreover, all these genes were highly expressed at 14 dpi and 21 dpi. While *BHLHE40* was significantly upregulated at 21 dpi, expression of *NR4A2*, *NFIL3*, and *BATF* was increased at 0 dpi. Previous studies showed that *BHLHE40*, a member of the basic helix-loop-helix TF family, correlates with cytokine and effector/cytolytic molecules production in human and mice (45, 46).

Looking at effector function genes, we found the upregulation of granzymes (*GZMA*, *GZMB*, *GZMK*), perforin (*PRF1*), killer cell lectin like receptors (*KLRK1*, *KLRD1*), and fas ligand (*FASLG*). Generally, the highest expression of these genes was recorded at 21 dpi. From four apoptotic genes (*ANXA1*, *ANXA2*, *ANXA5*, *ANXA8*) found, *ANXA8* showed the strongest upregulation. Interestingly, transcripts of *NKG7*, a natural killer cell granule protein 7 essential for the perforin-dependent cytolytic pathway and expressed in cytotoxic granules of activated CD8^+^ T cells (47), were highly increased at 14 dpi and 21 dpi. Besides *GZMK*, which was upregulated at 14 and 21 dpi, other granzymes (*GZMB*, *GZMA_1*, *GZMA_2*) were upregulated from 7 dpi to 21 dpi. Both *FASLG* and *PRF1* were highly upregulated at 14 dpi and 21 dpi. Moreover, genes encoding killer cell lectin like receptors (*KLRD1*, *KLRK1*) were upregulated at 21 dpi only.

Contrary to the expression profile of PBMCs, we detected a high expression of several cytokine genes in CD8^+^ T cells such as *IL10*, *IL2RB* (CD122), *IFNG* (IFN-γ), *IFNGR1* (CD119), and *IRF7*. Also, colony stimulating factors and receptors (*CSF1*, *CSF1R*, *CSF2RA*) were highly upregulated at 14 dpi and 21 dpi. Besides upregulation in expression levels of *IL10* and *IL2RB*, we also found increased expression in other interleukin receptor genes such as *IL21R*, *IL15RA*, and *IL13RA1*. Also, both genes of TNF-induced proteins, *TNFAIP2* and *TNFAIP3* were upregulated in CD8^+^ T cells.

In comparison to chemokine genes in PBMCs, we found a higher number and markedly higher expressions of chemokine genes in CD8^+^ T cells derived from PRRSV-infected animals. Moreover, the highest transcript levels of chemokine receptors (*CCR1*, *CCR2*, *CCR3*, *CCR2, CCR5*) and chemokines (*CCL2*, *CCL4*, *CCL8, CXCR3*, *CXCR6*, *CXCL9, CXCL10, CXCL16*) were detected at 21 dpi. The *CX3CR1*, a receptor of fractalkine expressed on virus-specific CD8^+^ T effector cells (48), was upregulated at 7 and 14 dpi. Also, two more chemokine genes, namely *CCR2* and *CCR5*, displayed the same expression pattern. Both *CCR3* and *CCL5* were significantly elevated in CD8^+^ T cells from PRRSV-infected swine at 14 and 21 dpi. We found a set of genes including *CCR1*, *CCL2*, *CCL4*, *CCL8*, *CXCR3*, *CXCR6*, *CXCL9*, and *CXCL16* highly expressed at 21 dpi only.

In case of co-stimulatory and co-inhibitory molecules, we observed induced expression of *PDCD1* (PD-1) and *CTLA4* from 7 dpi to 21 dpi. Both *HAVCR2* and *TNFRSF1B* were upregulated at 14 dpi and 21 dpi. Furthermore, expressions of *LAG3*, *TNFSF9*, and its receptor *TNFRSF9* (4-1BB) were significantly increased in PRRSV-infected CD8^+^ T cells at 21 dpi. Expression level of *TNFSF10* (TRAIL) was increased at 7 dpi and 14 dpi, while *CD83* was increased at 0 dpi only.

### 3.7 Time-series clustering of gene expression data in PBMCs and CD8^+^ T cells during PRRSV infection

**Figure 7:**
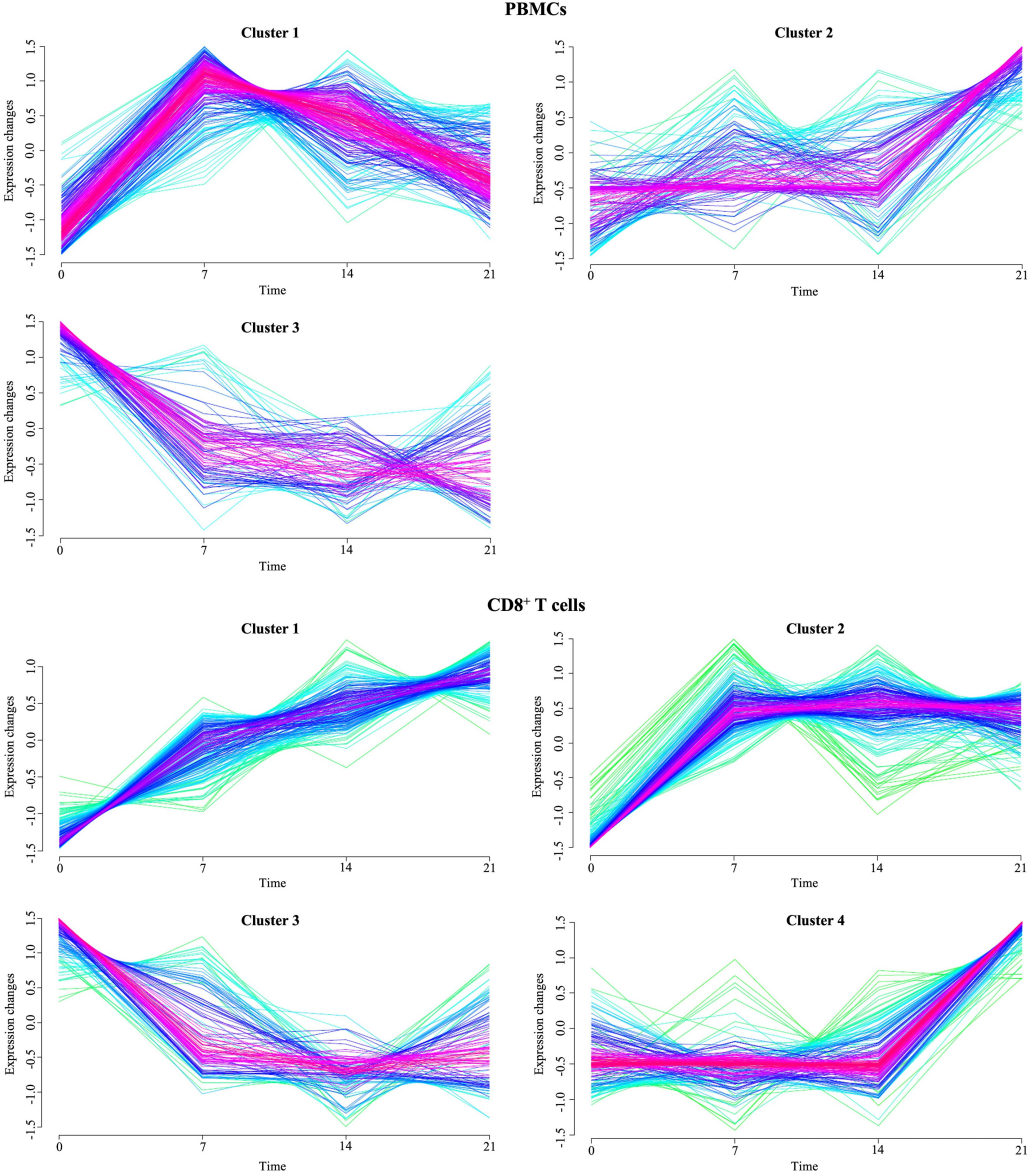
Temporal clustering of genes expressed in PBMCs and CD8^+^ T cells from PRRSV-infected animals. The Y-axis marks the expression changes and X-axis the time points (0, 7, 14 and 21 dpi). Colors indicate membership value, red and purple indicate strong membership (core of a cluster), while green and blue indicate weak membership.

To detect genes with correlated gene expression dynamics during PRRSV infection we performed clustering analysis for the union of 486 DEGs of PBMCs, which were differentially expressed in at least one pairwise comparison between infected and negative control group at one time point. For the time-series clustering, the average of the gene expression at each time point was used. We obtained three gene sets with different expression trends (**Figure 7**). Genes in cluster 1 (139) had an acute peak at 7 dpi and were then decreasing from 7 dpi to 21 dpi. An upward expression trend for 80 genes in cluster 2 was observed, while a downward expression trend was observed for 56 genes in cluster 3. Furthermore, in cluster 2, expressions of the genes were stable from 0 dpi to 14 dpi, while they increased from 14 to 21 dpi. In contrast to cluster 2, gene expressions in cluster 3 decreased from 0 dpi to 7 dpi and then tended to be stable from 7 dpi to 21 dpi (**Supplementary Table 5**).

**Figure 8:**
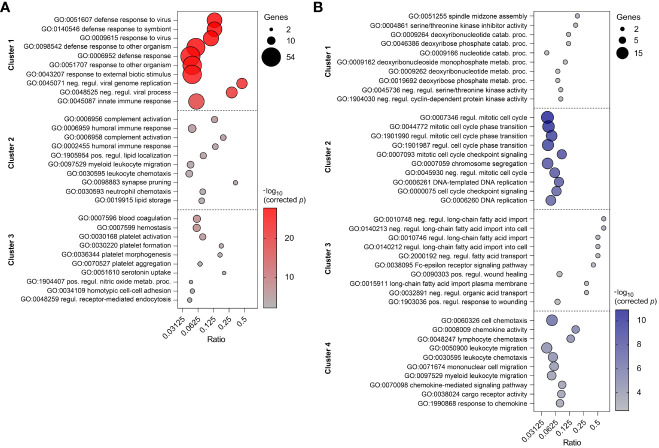
Top ten enriched GO terms in three clusters of PBMC **(A)** and four clusters of CD8^+^ T cells **(B)**. GO terms are listed in descending order of corrected p-value. The size of the bubbles indicates the number of genes enriching the corresponding annotation. Ratio refers to the number of genes found in the dataset relative to the total number of genes associated with the respective GO term.

**Figure 9:**
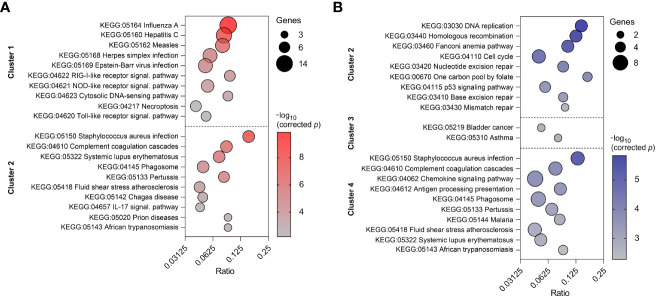
Top ten enriched KEGG pathways in two clusters of PBMC **(A)** and three clusters of CD8^+^ T cells **(B)**. KEGG pathways are listed in descending order of corrected p-value. The size of the bubbles indicates the number of genes enriching the corresponding annotation. Ratio refers to the number of genes found in the dataset relative to the total number of genes associated with the respective KEGG pathway.

To reveal the biological processes involved in each gene expression cluster, Gene Ontology and KEGG enrichment analyses were performed using ClueGO as described above. The top ten GO terms in each cluster are represented in **Figure 8A**. Cluster 1 was mainly enriched in processes involved in defense response to virus and innate immune response. Cluster 2 was associated with humoral immune response, complement activation and leukocyte chemotaxis. On the other hand, cluster 3 was enriched in genes involved in blood coagulation and regulation of receptor-mediated endocytosis. KEGG analysis revealed 10 and 12 significantly enriched pathways in cluster 1 and 2, respectively (**Figure 9A**). Cluster 1 involved genes enriched for influenza A, NOD-like, RIG-I-like, and toll-like receptor signaling pathway. The RIG-I-like receptor signaling pathway, responsible for detecting viral pathogens and generating innate immune responses, contained *CXCL10*, *DHX58*, *IFIH1*, *IRF7*, *ISG15*, and *RIGI* genes. At the same time, the toll-like receptor signaling pathway included *CXCL10*, *IRF7*, *STAT1*, *TLR7* and *TLR8* genes. The genes *C1QA*, *C1QB*, *C1QC*, *C1S*, *C3*, and *C4A* in cluster 2 were predominantly enriched in top ten pathways including *Staphylococcus aureus* infection, pertussis and complement and coagulation cascades. Genes from cluster 3 were not significantly enriched in any KEGG pathway.

The union of 743 DEGs of CD8^+^ T cells between infected and control animals at each time point were subjected to the time-clustering analysis. With the abovementioned cutoff criteria, four clusters were recorded (**Figure 7**). Among these, an upward expression trend in cluster 1 (24 genes), cluster 2 (55 genes), and cluster 4 (97 genes) was observed. Conversely, genes involved in cluster 3 (44 genes) showed a downward expression trend from 0 dpi to 7 dpi, with their expression levels stabilizing from 7 dpi to 21 dpi. Both cluster 1 and 4 reached the peak of the gene expression levels at 21 dpi. Genes in cluster 1 were characterized by continuous increase of expression levels from 0 dpi to 21 dpi. In contrast to cluster 1, gene expressions in cluster 4 were stable from 0 dpi to 14 dpi while increasing from 14 dpi to 21 dpi. Finally, cluster 2 grouped genes that primarily increased at 7 dpi and then remained at their plateau expression level from 7 dpi to 21 dpi.

Top ten GO terms for the genes involved in four clusters of CD8^+^ T cells are listed in **Figure 8B**. Genes in cluster 1 were enriched in cell cycle process (*AURKB*, *INCENP*), carbohydrate derivative catabolic process (*DUT*, *PNP*), and cyclin-dependent protein serine/threonine kinase inhibitor activity (*CASP3*, *CDKN2C*). Cluster 2 had enriched GO terms in the regulation of mitotic cell cycle, cell cycle checkpoint signaling, and DNA replication. This suggests apparent involvement in cell proliferation. Cluster 3 was enriched in regulation of long-chain fatty acid transport and Fc-epsilon receptor signaling pathway. The genes *CCL2*, *CCL4*, *CCL8*, *CXCL10*, *CXCL16*, *CXCL9*, *CXCR6*, *KLRK1*, *LGMN*, and *RARRES2* from cluster 4 were mostly enriched in top ten GO terms including cell chemotaxis, chemokine activity, and chemokine-mediated signaling pathway. In comparison to the GO enrichment analysis, the KEGG analysis revealed a smaller number of enriched pathways for the four clusters (**Figure 9B**). Genes involved in cluster 1 were not significantly enriched in any KEGG pathway. Cluster 2 contained *POLE*, *POLE2*, *RPA3*, *BLM*, *BRCA1*, *MCM6*, and *RFC4* which were enriched in DNA replication and repair, as well as the p53 signaling pathway. Genes in cluster 3 were enriched in asthma and bladder cancer pathways only. Similarly to cluster 3 of PBMCs, cluster 4 of CD8^+^ T cells was enriched in *Staphylococcus aureus* infection, pertussis, and complement and coagulation cascades. Additionally, it was also enriched in the chemokine signaling and antigen processing and presentation pathways (**Supplementary Table 5**).

### 3.8 GSEA of gene expression profile in CD8^+^ T cells from PRRSV-infected group at 21 dpi

The expression profile of CD8^+^ T cells from the PRRSV-infected group at 21 dpi showed high levels of effector-associated markers such as *TBX21* (T-bet), *GZMA*, *GZMB*, *GZMK*, *PRF1*, *KLRK1*, *KLRD1*, and *FASLG*. However, these cells also expressed several coinhibitory receptors, including *PDCD1*, *CTLA4*, *LAG3*, and *HAVCR2* (TIM3), as well as the transcription factor *EOMES*. The prolonged expression of these markers is a distinctive feature of exhausted CD8^+^ T cells (49, 50). To investigate further, GSEA was performed to determine whether expression data of CD8^+^ T cells from PRRSV-infected group at 21 dpi exhibits statistically significant differences with an *a priori* defined set of genes.

Our findings indicate that CD8^+^ T cells from PRRSV-infected group at 21 dpi exhibit a gene expression profile that is distinct from exhausted cells. Specifically, we found that genes upregulated in effector CD8^+^ T cells were enriched in the CD8^+^ T cells from PRRSV-infected group, while genes associated with T cell exhaustion signatures were downregulated (**Supplementary Figure 1**). Furthermore, our GSEA of effector CD8^+^ T cells from PRRSV-infected group revealed significant enrichment of genes associated with effector CD8^+^ T cell state during chronic LCMV infection, while these genes were downregulated in the exhausted state. Notably, the gene expression profile of CD8^+^ T cells from the PRRSV-infected group showed a significant enrichment in genes upregulated in effector CD8^+^ T cells at the peak expansion phase (day 8 after LCMV-Armstrong infection) compared to effector CD8^+^ T cells at the contraction phase (day 15 after LCMV-Armstrong infection), indicating a highly active effector state. As expected, these genes were also significantly enriched in effector CD8^+^ T cells at the peak expansion phase (day 8 after LCMV-Armstrong infection) compared to memory CD8^+^ T cells (day 40+ after LCMV-Armstrong infection). GSEA of Hallmark gene sets revealed significant enrichment of CD8^+^ T cells from the PRRSV-infected group in IFN-α response, IFN-γ response and inflammatory response (**Supplementary Figure 2**). Moreover, findings showed that gene sets related to cell division and proliferation (E2F targets, G2/M checkpoint, mitotic spindle assembly) as well as effector metabolic programming (glycolysis, MYC targets, mTORC1 complex) were positively enriched in CD8^+^ T cells from PRRSV-infected group at 21 dpi. Additionally, CD8^+^ T cells from the PRRSV-infected group at 21 dpi showed significant enrichment in three hallmark gene sets associated with T cell activation, acute phase response, and maintenance of effector CD8^+^ T cells during infection.

### 3.9 Temporal quantification of cytotoxic T cell response to PRRSV infection

**Figure 10:**
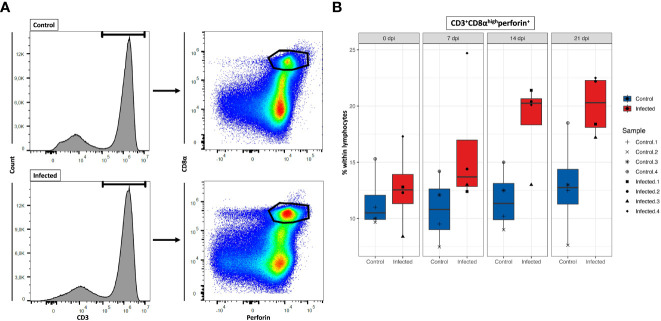
Total number of CD8^+^ T cells at four time points. **(A)** Gating strategy for a representative control and PRRSV-infected animals at 21dpi for the determination of CD8^+^ T cells. **(B)** Total CD8^+^ T cells numbers calculated based on the percentages of CD3^+^CD8α^high^perforin^+^ cells in total lymphocytes measured by FCM.

Characterization of porcine CTLs can be described by the expression of CD3, CD8α and perforin (51). In this study, we investigated the temporal changes of CTLs in response to PRRSV infection, with the aim to provide valuable insights into the role of CTLs in immune response to PRRSV. Here the population of CTLs was defined as CD3^+^CD8α^high^perforin^+^ cells (**Figure 10A**). Mean frequencies of CD3^+^CD8α^high^perforin^+^ remained stable in control group, while they progressively increased in PRRSV-infected group over time. Notably, during the period from 7 dpi to 21 dpi, average increase of CD3^+^CD8α^high^perforin^+^ was approximately 55% in infected groups compared to the control groups. Also, the highest frequencies of CD3^+^CD8α^high^perforin^+^ in PRRSV-infected group were recorded at 21 dpi (mean = 20.1%) (**Figure 10B**).

### 3.10 Differentiation stages of PRRSV-infected CD8β^+^ T cells

**Figure 11:**
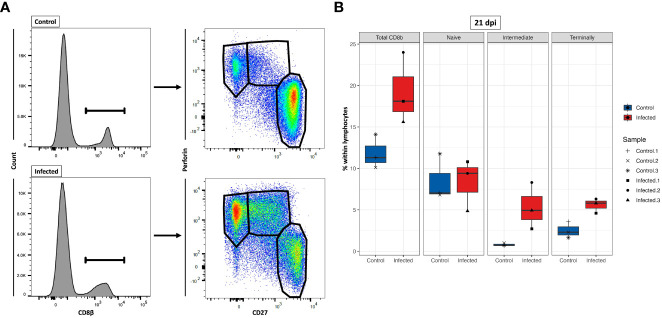
Characterization of three CD8β^+^ T cell subsets at 21 dpi. **(A)** Gating strategy for a representative control and PRRSV-infected animals for the determination of three CD8β^+^ cell subsets: naïve (T_n_; CD8β^+^CD27^+^perforin^-^), intermediate differentiated (T_inter_; CD8β^+^CD27^dim^perforin^+^), and terminally differentiated cells (T_term_; CD8β^+^CD27^-^perforin^high^). **(B)** T_n_ (CD8β^+^CD27^+^perforin^-^), T_inter_ (CD8β^+^CD27^dim^perforin^+^), and T_term_ cells (CD8β^+^CD27^-^perforin^high^) numbers were calculated based on the percentages of subset in total lymphocytes measured by FCM.

Our current understanding of the differentiation stages that porcine CTLs undergo in response to PRRSV infection is limited. Previous studies showed that we can identify these stages by analyzing the expression of CD8β, CD27, and perforin (44, 51, 52). To gain further understanding of the differentiation process upon PRRSV infection, we investigated the differentiation stages of CTLs by analyzing the phenotypic expression of these three markers at 21 dpi (**Figure 11A**). Due to technical issues, two animals (one from each group) had to be excluded from the analysis, resulting in a final sample size of 3 animals in the PRRSV-infected group and 3 animals in the control group. The results showed an 62% increase of total CD8β^+^ T cells in the PRRSV-infected group at 21 dpi, with a mean frequency of 19.2% compared to 11.8% in the control group (**Figure 11B**). Furthermore, the distribution of three distinct subsets of CD8β^+^ T cells differed markedly between the control and PRRSV-infected groups. Although the mean frequencies of naïve cells (T_n_; CD8β^+^CD27^+^perforin^-^) were comparable between the control and PRRSV-infected groups (mean = 8.5% vs. 8.4%), there were notable differences in the distribution of intermediate differentiated (T_inter_; CD8β^+^CD27^dim^perforin^+^) and terminally differentiated cells (T_term_; CD8β^+^CD27^-^perforin^high^) between the two groups. Specifically, in the PRRSV-infected group, we observed a remarkable increase of T_inter_ and T_term_ frequencies, which were over 6.6 and 2.2 times higher than those in the control group, respectively.

## 4 Discussion

In this study, we investigated the gene expression profiles of PBMCs and CD8^+^ T cells after infection with PRRSV strain AUT15-33 over 21 days by identifying the differentially expressed genes and conducting time-course clustering analysis at four time points (0, 7, 14 and 21 dpi) to determine gene expression dynamics of the immune response against PRRSV. To the best of our knowledge, this is the first study which comprehensively describes the time-course of transcriptome responses to PRRSV infection in PBMCs and CD8^+^ T cells.

The findings of this study highlight significant differences in the gene expression patterns of PBMCs and CD8^+^ T cells in response to PRRSV infection. The identification of the highest number of DEGs at different time points in each cell type suggests that the immune response is dynamic and time-dependent. The heterogeneous gene expression patterns observed in PBMCs suggest that different immune cell populations within this mixed population may be responding differently to the infection. This is supported by the observation that the majority of DEGs in PBMCs were specific to each time point, indicating a dynamic and time-dependent response to the infection. In contrast, the observation that the number of DEGs in CD8^+^ T cells increased over time, and that there was more overlap in the DEGs observed at different time points, suggests a more consistent response from this specific cell population. This can be explained by the fact that activated and differentiated CD8^+^ T cells have a more comparable gene expression profile (44, 52). Overall, these findings underscore the complexity and heterogeneity of the immune response to PRRSV infection, and highlight the importance for a more comprehensive understanding of the dynamics of gene expression in different immune cell populations over time. Such understanding could lead to the identification of potential targets for therapeutic intervention to improve the immune response to PRRSV infection.

PPI network analysis of DEGs in PBMCs and CD8^+^ T cells provided valuable insights into the complex immune response mechanisms triggered by PRRSV. The key clusters associated with innate and adaptive immune response, regulation of cytokines, and cell cycle processes suggest that a complex interplay of several immune pathways is involved in combating PRRSV. The observation that the innate immune response in PBMCs begins early at 7 dpi and continues at later time points with the involvement of adaptive immunity and regulation of cell cycle processes highlights the importance of an early and coordinated response against PRRSV, given its ability to suppress innate immunity and thereby delay the adaptive immune response (1, 7, 8). The variation in the number of proteins involved in the immune response processes in CD8^+^ T cells over time suggests that different immune mechanisms come into play at different stages of the infection. The presence of clusters associated with adaptive immune response, immune effector process and cytokine-mediated signaling pathways at 14 dpi and 21 dpi indicates the importance of these pathways in the later stages of the CD8^+^ T response. The observation of clusters associated with T-cell activation, differentiation, adaptive immune response, regulation of cell killing and cytokine production at 21 dpi further highlights the role of CD8^+^ T cells in the immune response against PRRSV. Also, the extensive cell cycle processes observed in CD8^+^ T cells at 21 dpi may reflect their proliferation and differentiation, which are necessary for a robust adaptive immune response against PRRSV.

To better understand changes in immune-related gene expression, we analyzed the most representative genes across five functional categories: transcription factors, effector function and apoptotic genes, cytokine genes, chemokine genes, and co-stimulatory and co-inhibitory genes. Overall, CD8^+^ T cells had a consistently higher number of DEGs compared to PBMCs. By examining PBMC-specific DEGs in infected group, we can derive genes not directly related to CD8^+^ T cells, as they are a subset of PBMCs. These include: *STAT1*, *PLSCR1*, *EGR3*, *HSP09B1*, *CXCL13*, *TNFAIP6*, *TNFRSF1A*, and *TNFRSF17*. Previous research showed that PRRSV infection induces expression of *STAT1* and upregulates some proinflammatory cytokines (53). Moreover, *STAT1* is essential in the IFN-α-activated JAK/STAT signaling pathway and it induces expression of IFN-stimulated genes (ISGs) which are important in innate immunity against viral infection (54, 55). Among these, we found three ISGs (*ISG12(A)*, *ISG15*, *ISG20*) significantly upregulated from 7 dpi onwards, in accordance with findings that demonstrate the antiviral activity of ISGs (56–58) and essential role of the heat-shock proteins (HSPs) in signal transduction pathways, which has beneficial effects such as inhibition of virus replication and activation of an antiviral immune response (59). Also, PRRSV-infection is usually accompanied by increased temperature in both young and old pigs (1) and these stress conditions (fever and viral infection) can cause upregulation of the HSPs inside the cell (60). Our findings regarding the *HSP90B1* marker align with a previous study that showed upregulation of this gene in porcine lung after PRRSV infection (30). Interestingly, our analysis also revealed the upregulation of *ETV7* and *EGR3* in PBMCs following PRRSV infection. *ETV7* is a negative regulator of the type I IFN response, particularly on antiviral ISGs (40), while *EGR3* suppresses T-cell activation and the expression of *IFNGR1*, contributing to the anti-inflammatory effects of type I IFNs (41, 42).

During a PRRSV infection, the body’s natural defenses against pathogens are weakened. This includes a reduction in the cytotoxic activity of NK cells, which play a key role in the early immune response (7, 61, 62). Based on our assumption that the early cell-mediated immune response to PRRSV in PBMCs at 7 dpi is primarily driven by NK cells (1), our results indicate that this suppression may not be complete, as we observed an upregulation of effector genes such as granzymes (GZMA, GZMB, GZMK), killer lectin like receptor (KLRD1), and BCL2L14 at 7 dpi. These findings can be explained by the crucial role of STAT1 in innate immunity, which is critical for NK cell cytotoxic activity that is independent of IFN signaling (63). It is worth noting that these markers may also be induced by other immune cells, such as CD8^+^ T cells. However, some of these markers were not expressed in CD8^+^ T cells, and CD8^+^ T cells expressed additional markers that are not present in PBMCs.

The results on cytokine and chemokine expression in PBMCs of the present study exhibit a degree of concurrence with the outcomes of the prior research, albeit with some variations. A previous study found that PRRSV-infected gilts showed a significant increase of CCL2 and IFN-α in serum at 2 dpi and 6 dpi, whereas IFN-γ was increased significantly at 2 dpi only. However, other analytes including IL1β, IL8, IL12, IL4 and IL10 did not significantly differ over time (64). Although our study differs in the design and methodology, there may be some commonalities that allow for certain results to be compared. Similar to aforementioned study, the expression of IL1β, IL8, IL12, IL4 and IL10 did not significantly differ over time between PRRSV-infected and control gilts. In contrast, our study revealed a significant upregulation of CCL2 at 21 dpi only, while no significant increases were observed in IFN-α and IFN-γ expression. These differences in expressions can be attributed to a number of factors, such as earlier days of sample collection as well as selected methods of studies. However, our findings align with prior research indicating that PRRSV infection decreases the production of IFN-α (7, 65, 66) and delays the IFN-γ response, while also reducing its effects (65, 67, 68). Notably, PRRSV-infected gilts did not show any IFN-α production, which could be beneficial since high levels of IFN-α have been associated with increased fetal mortality (69). Additionally, our findings regarding IFN-α, IL1β, IL8 and CSF2 are consistent with a previous gene expression study that demonstrated no significant upregulation of these innate markers in animals infected with PRRSV, regardless of whether the infection is persistent or non-persistent (70). Our findings are consistent with another study that showed a downregulation in the expression of Th2 markers (*IL4*, *IL5*, *IL13*, *IL25*) and innate immunity markers (*IL1β*, *IL6*, *IL8*, *IFNα*) in PBMCs isolated from pigs at week 5 after MLV vaccination and subjected to *in vitro* restimulation with PRRSV strain VR-2332 (71). It is known that PRRSV strongly induces upregulation of chemokines such as *CCR1*, *CCR2*, *CCL8*, *CXCL9*, *CXCL10*, *CXCL13*, and *CXCR6* in the lungs of PRRSV-infected animals (30). Our study confirms these previous findings but also shows that the expression of these chemokines varies throughout the entire infection period, with the highest expression observed at 21 dpi. These variations may mirror the complex interplay between PRRSV and the host immune response. Overall, our study shows that PRRSV infection in gilts is associated with alterations in the cytokine and chemokine expression, with some similarities and differences compared to previous research, indicating a potential decrease in IFN-α production, delayed IFN-γ response, downregulation of innate immunity markers, and upregulation of certain chemokines.

Upon virus infection activated naïve CD8^+^ T cells proliferate and differentiate into virus-specific effector CD8^+^ T cells that can effectively eliminate virus and virus-infected cells (72). Their effector activity is based on the production of effector cytokines and granule-associated proteases (73–75). When looking at CD8^+^ T cells only, we observed the upregulation of genes associated with later stages of porcine CD8^+^ T-cell differentiation along the time-course. For example, transcription factor genes such as *PRDM1* (Blimp-1), *EOMES*, and *TBX21* (*T-bet*) were highly upregulated at 14 dpi and 21 dpi, which fits well with a recent study showing the upregulation of *T-bet* and *EOMES* following PRRSV infection (76). Moreover, genes linked to cytolytic activity including granzymes (*GZMA*, *GZMB*, *GZMK*), perforin (*PRF1*), fas ligand (*FASLG*), killer cell lectin like receptors (*KLRK1*, *KLRD1*), and a natural killer cell granule protein 7 (*NKG7*) were significantly upregulated at later time points (14 and 21 dpi) (47). Notably, the strongest expression was observed at 21 dpi, which further supports the effector function of CD8^+^ T cells.

Several research papers suggest that expression of co-inhibitory molecules such as *PDCD1* (PD-1), *HAVCR2* (Tim-3), *CTLA4*, and *LAG3* correlates with the activated and more differentiated state of CD8^+^ T cells in viral infection (77–79). In our study, we found high expression of *PDCD1* (PD-1) and *CTLA4* from 7 dpi to 21 dpi, with the strongest expression at 21 dpi. Furthermore, both *HAVCR2* and *LAG3* showed the highest upregulation at 21 dpi. However, prolonged expression of these markers during chronic infection contributes to the exhaustion of CD8^+^ T cells (49, 50). To investigate the potential for CD8^+^ T cell exhaustion during PRRSV infection, we used GSEA to compare gene expression profiles of CD8^+^ T cells from PRRSV-infected group at 21 dpi with those of exhausted cells. Our GSEA results indicate that CD8^+^ T cells from PRRSV-infected group at 21 dpi exhibit a gene expression profile that is distinct from exhausted cells. In particular, these cells showed significant enrichment in effector CD8^+^ T cell gene sets in general as well as during chronic LCMV infection. Upon antigen stimulation, effector CD8^+^ T cells are known to exhibit a high degree of proliferative capacity as a key feature (80). Also, the metabolic programming of these cells rely on aerobic glycolysis (81, 82), while exhausted CD8^+^ T cells suppress AKT activation and mTOR activity, resulting in a metabolic switch from glycolysis to fatty acid oxidation (FAO) (83, 84). The positive enrichment of gene sets related to cell division and proliferation in CD8^+^ T cells from PRRSV-infected group at 21 dpi, suggests that these cells can undergo rapid proliferation in response to viral infection. Moreover, these cells were enriched in gene sets associated with effector metabolic programming, such as glycolysis, MYC targets, and mTORC1 complex, which suggests that they have the necessary metabolic pathways to support their effector function. In addition, the enrichment of hallmark gene sets associated with T cell activation, acute phase response, and maintenance of effector CD8^+^ T cells during infection, further supports the notion that CD8^+^ T cells from the PRRSV-infected group at 21 dpi have an activated effector phenotype.

Another distinctive feature of effector CD8^+^ T cells is the capacity to secrete inflammatory cytokines and chemokines that work together to promote the immune response against viral infections (85). In contrast to gene expression profile of PBMCs derived from PRRSV-infected animals, within CD8^+^ T cells we found a very strong expression of several cytokine genes (*IL10*, *IL2RB* (CD122), *IFNG* (IFN-γ), *IL21R*, *IL15RA*, *IL13RA1*) at 14 and 21 dpi. Our findings are consistent with a previous study that identified CD8^+^ T cells as the primary producers of IFN-γ in the lungs of PRRSV-vaccinated animals (86). At the peak of immune response in mice, CD8 T cells are main producers of IL10 at the peripheral sites, whereas they transit to IL10^-^CD8^+^ T cells during later phase (87–89). IL10^+^CD8 T cells also produce higher amount of granzyme B, IFN-γ and TNF-α than IL10^-^CD8^+^ T cells (89). Our results are consistent with some of these findings, as we observed high expression of IL10 at 14 dpi but not at later time points. However, we found that only GZMB were higher produced, while other granzymes and IFN-γ were more produced at later time point in presumably IL10^-^CD8^+^ T cells. Production of IL10 in CD8^+^ T cells is directly correlated with level of *PRDM1* expression (Blimp-1) during acute viral infection (90). A previous study demonstrated that Blimp-1 expression in CD8^+^ T cells might also be induced by other cytokines such as IL21 (91, 92), which is crucial for long-term maintenance of functionality of CD8^+^ T cell in chronic viral infections such as LCMV in mice (93, 94). In the absence of IL21, CD8 T cells may acquire a more exhausted state, unable to exhibit their cytolytic properties. Our study showed that CD8^+^ T cells from PRRSV infected animals expressed *IL21R* at 14 dpi only, whereas Blimp-1 was expressed at both 14 dpi and 21 dpi, suggesting possible role of Blimp-1 in regulating the CD8^+^ T-cell response to PRRSV.

Our study identified a high number of chemokines and chemokine receptors in CD8^+^ T cells from the PRRSV-infected group. We found that chemokine and chemokine receptor genes such as *CXCR3* and its two ligands (*CXCL9, CXCL10*) were particularly highly expressed at 21 dpi. Together with its ligands, *CXCR3* is known to be highly expressed on activated CD8^+^ T cells (95). Moreover, *CXCL10* was continuously upregulated from 7 dpi to 21 dpi, which can be explained by the fact that *CXCL10* promotes generation of CD8^+^ effector cells (96). Interestingly, we found that the transcripts of some genes including *CCR1*, *CCL2*, *CCL4*, *CCL8*, *CXCR3*, *CXCR6*, *CXCL9*, and *CXCL16* were significantly elevated at 21 dpi only. This suggests that these genes may play a role in the later stages of the CD8^+^ T-cell response to PRRSV. Another interesting finding was the upregulation of *CX3CR1* at 7 and 14 dpi. *CX3CR1* is expressed on virus-specific CD8^+^ T effector cells (48), which suggests that these cells may be important in the early stages of the immune response to PRRSV.

Upon encountering a virus, naive CD8^+^ T cells are activated and undergo a process of rapid proliferation and differentiation, resulting in the generation of a heterogeneous pool of effector CD8^+^ T cells that play a crucial role in the host’s immune response against the pathogen (97). Various studies have explored the role of CD8^+^ T cells in the immune response to PRRSV infection. Peripheral blood CD8^+^ T cells have been found to proliferate upon restimulation *in vitro* 21 dpi and gain the ability to kill PRRSV-infected macrophages 49 dpi (98). Other studies suggest that CD8^+^ T cells may play an important role in controlling a PRRSV infection at the site of infection, particularly in the lung and bronchoalveolar lavage (99, 100). Infected pigs have shown a higher percentage of CD8^+^ T cells and higher levels of IFN-γ-producing cells in their bronchoalveolar lavage fluid compared to control pigs at 5 weeks post-infection (101). PRRSV-specific T cells have also been observed as early as 2 weeks post-infection, but the effectiveness of CD8^+^ T cells in controlling primary PRRSV infection is still uncertain, as anti-PRRSV-targeted CTLs were only detected after clearance of viremia (98). Nevertheless, recent research has demonstrated that during late gestation CD8α^pos^CD27^dim^ early effector CD8β^+^ T cells exhibit the strongest response to infection with the two PRRSV-1 strains compared to other investigated lymphocyte subsets (102). Our findings are consistent with these results. Using flow cytometry, we observed a progressive increase in the population of CD8^+^ T cells characterized by CD3^+^CD8α^high^perforin^+^ expression following PRRSV infection. This population peaked at 21 days post-infection, indicating an ongoing immune response. Additionally, we observed an increase in total CD8β^+^ T cells in the PRRSV-infected group at 21 dpi, with notable differences in the distribution of intermediate and terminally differentiated cells compared to the control group. These results suggest that the PRRSV infection induces differentiation of CTLs, with a shift towards more differentiated subsets. Taken together, these findings indicate that PRRSV infection leads to a significant expansion and differentiation of CTLs, which could play an important role in controlling the virus. Although our study provided important insights into the role of CD8^+^ T cells in the immune response to PRRSV infection, we recognize that our analysis was limited by the unavailability of material to use CD8α complementing with CD4 or CD8β markers for time course analysis. Thus, additional studies using alternative markers are warranted to further elucidate the immune response to PRRSV infection.

Our findings demonstrate two key insights about the CD8^+^ T-cell response to PRRSV infection. First, the general induction of an adaptive immunity through activation of CD8^+^ T cells, with this response constantly increasing and reaching its peak at 21 dpi. Second, from 14 dpi to 21 dpi CD8^+^ T cells acquired a more differentiated profile characterized by stronger effector functions and cytolytic activity. To gain insights into the transcriptional regulation of immune-response genes to PRRSV infection, we performed temporal clustering analysis of DEGs in PBMCs and CD8^+^ T cells from infected animals. In PBMCs, the acute peak at 7 dpi in cluster 1 and the involvement of signaling pathways for innate immune response to viral infection suggest an early activation of host immune defense mechanisms. In their study, Wilkinson et al. found that *CCNB1*, *ISG20*, and *TNFSF10* were upregulated in the whole blood of pregnant gilts at 6 days post-infection with PRRSV-2 (32). Interestingly, these genes were also identified in cluster 1 of our analysis, which also exhibited high expression of *OAS1* and *OAS2*, members of the 2′-5′ oligoadenylate synthetase (OAS) family known to be rapidly induced in response to viral infections (103). In addition, studies in mice have shown that *OAS1* and *OAS2* expression can be enhanced in the lungs after influenza A infection or pathogen-associated molecular pattern stimulation (PAMPs) (104). Cluster 1 also included *OAS2*, *ISG15*, *ISG20*, *USP18* and *MX1*, which were found to be upregulated in the lungs of swine infected with H1N1 swine influenza virus (105). In addition, the *MX1* marker was found to be upregulated in uterine endothelium with adherent placental tissue from PRRSV infected gilts (31). Also, expression of *MX1*, *ISG20*, and *IFIT3* in whole blood from PRRSV-inoculated gilts correlated positively with low fetal mortality at 6 dpi (32). *DDX60*, also present in this cluster, is known to be elevated after viral infection and promotes RIG-I-like receptor-mediated signaling (106). Furthermore, previous studies have demonstrated that the expression levels of ISGs, including *DDX60*, *ISG20*, and *USP18*, were significantly upregulated in whole blood after PRRSV infection (107). Overall, the upregulation of cluster 1 genes in response to PRRSV infections may represent a conserved and critical aspect of the host antiviral response. Cluster 2 increased from 21 dpi and was associated with biological processes such as humoral immune response and complement activation, indicating a crucial role of humoral immunity in response to PRRSV infection. In contrast, the downregulation of genes involved in blood coagulation and receptor-mediated endocytosis in cluster 3 suggests that PRRSV may also evade host immune response by interfering with these biological processes. Supporting evidence from other studies suggests that PRRSV infection involves receptor-mediated endocytosis and replication within host cells (108–110). In particular, infected pigs have been shown to develop a rapid humoral response, but the early development of sub- or non-neutralizing antibodies can enhance viral attachment and internalization through Fc receptor-mediated endocytosis, a phenomenon known as antibody-dependent enhancement (ADE) (111). Moreover, confocal microscopy studies have demonstrated the receptor-mediated endocytosis of PRRSV virions into endosomes (112). Additionally, several studies have highlighted the correlation of blood coagulation and complement cascade pathways with PRRSV infection and vaccination responsiveness (113). These pathways play important roles in the first line of defense against pathogens and the regulation of inflammatory responses (114). Furthermore, early changes in blood transcriptional modules (BTMs) associated with the neutralizing antibody response have included the blood coagulation, platelet activation, and complement activation (29). The KEGG analysis revealed significantly enriched pathways in clusters 1 and 2, with cluster 1 showing involvement in various signaling pathways for innate immune response to viral infection, including RIG-I-like, toll-like and NOD-like receptor pathways, and cluster 2 being associated with pathways involved in bacterial infections such as *Staphylococcus aureus* and *pertussis*. The RIG-I-like receptor signaling pathway is activated by viral infections and initiates an antiviral innate immune response (115, 116). Additionally, the Toll-like and NOD-like receptor signaling pathways, which are also part of pattern recognition receptors (PRRs), play a crucial role in the innate immunity and assist in activation of the adaptive immunity (117). In conclusion, our study suggests that PRRSV infection elicits early activation of host immune defense mechanisms through innate immune response signaling pathways and plays a crucial role in humoral immunity, while also potentially evading host immune response by interfering with genes involved in blood coagulation and receptor-mediated endocytosis.

Time-clustering analysis of DEGs of CD8^+^ T cells revealed four clusters, which shed light on the molecular processes in CD8^+^ T cells following PRRSV infection. Genes enriched in cluster 1 and 2 showed that from 7 dpi CD8^+^ T cell undergo continuous massive cell division and proliferation in response to PRRSV. Notably, the gene *MKI67*, which encodes the marker of proliferation Ki67 (118, 119), was among these genes in cluster 2. In a previous study, we demonstrated that *MKI67* is upregulated in porcine intermediate and terminally differentiated but not in naïve CD8^+^ T-cell subsets (44). Also, the expression of *MKI67* is in accordance with previous research suggesting that PBMCs of PRRSV-infected animals induce the number of proliferating CTLs after *in vitro* restimulation from 14 dpi (98). Therefore, genes in cluster 2 probably contribute to the early cell transformation of CD8^+^ T cells after encountering PRRSV. At 7 dpi, cluster 3 revealed downregulated genes necessary for metabolic switching from fatty acid oxidation, which is typical for naïve CD8^+^ T cells (120). This suggests that CD8^+^ T cells undergo metabolic reprogramming in response to PRRSV infection. Lastly, cluster 4 showed that CD8^+^ T cells from 21 dpi increase the chemotaxis and chemokine activity, suggesting a crucial role of these cells in the immune response to PRRSV infection.

In conclusion, our study uncovered the dynamic gene expression patterns of PBMCs and CD8^+^ T cells during PRRSV infection over the course of 21 days. We observed that the initial innate immune response in PBMCs peaked at 7 dpi, while the adaptive immune response in CD8^+^ T cells was most prominent at 21 dpi, marked by the generation of highly differentiated CD8^+^ T cells with potent effector and cytolytic capabilities. Our findings shed light on the complex transcriptional changes and key players involved in the immune response to PRRSV and provide a valuable resource for the identification of biomarkers for PRRSV diagnosis and improved understanding of PRRS pathogenesis.

## Data availability statement

The datasets presented in this study can be found in online repositories. The names of the repository/repositories and accession number(s) can be found below: https://www.ncbi.nlm.nih.gov/, PRJNA880682.

## Ethics statement

The animal study was reviewed and approved by institutional ethics and animal welfare committee (Vetmeduni Vienna) and the national authority according to §§26ff. of Animal Experiments Act, Tierversuchsgesetz in Austria – TVG 2012 (BMWFW-2021-0.117.108).

## Author contributions

EL, AL and AS designed the project. TR provided the virus for the experimental infection. HK performed sample collection. SS and MeS performed lymphocyte isolation. MaS and EL organized magnetic-activated cell sorting. EL performed RNA isolation and quality assessment. RE and EL prepared libraries of the samples. EL performed in-depth bioinformatic analysis. NP advised on the most suitable bioinformatic analysis. EL and AS analyzed the experiments and wrote the manuscript. CP assisted with the interpretation of the data. All authors contributed to the article and approved the submitted version.
