# Supplementary figures and images for: Gene expression of peripheral blood mononuclear cells and CD8+ T cells from gilts after PRRSV infection
Source: Front Immunol. 2023 Jun 20;14:1159970. doi: 10.3389/fimmu.2023.1159970 (PMC10318438; doi:10.3389/fimmu.2023.1159970)

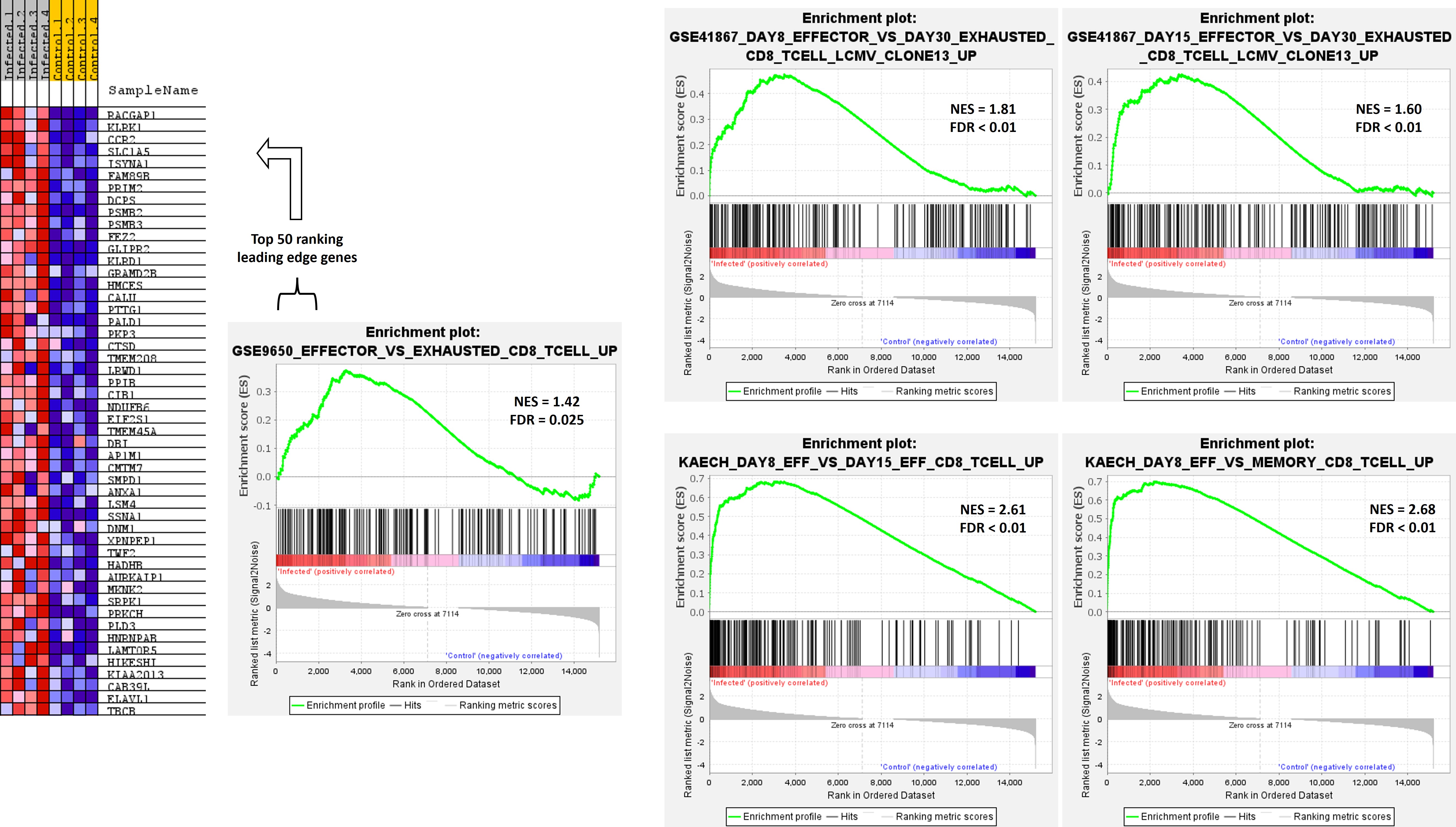

Supplement: Supplementary file 1 [file Image_1.jpeg]

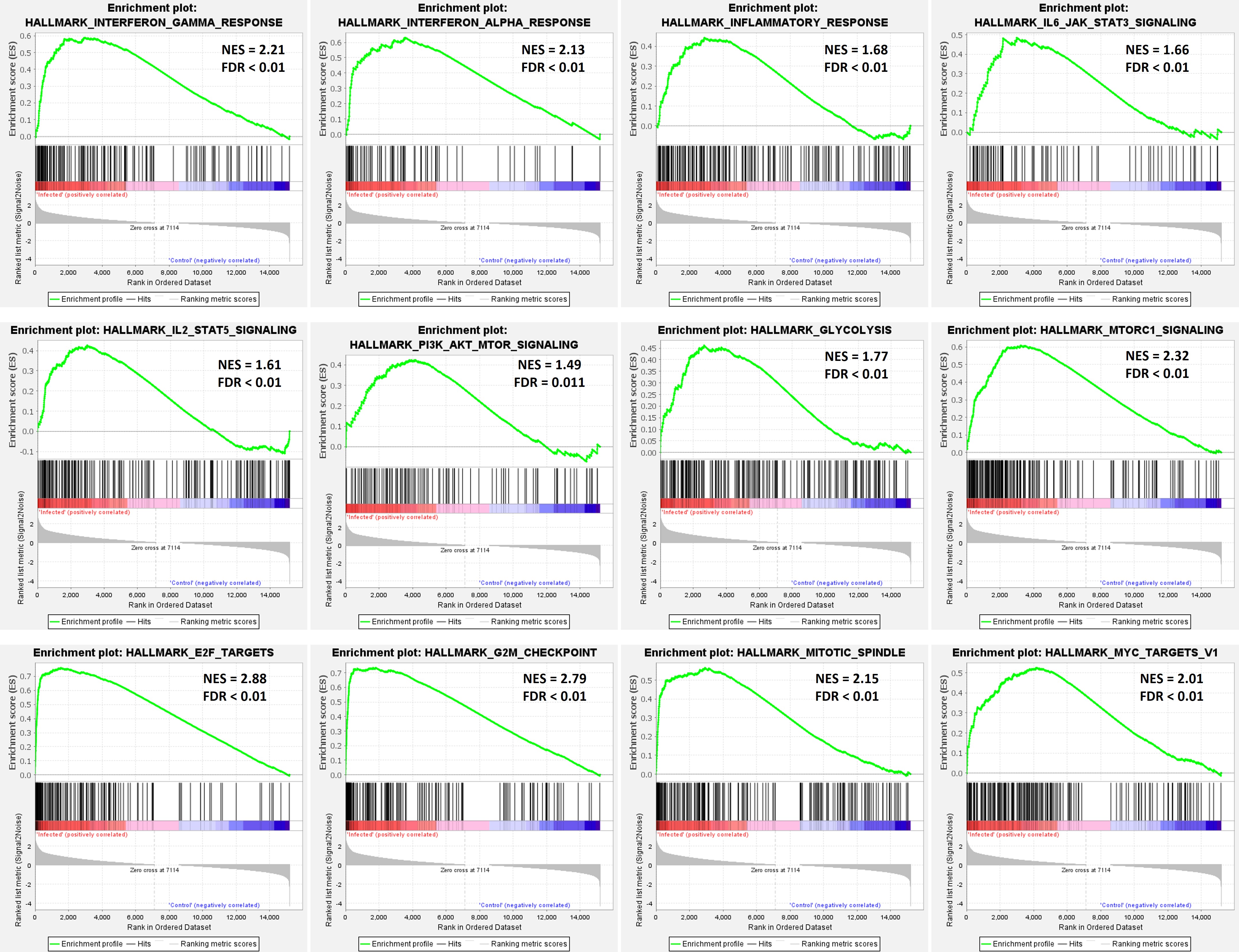

Supplement: Supplementary file 2 [file Image_2.jpeg]
