# Supplementary material for: Gene expression of peripheral blood mononuclear cells and CD8+ T cells from gilts after PRRSV infection
Source: Front Immunol. 2023 Jun 20;14:1159970. doi: 10.3389/fimmu.2023.1159970 (PMC10318438; doi:10.3389/fimmu.2023.1159970)
Supplement: Supplementary file 7 [file DataSheet_1.pdf]

Table of contents

1. Reads summary 3
    1.1 Summary statistics 3
    1.2 Read length distribution 5
2. QC for sequencing reads 5
    2.1 Coverage 5
    2.2 Ambiguous base-content 6
    2.3 Nucleotide contributions 8
    2.4 GC-content 8
    2.5 Quality distribution 10
    2.6 Enriched 5-mer 11
    2.7 Sequence duplication levels 12

1. Reads summary

1.1 Summary statistics

The table is based on 64 samples.

Sample name	Data sets (#)	Reads (#)	Paired reads (%)	Bases (#)
190979_S1	1	51.860.276	100	7.779.041.400
190980_S2	1	57.639.418	100	8.645.912.700
190981_S3	1	76.585.178	100	11.487.776.700
190982_S4	1	74.909.674	100	11.236.451.100
190983_S5	1	84.211.104	100	12.631.665.600
190984_S6	1	83.920.234	100	12.588.035.100
190985_S7	1	84.363.200	100	12.654.480.000
190986_S8	1	50.572.368	100	7.585.855.200
190987_S9	1	72.211.908	100	10.831.786.200
190988_S10	1	103.284.056	100	15.492.608.400
190989_S11	1	76.216.542	100	11.432.481.300
190992_S12	1	85.664.610	100	12.849.691.500
190993_S13	1	66.883.100	100	10.032.465.000
190994_S14	1	70.965.070	100	10.644.760.500
190995_S15	1	76.277.292	100	11.441.593.800
190996_S16	1	68.856.782	100	10.328.517.300
190997_S17	1	90.157.680	100	13.523.652.000
190998_S18	1	118.116.462	100	17.717.469.300
190999_S19	1	79.276.516	100	11.891.477.400
191000_S20	1	67.107.218	100	10.066.082.700
191001_S21	1	85.524.584	100	12.828.687.600
191002_S22	1	101.658.880	100	15.248.832.000
191003_S23	1	106.818.522	100	16.022.778.300
191004_S24	1	91.417.592	100	13.712.638.800
191005_S25	1	89.618.970	100	13.442.845.500
191006_S26	1	110.405.300	100	16.560.795.000
191007_S27	1	103.584.542	100	15.537.681.300
191008_S28	1	76.751.694	100	11.512.754.100
191009_S29	1	102.051.848	100	15.307.777.200
191010_S30	1	95.751.286	100	14.362.692.900
191011_S31	1	88.571.064	100	13.285.659.600
191012_S32	1	88.240.996	100	13.236.149.400

Sample name	Data sets (#)	Reads (#)	Paired reads (%)	Bases (#)
191013_S33	1	94.920.068	100	14.238.010.200
191014_S34	1	88.025.888	100	13.203.883.200
191015_S35	1	93.548.336	100	14.032.250.400
191016_S36	1	100.695.730	100	15.104.359.500
191017_S37	1	101.512.444	100	15.226.866.600
191018_S38	1	84.326.668	100	12.649.000.200
191019_S39	1	100.288.534	100	15.043.280.100
191020_S40	1	106.756.214	100	16.013.432.100
191021_S41	1	94.438.202	100	14.165.730.300
191022_S42	1	116.877.504	100	17.531.625.600
191023_S43	1	75.470.924	100	11.320.638.600
191024_S44	1	93.734.350	100	14.060.152.500
191025_S45	1	130.251.246	100	19.537.686.900
191026_S46	1	111.715.376	100	16.757.306.400
191027_S47	1	118.691.480	100	17.803.722.000
191028_S48	1	82.844.912	100	12.426.736.800
191029_S49	1	106.984.422	100	16.047.663.300
191030_S50	1	105.016.498	100	15.752.474.700
191031_S51	1	115.222.302	100	17.283.345.300
191032_S52	1	94.211.386	100	14.131.707.900
191033_S53	1	105.846.956	100	15.877.043.400
191034_S54	1	96.994.300	100	14.549.145.000
191035_S55	1	116.610.434	100	17.491.565.100
191036_S56	1	87.186.660	100	13.077.999.000
191037_S57	1	112.596.746	100	16.889.511.900
191038_S58	1	119.546.242	100	17.931.936.300
191039_S59	1	130.884.680	100	19.632.702.000
191040_S60	1	119.395.784	100	17.909.367.600
191041_S61	1	110.077.048	100	16.511.557.200
191042_S62	1	110.190.516	100	16.528.577.400
191043_S63	1	113.019.250	100	16.952.887.500
191044_S64	1	107.472.130	100	16.120.819.500
Minimum	1,00	50.572.368,00	100,00	7.585.855.200,00
Median	1,00	94.324.794,00	100,00	14.148.719.100,00
Maximum	1,00	130.884.680,00	100,00	19.632.702.000,00
Mean	1,00	94.137.924,94	100,00	14.120.688.740,62
Standard deviation	0,00	18.224.138,70	0,00	2.733.620.804,31

1.2 Read length distribution

The box plot is based on 64 samples.

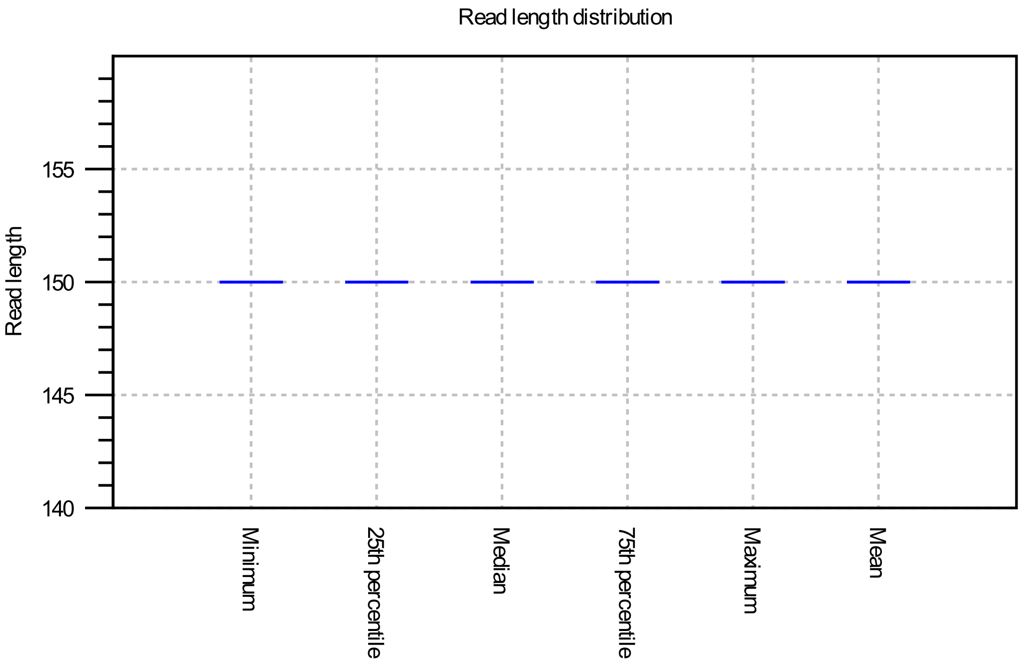

2. QC for sequencing reads

2.1 Coverage

Summarizes the number of sequences that support (cover) the individual base positions.
p - r: The base positions in the read
y: the mean number of sequences covering the respective base positions normalized to the total number of sequences

The box plot is based on 64 samples.

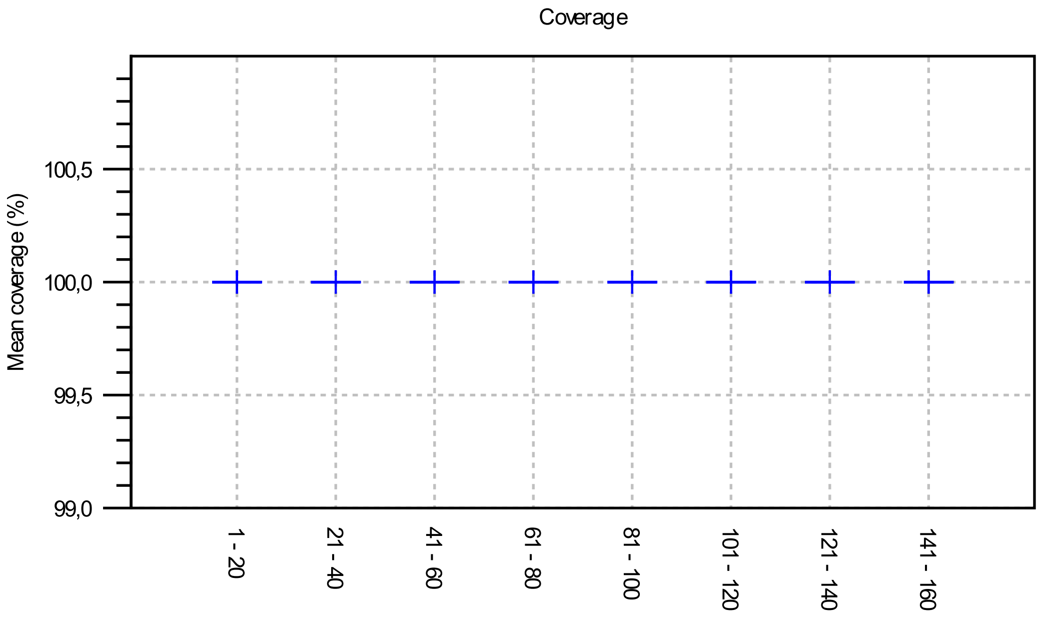

2.2 Ambiguous base-content

Ambiguous base-content per-sequence

Summarizes the distribution of N-contents. The N-content of a sequence is calculated as the number of ambiguous bases compared to all bases.
x: p% - r%: percentage range
y: the number of sequences featuring particular N-percentages normalized to the total number of sequences

The box plot is based on 64 samples.

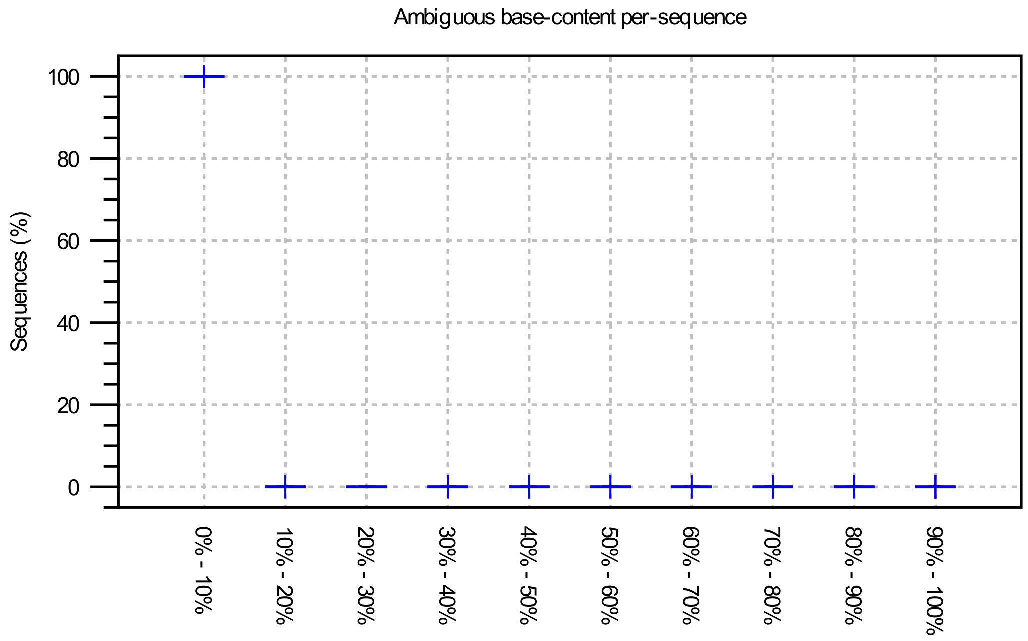

Ambiguous base-content per-base

Summarizes the cumulative coverage of ambiguous bases.
p - r: The base positions in the read
y: the mean number of ambiguous bases observed at the base positions normalized to the total number of bases observed at those positions

The box plot is based on 64 samples.

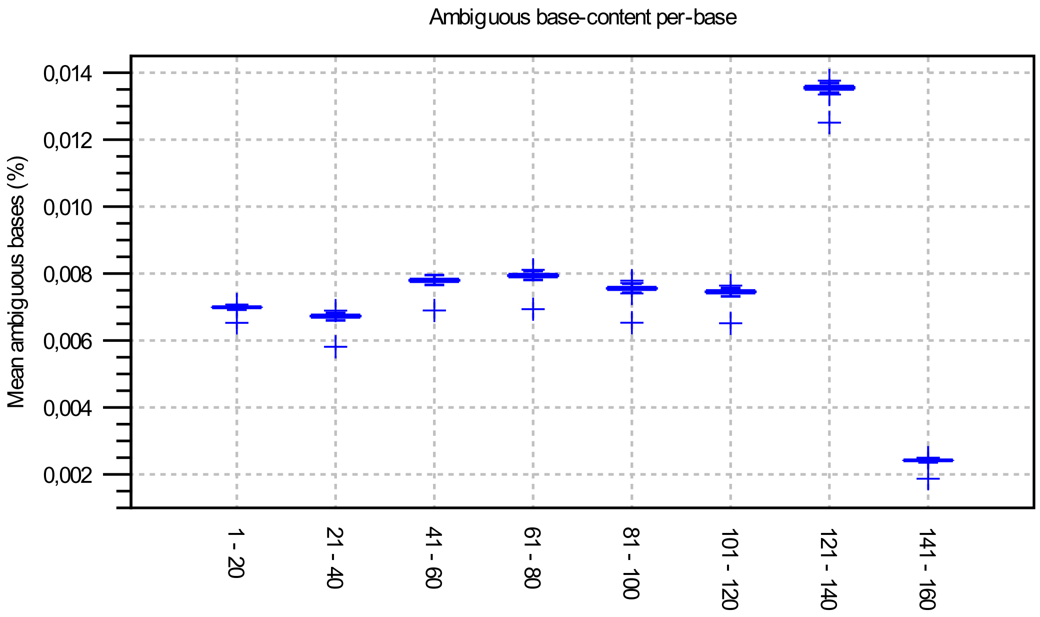

2.3 Nucleotide contributions

Summarizes the coverages for the four DNA nucleotides.
p - r: The base positions in the read
y: the mean number of nucleotides observed per type normalized to the total number of nucleotides observed at those positions

The box plot is based on 64 samples.

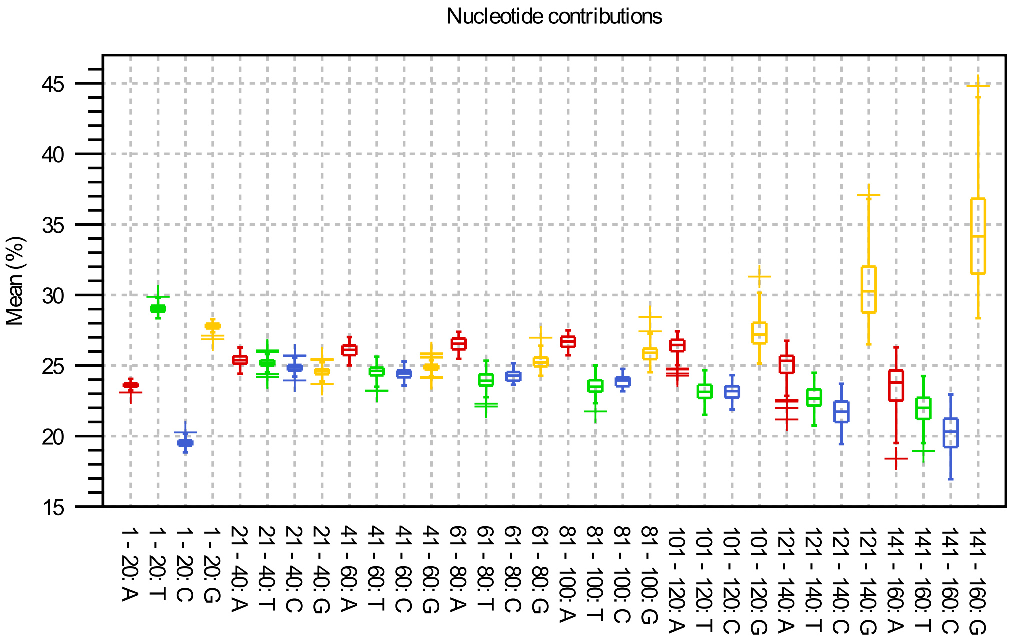

2.4 GC-content

GC-content per-sequence

Summarizes the distribution of GC-contents. The GC-content of a sequence is calculated as the number of GC-bases compared to all bases (including ambiguous bases).
x: p% - r%: percentage range
y: the number of sequences featuring the particular GC-percentage range normalized to the total number of sequences

The box plot is based on 64 samples.

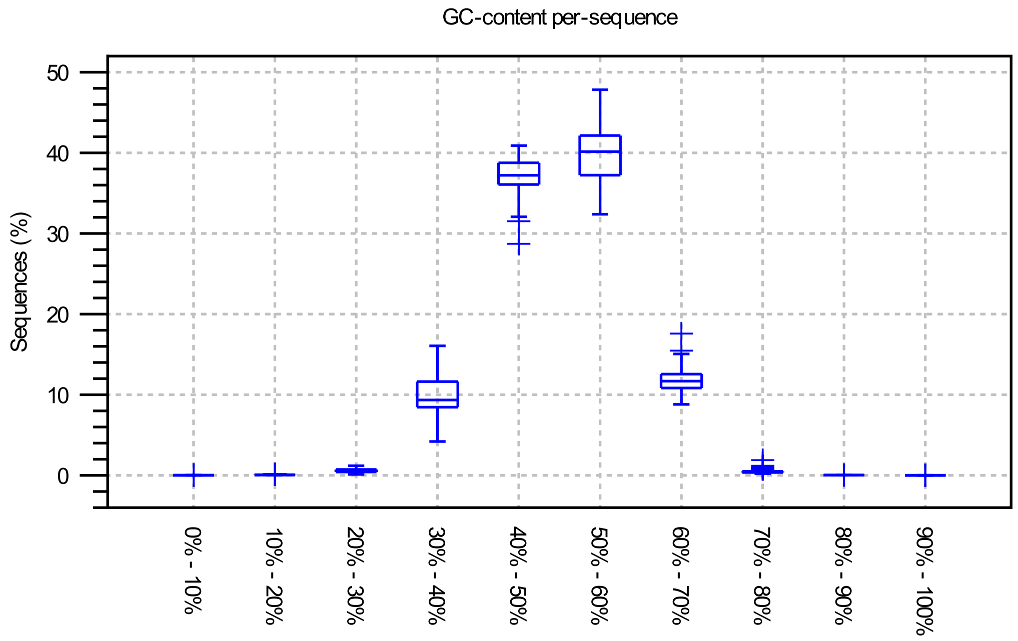

GC-content per-base

Summarizes the cumulative coverage of G- and C-bases.
p - r: The base positions in the read
y: the mean number of G- and C-bases observed at the base positions normalized to the total number of bases observed at those positions

The box plot is based on 64 samples.

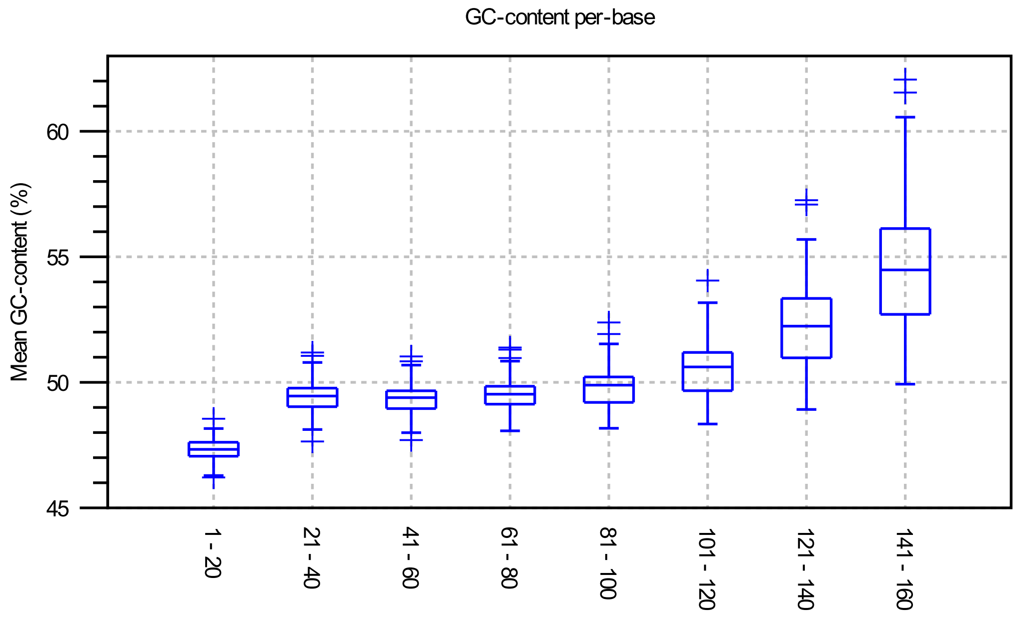

2.5 Quality distribution

Quality distribution per-sequence

Summarizes the distribution of average sequence quality scores. The quality of a sequence is calculated as the arithmetic mean of its base qualities.
x - y: PHRED range
y: the number of sequences observed at that qual. score normalized to the total number of sequences

The box plot is based on 64 samples.

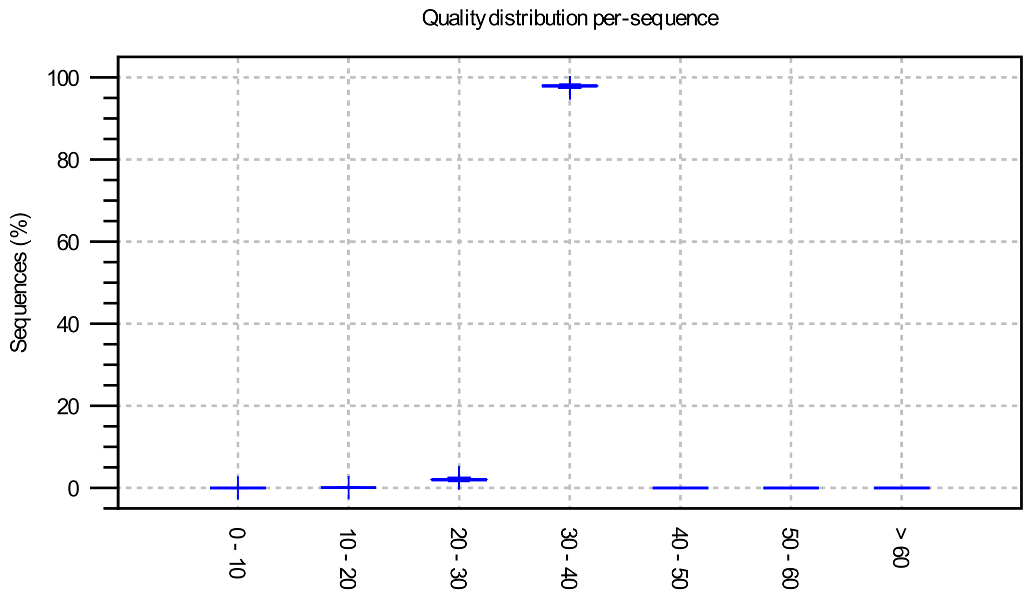

Quality distribution per-base

Summarizes the base-quality distribution along the base positions.
p - r: The base positions in the read
y: the mean of the median of quality scores observed at the base positions

The box plot is based on 64 samples.

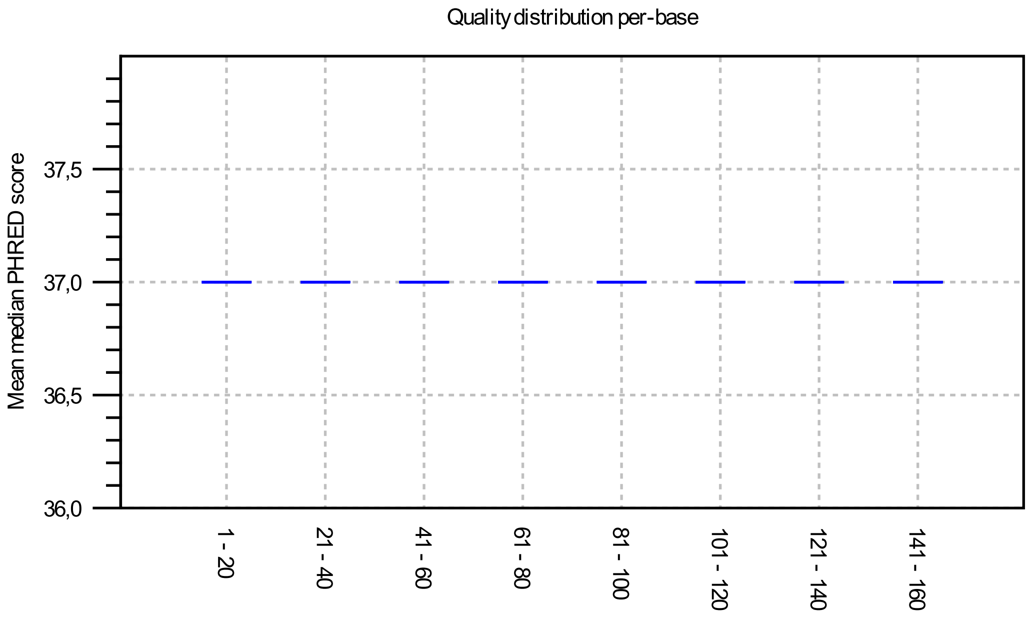

2.6 Enriched 5-mer

Summarizes the most-overrepresented 5-mer in the different types of reads. The over-representation of a 5-mer is calculated as the ratio of the expected and observed 5-mer frequency. The expected frequency is calculated as product of the empirical nucleotide probabilities that make up the k-mer. (5-mers that contain ambiguous bases are ignored)
p - r: The base positions in the read
y: the mean number of times the most enriched 5-mer has been observed at the base positions normalized to all 5-mers observed at those positions in the different types of reads

The box plot is based on 64 samples.

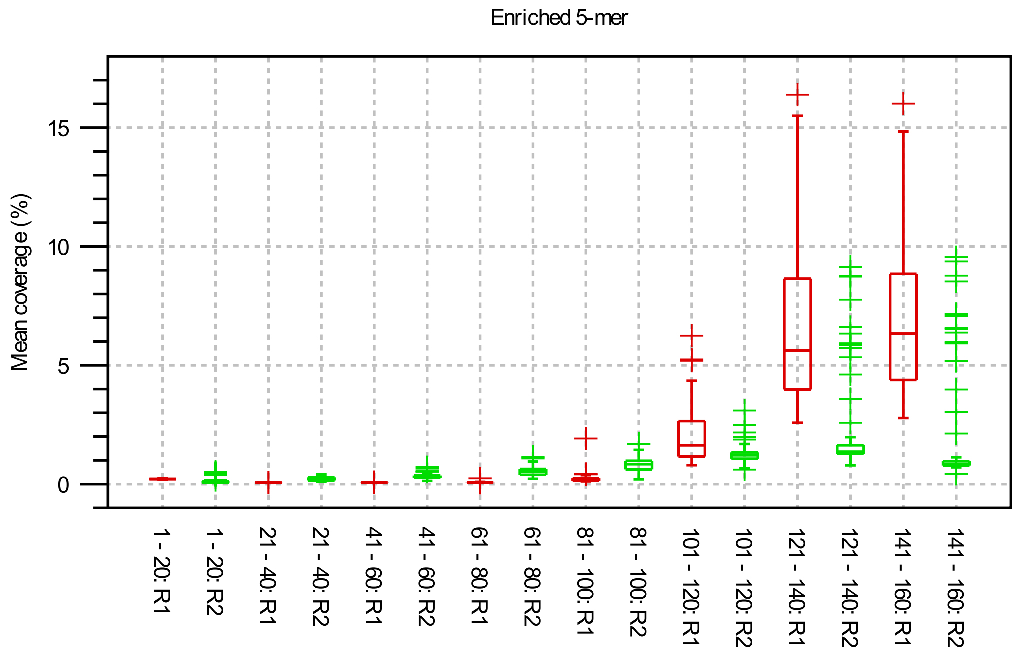

2.7 Sequence duplication levels

Summarizes the duplication level distribution. Duplication levels are simply the count of how often a particular sequence has been found.
x - y: sequence duplication range
y: the number of sequences that have been found that many times normalized to the number of unique sequences

The box plot is based on 64 samples.

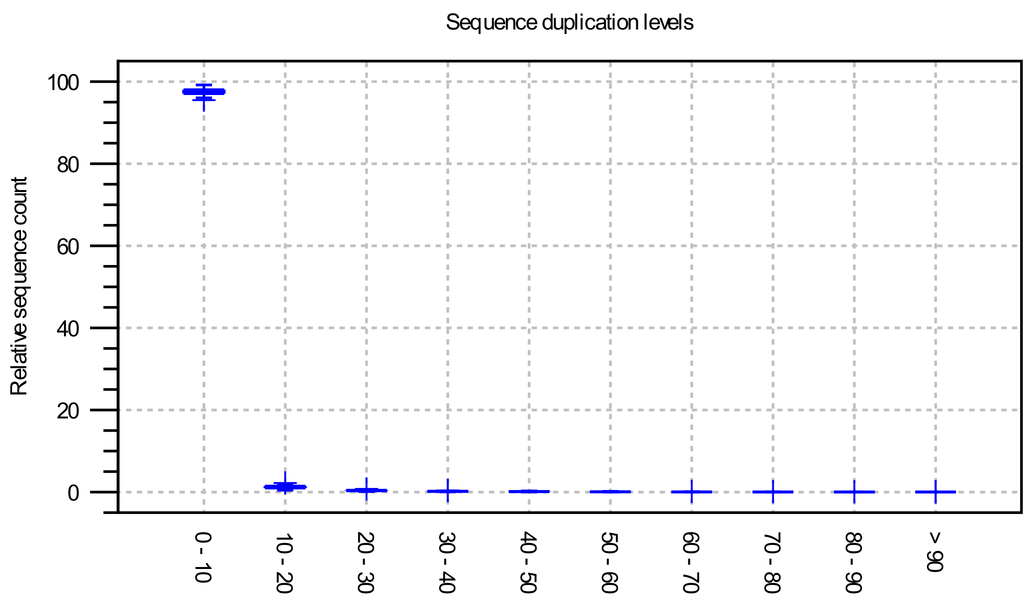
Sequence duplication levels
Relative sequence count
100
80
60
40
20
0
0 - 10
10 - 20
20 - 30
30 - 40
40 - 50
50 - 60
60 - 70
70 - 80
80 - 90
> 90

Table of contents

1. Reads summary 3
    1.1 Summary statistics 3
    1.2 Read length distribution 5
2. Trim reads 8
    2.1 Trim summary 8
    2.2 Read length after trimming 10
    2.3 Detailed trim results 14

1. Reads summary

1.1 Summary statistics

The table is based on 64 samples.

Sample name	Data sets (#)	Reads (#)	Paired reads (%)	Bases (#)
190979_1	1	51.860.276	-	7.779.041.400
190980_S2	1	57.639.418	-	8.645.912.700
190981_S3	1	76.585.178	-	11.487.776.700
190982_S4	1	74.909.674	-	11.236.451.100
190983_S5	1	84.211.104	-	12.631.665.600
190984_S6	1	83.920.234	-	12.588.035.100
190985_S7	1	84.363.200	-	12.654.480.000
190986_S8	1	50.572.368	-	7.585.855.200
190987_S9	1	72.211.908	-	10.831.786.200
190988_S10	1	103.284.056	-	15.492.608.400
190989_S11	1	76.216.542	-	11.432.481.300
190992_S12	1	85.664.610	-	12.849.691.500
190993_S13	1	66.883.100	-	10.032.465.000
190994_S14	1	70.965.070	-	10.644.760.500
190995_S15	1	76.277.292	-	11.441.593.800
190996_S16	1	68.856.782	-	10.328.517.300
190997_S17	1	90.157.680	-	13.523.652.000
190998_S18	1	118.116.462	-	17.717.469.300
190999_S19	1	79.276.516	-	11.891.477.400
191000_S20	1	67.107.218	-	10.066.082.700
191001_S21	1	85.524.584	-	12.828.687.600
191002_S22	1	101.658.880	-	15.248.832.000
191003_S23	1	106.818.522	-	16.022.778.300
191004_S24	1	91.417.592	-	13.712.638.800
191005_S25	1	89.618.970	-	13.442.845.500
191006_S26	1	110.405.300	-	16.560.795.000
191007_S27	1	103.584.542	-	15.537.681.300
191008_S28	1	76.751.694	-	11.512.754.100
191009_S29	1	102.051.848	-	15.307.777.200
191010_S30	1	95.751.286	-	14.362.692.900
191011_S31	1	88.571.064	-	13.285.659.600
191012_S32	1	88.240.996	-	13.236.149.400

Sample name	Data sets (#)	Reads (#)	Paired reads (%)	Bases (#)
191013_S33	1	94.920.068	-	14.238.010.200
191014_S34	1	88.025.888	-	13.203.883.200
191015_S35	1	93.548.336	-	14.032.250.400
191016_S36	1	100.695.730	-	15.104.359.500
191017_S37	1	101.512.444	-	15.226.866.600
191018_S38	1	84.326.668	-	12.649.000.200
191019_S39	1	100.288.534	-	15.043.280.100
191020_S40	1	106.756.214	-	16.013.432.100
191021_S41	1	94.438.202	-	14.165.730.300
191022_S42	1	116.877.504	-	17.531.625.600
191023_S43	1	75.470.924	-	11.320.638.600
191024_S44	1	93.734.350	-	14.060.152.500
191025_S45	1	130.251.246	-	19.537.686.900
191026_S46	1	111.715.376	-	16.757.306.400
191027_S47	1	118.691.480	-	17.803.722.000
191028_S48	1	82.844.912	-	12.426.736.800
191029_S49	1	106.984.422	-	16.047.663.300
191030_S50	1	105.016.498	-	15.752.474.700
191031_S51	1	115.222.302	-	17.283.345.300
191032_S52	1	94.211.386	-	14.131.707.900
191033_S53	1	105.846.956	-	15.877.043.400
191034_S54	1	96.994.300	-	14.549.145.000
191035_S55	1	116.610.434	-	17.491.565.100
191036_S56	1	87.186.660	-	13.077.999.000
191037_S57	1	112.596.746	-	16.889.511.900
191038_S58	1	119.546.242	-	17.931.936.300
191039_S59	1	130.884.680	-	19.632.702.000
191040_S60	1	119.395.784	-	17.909.367.600
191041_S61	1	110.077.048	-	16.511.557.200
191042_S62	1	110.190.516	-	16.528.577.400
191043_S63	1	113.019.250	-	16.952.887.500
191044_S64	1	107.472.130	-	16.120.819.500
Minimum	1,00	50.572.368,00	-	7.585.855.200,00
Median	1,00	94.324.794,00	-	14.148.719.100,00
Maximum	1,00	130.884.680,00	-	19.632.702.000,00
Mean	1,00	94.137.924,94	-	14.120.688.740,62
Standard deviation	0,00	18.224.138,70	-	2.733.620.804,31

1.2 Read length distribution

The table is based on 64 samples.

Sample name	Minimum	25th percentile	Median	75th percentile
190979_1	150	150	150	150
190980_S2	150	150	150	150
190981_S3	150	150	150	150
190982_S4	150	150	150	150
190983_S5	150	150	150	150
190984_S6	150	150	150	150
190985_S7	150	150	150	150
190986_S8	150	150	150	150
190987_S9	150	150	150	150
190988_S10	150	150	150	150
190989_S11	150	150	150	150
190992_S12	150	150	150	150
190993_S13	150	150	150	150
190994_S14	150	150	150	150
190995_S15	150	150	150	150
190996_S16	150	150	150	150
190997_S17	150	150	150	150
190998_S18	150	150	150	150
190999_S19	150	150	150	150
191000_S20	150	150	150	150
191001_S21	150	150	150	150
191002_S22	150	150	150	150
191003_S23	150	150	150	150
191004_S24	150	150	150	150
191005_S25	150	150	150	150
191006_S26	150	150	150	150
191007_S27	150	150	150	150
191008_S28	150	150	150	150
191009_S29	150	150	150	150
191010_S30	150	150	150	150
191011_S31	150	150	150	150
191012_S32	150	150	150	150
191013_S33	150	150	150	150
191014_S34	150	150	150	150
191015_S35	150	150	150	150

Sample name	Minimum	25th percentile	Median	75th percentile
191016_S36	150	150	150	150
191017_S37	150	150	150	150
191018_S38	150	150	150	150
191019_S39	150	150	150	150
191020_S40	150	150	150	150
191021_S41	150	150	150	150
191022_S42	150	150	150	150
191023_S43	150	150	150	150
191024_S44	150	150	150	150
191025_S45	150	150	150	150
191026_S46	150	150	150	150
191027_S47	150	150	150	150
191028_S48	150	150	150	150
191029_S49	150	150	150	150
191030_S50	150	150	150	150
191031_S51	150	150	150	150
191032_S52	150	150	150	150
191033_S53	150	150	150	150
191034_S54	150	150	150	150
191035_S55	150	150	150	150
191036_S56	150	150	150	150
191037_S57	150	150	150	150
191038_S58	150	150	150	150
191039_S59	150	150	150	150
191040_S60	150	150	150	150
191041_S61	150	150	150	150
191042_S62	150	150	150	150
191043_S63	150	150	150	150
191044_S64	150	150	150	150
Minimum	150,00	150,00	150,00	150,00
Median	150,00	150,00	150,00	150,00
Maximum	150,00	150,00	150,00	150,00
Mean	150,00	150,00	150,00	150,00
Standard deviation	0,00	0,00	0,00	0,00

Sample name	Maximum	Mean
190979_1	150	150
190980_S2	150	150
190981_S3	150	150
190982_S4	150	150

Sample name	Maximum	Mean
190983_S5	150	150
190984_S6	150	150
190985_S7	150	150
190986_S8	150	150
190987_S9	150	150
190988_S10	150	150
190989_S11	150	150
190992_S12	150	150
190993_S13	150	150
190994_S14	150	150
190995_S15	150	150
190996_S16	150	150
190997_S17	150	150
190998_S18	150	150
190999_S19	150	150
191000_S20	150	150
191001_S21	150	150
191002_S22	150	150
191003_S23	150	150
191004_S24	150	150
191005_S25	150	150
191006_S26	150	150
191007_S27	150	150
191008_S28	150	150
191009_S29	150	150
191010_S30	150	150
191011_S31	150	150
191012_S32	150	150
191013_S33	150	150
191014_S34	150	150
191015_S35	150	150
191016_S36	150	150
191017_S37	150	150
191018_S38	150	150
191019_S39	150	150
191020_S40	150	150
191021_S41	150	150
191022_S42	150	150
191023_S43	150	150
191024_S44	150	150

Sample name	Maximum	Mean
191025_S45	150	150
191026_S46	150	150
191027_S47	150	150
191028_S48	150	150
191029_S49	150	150
191030_S50	150	150
191031_S51	150	150
191032_S52	150	150
191033_S53	150	150
191034_S54	150	150
191035_S55	150	150
191036_S56	150	150
191037_S57	150	150
191038_S58	150	150
191039_S59	150	150
191040_S60	150	150
191041_S61	150	150
191042_S62	150	150
191043_S63	150	150
191044_S64	150	150
Minimum	150,00	150,00
Median	150,00	150,00
Maximum	150,00	150,00
Mean	150,00	150,00
Standard deviation	0,00	0,00

2. Trim reads

2.1 Trim summary

Summarizes the percentage of reads.

The table is based on 64 samples.

Sample name	Reads after trim (%)	Avg. length after trim
190979_1	91,20	99,11
190980_S2	92,28	104,95
190981_S3	92,32	104,19
190982_S4	92,95	105,26

Sample name	Reads after trim (%)	Avg. length after trim
190983_S5	93,12	104,87
190984_S6	92,96	106,40
190985_S7	92,94	106,98
190986_S8	92,31	99,73
190987_S9	95,09	118,64
190988_S10	95,02	123,19
190989_S11	95,49	124,12
190992_S12	95,04	120,59
190993_S13	95,65	120,65
190994_S14	95,56	121,00
190995_S15	95,52	118,19
190996_S16	95,40	119,40
190997_S17	95,27	116,75
190998_S18	95,31	119,96
190999_S19	94,79	118,46
191000_S20	94,67	116,79
191001_S21	95,35	120,53
191002_S22	95,34	120,81
191003_S23	95,05	121,15
191004_S24	95,37	117,52
191005_S25	94,84	116,20
191006_S26	95,21	117,21
191007_S27	94,77	114,34
191008_S28	93,26	112,04
191009_S29	95,47	118,51
191010_S30	95,40	118,51
191011_S31	94,95	117,56
191012_S32	93,89	113,21
191013_S33	94,93	109,71
191014_S34	94,55	108,65
191015_S35	94,78	111,37
191016_S36	94,39	109,03
191017_S37	95,05	113,82
191018_S38	93,11	109,77
191019_S39	94,88	114,43
191020_S40	94,07	109,96
191021_S41	93,80	106,80
191022_S42	93,72	106,12
191023_S43	94,05	108,39
191024_S44	93,58	107,31

Sample name	Reads after trim (%)	Avg. length after trim
191025_S45	94,23	111,95
191026_S46	93,92	110,90
191027_S47	93,79	109,33
191028_S48	93,46	107,34
191029_S49	93,99	108,99
191030_S50	94,01	109,95
191031_S51	91,89	95,61
191032_S52	93,30	106,47
191033_S53	93,59	105,41
191034_S54	93,44	108,57
191035_S55	93,93	110,53
191036_S56	94,09	110,88
191037_S57	94,97	114,66
191038_S58	95,22	114,05
191039_S59	95,04	113,31
191040_S60	95,41	119,11
191041_S61	95,04	115,89
191042_S62	93,44	105,85
191043_S63	94,22	110,08
191044_S64	94,82	114,78
Minimum	91,20	95,61
Median	94,61	111,99
Maximum	95,65	124,12
Mean	94,29	112,44
Standard deviation	1,04	6,25

	Outliers
Reads after trim (%)	190979_1

2.2 Read length after trimming

Summarizes the percentage of reads.

The table is based on 64 samples.

Sample name	Minimum	25th percentile	Median	75th percentile
190979_1	35	69	98	132
190980_S2	35	74	107	141
190981_S3	35	73	106	140
190982_S4	35	75	107	141
190983_S5	35	75	107	139

Sample name	Minimum	25th percentile	Median	75th percentile
190984_S6	35	76	109	143
190985_S7	35	77	110	143
190986_S8	35	70	98	133
190987_S9	35	96	128	150
190988_S10	35	104	135	150
190989_S11	35	106	137	150
190992_S12	35	99	131	150
190993_S13	35	100	131	150
190994_S14	35	100	131	150
190995_S15	35	95	127	150
190996_S16	35	97	129	150
190997_S17	35	93	125	150
190998_S18	35	98	130	150
190999_S19	35	95	128	150
191000_S20	35	93	126	150
191001_S21	35	100	130	150
191002_S22	35	100	131	150
191003_S23	35	101	131	150
191004_S24	35	95	126	150
191005_S25	35	92	125	150
191006_S26	35	94	125	150
191007_S27	35	90	121	149
191008_S28	35	85	119	148
191009_S29	35	97	127	150
191010_S30	35	96	128	150
191011_S31	35	94	127	150
191012_S32	35	87	121	149
191013_S33	35	85	113	140
191014_S34	35	82	112	141
191015_S35	35	86	116	145
191016_S36	35	83	112	141
191017_S37	35	89	120	147
191018_S38	35	84	114	141
191019_S39	35	90	121	150
191020_S40	35	83	114	145
191021_S41	35	79	109	140
191022_S42	35	78	108	139
191023_S43	35	81	111	142
191024_S44	35	79	110	141
191025_S45	35	85	118	147

Sample name	Minimum	25th percentile	Median	75th percentile
191026_S46	35	84	116	145
191027_S47	35	82	113	143
191028_S48	35	79	110	142
191029_S49	35	82	113	143
191030_S50	35	83	115	145
191031_S51	35	67	93	124
191032_S52	35	77	109	142
191033_S53	35	78	107	138
191034_S54	35	81	112	143
191035_S55	35	84	115	144
191036_S56	35	83	116	147
191037_S57	35	90	122	150
191038_S58	35	89	121	148
191039_S59	35	88	119	147
191040_S60	35	96	129	150
191041_S61	35	92	124	150
191042_S62	35	79	108	137
191043_S63	35	83	114	144
191044_S64	35	90	122	149
Minimum	35,00	67,00	93,00	124,00
Median	35,00	85,50	118,50	147,00
Maximum	35,00	106,00	137,00	150,00
Mean	35,00	86,98	118,23	145,28
Standard deviation	0,00	9,22	9,72	5,43

Sample name	Maximum	Mean
190979_1	150	99,11
190980_S2	150	104,95
190981_S3	150	104,19
190982_S4	150	105,26
190983_S5	150	104,87
190984_S6	150	106,40
190985_S7	150	106,98
190986_S8	150	99,73
190987_S9	150	118,64
190988_S10	150	123,19
190989_S11	150	124,12
190992_S12	150	120,59
190993_S13	150	120,65
190994_S14	150	121,00

Sample name	Maximum	Mean
190995_S15	150	118,19
190996_S16	150	119,40
190997_S17	150	116,75
190998_S18	150	119,96
190999_S19	150	118,46
191000_S20	150	116,79
191001_S21	150	120,53
191002_S22	150	120,81
191003_S23	150	121,15
191004_S24	150	117,52
191005_S25	150	116,20
191006_S26	150	117,21
191007_S27	150	114,34
191008_S28	150	112,04
191009_S29	150	118,51
191010_S30	150	118,51
191011_S31	150	117,56
191012_S32	150	113,21
191013_S33	150	109,71
191014_S34	150	108,65
191015_S35	150	111,37
191016_S36	150	109,03
191017_S37	150	113,82
191018_S38	150	109,77
191019_S39	150	114,43
191020_S40	150	109,96
191021_S41	150	106,80
191022_S42	150	106,12
191023_S43	150	108,39
191024_S44	150	107,31
191025_S45	150	111,95
191026_S46	150	110,90
191027_S47	150	109,33
191028_S48	150	107,34
191029_S49	150	108,99
191030_S50	150	109,95
191031_S51	150	95,61
191032_S52	150	106,47
191033_S53	150	105,41
191034_S54	150	108,57

Sample name	Maximum	Mean
191035_S55	150	110,53
191036_S56	150	110,88
191037_S57	150	114,66
191038_S58	150	114,05
191039_S59	150	113,31
191040_S60	150	119,11
191041_S61	150	115,89
191042_S62	150	105,85
191043_S63	150	110,08
191044_S64	150	114,78
Minimum	150,00	95,61
Median	150,00	111,99
Maximum	150,00	124,12
Mean	150,00	112,44
Standard deviation	0,00	6,25

	Outliers
75th percentile	191031_S51

2.3 Detailed trim results

Trim on quality

Summarizes the percentage of reads.

The table is based on 64 samples.

Sample name	Input (%)	No trim (%)	Trimmed (%)	Nothing left / discarded (%)
190979_1	100,00	36,79	63,21	0,00
190980_S2	100,00	41,86	58,14	0,00
190981_S3	100,00	39,28	60,72	0,00
190982_S4	100,00	40,77	59,23	0,00
190983_S5	100,00	39,81	60,19	0,00
190984_S6	100,00	43,83	56,17	0,00
190985_S7	100,00	43,21	56,79	0,00
190986_S8	100,00	37,09	62,91	0,00
190987_S9	100,00	50,20	49,80	0,00
190988_S10	100,00	52,89	47,11	0,00
190989_S11	100,00	55,53	44,47	0,00
190992_S12	100,00	50,73	49,27	0,00
190993_S13	100,00	54,14	45,86	0,00

Sample name	Input (%)	No trim (%)	Trimmed (%)	Nothing left / discarded (%)
190994_S14	100,00	54,26	45,74	0,00
190995_S15	100,00	53,80	46,20	0,00
190996_S16	100,00	53,43	46,57	0,00
190997_S17	100,00	52,46	47,54	0,00
190998_S18	100,00	53,64	46,36	0,00
190999_S19	100,00	50,30	49,70	0,00
191000_S20	100,00	49,70	50,30	0,00
191001_S21	100,00	52,75	47,25	0,00
191002_S22	100,00	52,55	47,45	0,00
191003_S23	100,00	50,74	49,26	0,00
191004_S24	100,00	51,79	48,21	0,00
191005_S25	100,00	51,08	48,92	0,00
191006_S26	100,00	52,25	47,75	0,00
191007_S27	100,00	49,02	50,98	0,00
191008_S28	100,00	45,50	54,50	0,00
191009_S29	100,00	52,08	47,92	0,00
191010_S30	100,00	52,24	47,76	0,00
191011_S31	100,00	53,32	46,68	0,00
191012_S32	100,00	47,28	52,72	0,00
191013_S33	100,00	45,38	54,62	0,00
191014_S34	100,00	48,06	51,94	0,00
191015_S35	100,00	49,52	50,48	0,00
191016_S36	100,00	40,63	59,37	0,00
191017_S37	100,00	49,27	50,73	0,00
191018_S38	100,00	41,45	58,55	0,00
191019_S39	100,00	50,02	49,98	0,00
191020_S40	100,00	46,90	53,10	0,00
191021_S41	100,00	44,65	55,35	0,00
191022_S42	100,00	45,85	54,15	0,00
191023_S43	100,00	46,48	53,52	0,00
191024_S44	100,00	43,74	56,26	0,00
191025_S45	100,00	46,52	53,48	0,00
191026_S46	100,00	44,34	55,66	0,00
191027_S47	100,00	47,16	52,84	0,00
191028_S48	100,00	43,39	56,61	0,00
191029_S49	100,00	47,59	52,41	0,00
191030_S50	100,00	44,74	55,26	0,00
191031_S51	100,00	38,01	61,99	0,00
191032_S52	100,00	42,42	57,58	0,00

Sample name	Input (%)	No trim (%)	Trimmed (%)	Nothing left / discarded (%)
191033_S53	100,00	42,88	57,12	0,00
191034_S54	100,00	44,60	55,40	0,00
191035_S55	100,00	45,03	54,97	0,00
191036_S56	100,00	46,42	53,58	0,00
191037_S57	100,00	51,04	48,96	0,00
191038_S58	100,00	48,98	51,02	0,00
191039_S59	100,00	46,91	53,09	0,00
191040_S60	100,00	48,45	51,55	0,00
191041_S61	100,00	49,96	50,04	0,00
191042_S62	100,00	41,37	58,63	0,00
191043_S63	100,00	46,21	53,79	0,00
191044_S64	100,00	48,23	51,77	0,00
Minimum	100,00	36,79	44,47	0,00
Median	100,00	47,44	52,56	0,00
Maximum	100,00	55,53	63,21	0,00
Mean	100,00	47,35	52,65	0,00
Standard deviation	0,00	4,69	4,69	0,00

	Outliers
Input (%)	190986_S8, 190989_S11, 191009_S29, 191011_S31, 191013_S33, 191016_S36, 191032_S52
Nothing left / discarded (%)	191008_S28, 191026_S46, 191041_S61

Ambiguity trim

Summarizes the percentage of reads.

The table is based on 64 samples.

Sample name	Input (%)	No trim (%)	Trimmed (%)	Nothing left / discarded (%)
190979_1	100,00	99,93	0,06	0,01
190980_S2	100,00	99,94	0,06	0,01
190981_S3	100,00	99,94	0,06	0,01
190982_S4	100,00	99,94	0,06	0,01
190983_S5	100,00	99,93	0,06	0,01
190984_S6	100,00	99,94	0,06	0,01
190985_S7	100,00	99,93	0,06	0,01
190986_S8	100,00	99,94	0,06	0,01
190987_S9	100,00	99,94	0,06	0,01
190988_S10	100,00	99,94	0,06	0,01
190989_S11	100,00	99,94	0,06	0,01

Sample name	Input (%)	No trim (%)	Trimmed (%)	Nothing left / discarded (%)
190992_S12	100,00	99,94	0,06	0,01
190993_S13	100,00	99,94	0,06	0,01
190994_S14	100,00	99,94	0,06	0,01
190995_S15	100,00	99,93	0,06	0,01
190996_S16	100,00	99,94	0,06	0,01
190997_S17	100,00	99,94	0,06	0,01
190998_S18	100,00	99,94	0,06	0,01
190999_S19	100,00	99,94	0,06	0,01
191000_S20	100,00	99,93	0,06	0,01
191001_S21	100,00	99,94	0,06	0,01
191002_S22	100,00	99,94	0,06	0,01
191003_S23	100,00	99,94	0,06	0,01
191004_S24	100,00	99,94	0,06	0,01
191005_S25	100,00	99,94	0,06	0,01
191006_S26	100,00	99,94	0,06	0,01
191007_S27	100,00	99,94	0,06	0,01
191008_S28	100,00	99,93	0,06	0,01
191009_S29	100,00	99,94	0,06	0,01
191010_S30	100,00	99,94	0,06	0,01
191011_S31	100,00	99,94	0,06	0,01
191012_S32	100,00	99,94	0,06	0,01
191013_S33	100,00	99,94	0,06	0,01
191014_S34	100,00	99,94	0,06	0,01
191015_S35	100,00	99,94	0,06	0,01
191016_S36	100,00	99,94	0,06	0,01
191017_S37	100,00	99,94	0,06	0,01
191018_S38	100,00	99,94	0,06	0,01
191019_S39	100,00	99,94	0,06	0,01
191020_S40	100,00	99,94	0,06	0,01
191021_S41	100,00	99,93	0,06	0,01
191022_S42	100,00	99,94	0,06	0,01
191023_S43	100,00	99,93	0,06	0,01
191024_S44	100,00	99,94	0,06	0,01
191025_S45	100,00	99,94	0,06	0,01
191026_S46	100,00	99,94	0,06	0,01
191027_S47	100,00	99,94	0,06	0,01
191028_S48	100,00	99,94	0,06	0,01
191029_S49	100,00	99,94	0,06	0,01
191030_S50	100,00	99,94	0,06	0,01

Sample name	Input (%)	No trim (%)	Trimmed (%)	Nothing left / discarded (%)
191031_S51	100,00	99,94	0,06	0,01
191032_S52	100,00	99,94	0,06	0,01
191033_S53	100,00	99,94	0,06	0,01
191034_S54	100,00	99,94	0,06	0,01
191035_S55	100,00	99,94	0,06	0,01
191036_S56	100,00	99,94	0,06	0,01
191037_S57	100,00	99,93	0,06	0,01
191038_S58	100,00	99,94	0,06	0,01
191039_S59	100,00	99,94	0,06	0,01
191040_S60	100,00	99,94	0,06	0,01
191041_S61	100,00	99,94	0,06	0,01
191042_S62	100,00	99,94	0,06	0,01
191043_S63	100,00	99,94	0,06	0,01
191044_S64	100,00	99,94	0,06	0,01
Minimum	100,00	99,93	0,06	0,01
Median	100,00	99,94	0,06	0,01
Maximum	100,00	99,94	0,06	0,01
Mean	100,00	99,94	0,06	0,01
Standard deviation	0,00	0,00	0,00	0,00

	Outliers
Input (%)	190986_S8, 190989_S11, 191008_S28, 191009_S29, 191011_S31, 191013_S33, 191016_S36, 191026_S46, 191032_S52, 191041_S61
No trim (%)	191016_S36
Trimmed (%)	191016_S36, 191018_S38
Nothing left / discarded (%)	191016_S36

Filter on length

Summarizes the percentage of reads.

The table is based on 64 samples.

Sample name	Input (%)	No trim (%)	Trimmed (%)	Nothing left / discarded (%)
190979_1	99,99	91,20	0	8,79
190980_S2	99,99	92,28	0	7,72
190981_S3	99,99	92,32	0	7,67
190982_S4	99,99	92,95	0	7,05
190983_S5	99,99	93,12	0	6,88
190984_S6	99,99	92,96	0	7,04

Sample name	Input (%)	No trim (%)	Trimmed (%)	Nothing left / discarded (%)
190985_S7	99,99	92,94	0	7,05
190986_S8	99,99	92,31	0	7,68
190987_S9	99,99	95,09	0	4,91
190988_S10	99,99	95,02	0	4,97
190989_S11	99,99	95,49	0	4,50
190992_S12	99,99	95,04	0	4,95
190993_S13	99,99	95,65	0	4,34
190994_S14	99,99	95,56	0	4,44
190995_S15	99,99	95,52	0	4,47
190996_S16	99,99	95,40	0	4,59
190997_S17	99,99	95,27	0	4,73
190998_S18	99,99	95,31	0	4,68
190999_S19	99,99	94,79	0	5,21
191000_S20	99,99	94,67	0	5,32
191001_S21	99,99	95,35	0	4,64
191002_S22	99,99	95,34	0	4,65
191003_S23	99,99	95,05	0	4,95
191004_S24	99,99	95,37	0	4,63
191005_S25	99,99	94,84	0	5,16
191006_S26	99,99	95,21	0	4,78
191007_S27	99,99	94,77	0	5,22
191008_S28	99,99	93,26	0	6,73
191009_S29	99,99	95,47	0	4,53
191010_S30	99,99	95,40	0	4,60
191011_S31	99,99	94,95	0	5,04
191012_S32	99,99	93,89	0	6,11
191013_S33	99,99	94,93	0	5,07
191014_S34	99,99	94,55	0	5,44
191015_S35	99,99	94,78	0	5,22
191016_S36	99,99	94,39	0	5,61
191017_S37	99,99	95,05	0	4,95
191018_S38	99,99	93,11	0	6,89
191019_S39	99,99	94,88	0	5,12
191020_S40	99,99	94,07	0	5,93
191021_S41	99,99	93,80	0	6,19
191022_S42	99,99	93,72	0	6,27
191023_S43	99,99	94,05	0	5,94
191024_S44	99,99	93,58	0	6,41
191025_S45	99,99	94,23	0	5,77

Sample name	Input (%)	No trim (%)	Trimmed (%)	Nothing left / discarded (%)
191026_S46	99,99	93,92	0	6,07
191027_S47	99,99	93,79	0	6,21
191028_S48	99,99	93,46	0	6,54
191029_S49	99,99	93,99	0	6,01
191030_S50	99,99	94,01	0	5,99
191031_S51	99,99	91,89	0	8,11
191032_S52	99,99	93,30	0	6,69
191033_S53	99,99	93,59	0	6,41
191034_S54	99,99	93,44	0	6,56
191035_S55	99,99	93,93	0	6,06
191036_S56	99,99	94,09	0	5,91
191037_S57	99,99	94,97	0	5,03
191038_S58	99,99	95,22	0	4,77
191039_S59	99,99	95,04	0	4,96
191040_S60	99,99	95,41	0	4,58
191041_S61	99,99	95,04	0	4,95
191042_S62	99,99	93,44	0	6,56
191043_S63	99,99	94,22	0	5,77
191044_S64	99,99	94,82	0	5,18
Minimum	99,99	91,20	0,00	4,34
Median	99,99	94,61	0,00	5,38
Maximum	99,99	95,65	0,00	8,79
Mean	99,99	94,29	0,00	5,71
Standard deviation	0,00	1,04	0,00	1,04

	Outliers
Input (%)	191016_S36
No trim (%)	190979_1
Nothing left / discarded (%)	190979_1

Table of contents

1. Read mapping summary 3
1.1 References 3
1.2 Reads summary 6
1.3 Read length distribution 10
1.4 Mapped reads length distribution 10
1.5 Mapped paired reads 11
1.6 Not mapped reads length distribution 12
2. QC for read mapping 13
2.1 Reference coverage 13
2.2 Non-specific matches 16
2.3 Non-perfect matches 18
2.4 Insertion length distribution 20
2.5 Deletion length distribution 21
2.6 Nucleotide differences in reads relative to reference 21
2.7 Quality for match distribution 22
2.8 Quality for mismatch distribution 22
2.9 Mismatch percentage per read position 23
2.10 Unaligned ends 24

1. Read mapping summary

1.1 References

The table is based on 64 samples.

Sample name	Count	Total length	Average length	Total consensus length
190979_S1	613	2.501.912.388	4.081.423,15	-
190980_S2	613	2.501.912.388	4.081.423,15	-
190981_S3	613	2.501.912.388	4.081.423,15	-
190982_S4	613	2.501.912.388	4.081.423,15	-
190983_S5	613	2.501.912.388	4.081.423,15	-
190984_S6	613	2.501.912.388	4.081.423,15	-
190985_S7	613	2.501.912.388	4.081.423,15	-
190986_S8	613	2.501.912.388	4.081.423,15	-
190987_S9	613	2.501.912.388	4.081.423,15	-
190988_S10	613	2.501.912.388	4.081.423,15	-
190989_S11	613	2.501.912.388	4.081.423,15	-
190992_S12	613	2.501.912.388	4.081.423,15	-
190993_S13	613	2.501.912.388	4.081.423,15	-
190994_S14	613	2.501.912.388	4.081.423,15	-
190995_S15	613	2.501.912.388	4.081.423,15	-
190996_S16	613	2.501.912.388	4.081.423,15	-
190997_S17	613	2.501.912.388	4.081.423,15	-
190998_S18	613	2.501.912.388	4.081.423,15	-
190999_S19	613	2.501.912.388	4.081.423,15	-
191000_S20	613	2.501.912.388	4.081.423,15	-
191001_S21	613	2.501.912.388	4.081.423,15	-
191002_S22	613	2.501.912.388	4.081.423,15	-
191003_S23	613	2.501.912.388	4.081.423,15	-
191004_S24	613	2.501.912.388	4.081.423,15	-
191005_S25	613	2.501.912.388	4.081.423,15	-
191006_S26	613	2.501.912.388	4.081.423,15	-
191007_S27	613	2.501.912.388	4.081.423,15	-
191008_S28	613	2.501.912.388	4.081.423,15	-
191009_S29	613	2.501.912.388	4.081.423,15	-
191010_S30	613	2.501.912.388	4.081.423,15	-
191011_S31	613	2.501.912.388	4.081.423,15	-
191012_S32	613	2.501.912.388	4.081.423,15	-

Sample name	Count	Total length	Average length	Total consensus length
191013_S33	613	2.501.912.388	4.081.423,15	-
191014_S34	613	2.501.912.388	4.081.423,15	-
191015_S35	613	2.501.912.388	4.081.423,15	-
191016_S36	613	2.501.912.388	4.081.423,15	-
191017_S37	613	2.501.912.388	4.081.423,15	-
191018_S38	613	2.501.912.388	4.081.423,15	-
191019_S39	613	2.501.912.388	4.081.423,15	-
191020_S40	613	2.501.912.388	4.081.423,15	-
191021_S41	613	2.501.912.388	4.081.423,15	-
191022_S42	613	2.501.912.388	4.081.423,15	-
191023_S43	613	2.501.912.388	4.081.423,15	-
191024_S44	613	2.501.912.388	4.081.423,15	-
191025_S45	613	2.501.912.388	4.081.423,15	-
191026_S46	613	2.501.912.388	4.081.423,15	-
191027_S47	613	2.501.912.388	4.081.423,15	-
191028_S48	613	2.501.912.388	4.081.423,15	-
191029_S49	613	2.501.912.388	4.081.423,15	-
191030_S50	613	2.501.912.388	4.081.423,15	-
191031_S51	613	2.501.912.388	4.081.423,15	-
191032_S52	613	2.501.912.388	4.081.423,15	-
191033_S53	613	2.501.912.388	4.081.423,15	-
191034_S54	613	2.501.912.388	4.081.423,15	-
191035_S55	613	2.501.912.388	4.081.423,15	-
191036_S56	613	2.501.912.388	4.081.423,15	-
191037_S57	613	2.501.912.388	4.081.423,15	-
191037_S58	613	2.501.912.388	4.081.423,15	-
191039_S59	613	2.501.912.388	4.081.423,15	-
191040_S60	613	2.501.912.388	4.081.423,15	-
191041_S61	613	2.501.912.388	4.081.423,15	-
191042_S62	613	2.501.912.388	4.081.423,15	-
191043_S63	613	2.501.912.388	4.081.423,15	-
191044_S64	613	2.501.912.388	4.081.423,15	-
Minimum	613,00	2.501.912.388,00	4.081.423,15	-
Median	613,00	2.501.912.388,00	4.081.423,15	-
Maximum	613,00	2.501.912.388,00	4.081.423,15	-
Mean	613,00	2.501.912.388,00	4.081.423,15	-
Standard deviation	0,00	0,00	0,00	-

Sample name	GC (%)
190979_S1	41,37

Sample name	GC (%)
190980_S2	41,37
190981_S3	41,37
190982_S4	41,37
190983_S5	41,37
190984_S6	41,37
190985_S7	41,37
190986_S8	41,37
190987_S9	41,37
190988_S10	41,37
190989_S11	41,37
190992_S12	41,37
190993_S13	41,37
190994_S14	41,37
190995_S15	41,37
190996_S16	41,37
190997_S17	41,37
190998_S18	41,37
190999_S19	41,37
191000_S20	41,37
191001_S21	41,37
191002_S22	41,37
191003_S23	41,37
191004_S24	41,37
191005_S25	41,37
191006_S26	41,37
191007_S27	41,37
191008_S28	41,37
191009_S29	41,37
191010_S30	41,37
191011_S31	41,37
191012_S32	41,37
191013_S33	41,37
191014_S34	41,37
191015_S35	41,37
191016_S36	41,37
191017_S37	41,37
191018_S38	41,37
191019_S39	41,37
191020_S40	41,37
191021_S41	41,37

Sample name	GC (%)
191022_S42	41,37
191023_S43	41,37
191024_S44	41,37
191025_S45	41,37
191026_S46	41,37
191027_S47	41,37
191028_S48	41,37
191029_S49	41,37
191030_S50	41,37
191031_S51	41,37
191032_S52	41,37
191033_S53	41,37
191034_S54	41,37
191035_S55	41,37
191036_S56	41,37
191037_S57	41,37
191037_S58	41,37
191039_S59	41,37
191040_S60	41,37
191041_S61	41,37
191042_S62	41,37
191043_S63	41,37
191044_S64	41,37
Minimum	41,37
Median	41,37
Maximum	41,37
Mean	41,37
Standard deviation	0,00

1.2 Reads summary

The table is based on 64 samples.

Sample name	Reads (#)	Mapped reads (#)	Mapped reads (%)	Not mapped reads (#)
190979_S1	-	42.916.621	-	-
190980_S2	-	48.611.046	-	-
190981_S3	-	64.241.436	-	-
190982_S4	-	63.463.737	-	-
190983_S5	-	71.639.587	-	-
190984_S6	-	71.563.708	-	-

Sample name	Reads (#)	Mapped reads (#)	Mapped reads (%)	Not mapped reads (#)
190985_S7	-	71.772.952	-	-
190986_S8	-	42.534.151	-	-
190987_S9	-	63.159.551	-	-
190988_S10	-	91.026.813	-	-
190989_S11	-	68.064.704	-	-
190992_S12	-	75.772.213	-	-
190993_S13	-	59.732.429	-	-
190994_S14	-	63.197.731	-	-
190995_S15	-	68.222.625	-	-
190996_S16	-	61.362.817	-	-
190997_S17	-	80.033.478	-	-
190998_S18	-	104.632.218	-	-
190999_S19	-	69.188.896	-	-
191000_S20	-	58.064.077	-	-
191001_S21	-	76.147.029	-	-
191002_S22	-	90.343.233	-	-
191003_S23	-	94.168.888	-	-
191004_S24	-	80.841.650	-	-
191005_S25	-	78.518.373	-	-
191006_S26	-	97.642.330	-	-
191007_S27	-	90.968.496	-	-
191008_S28	-	65.634.623	-	-
191009_S29	-	90.615.698	-	-
191010_S30	-	85.036.932	-	-
191011_S31	-	78.216.935	-	-
191012_S32	-	76.435.632	-	-
191013_S33	-	83.237.516	-	-
191014_S34	-	76.682.300	-	-
191015_S35	-	81.770.841	-	-
191016_S36	-	87.607.860	-	-
191017_S37	-	89.145.742	-	-
191018_S38	-	71.691.777	-	-
191019_S39	-	87.877.137	-	-
191020_S40	-	92.399.872	-	-
191021_S41	-	81.320.749	-	-
191022_S42	-	100.453.317	-	-
191023_S43	-	65.411.364	-	-
191024_S44	-	80.686.438	-	-
191025_S45	-	113.515.926	-	-
191026_S46	-	96.686.858	-	-

Sample name	Reads (#)	Mapped reads (#)	Mapped reads (%)	Not mapped reads (#)
191027_S47	-	102.631.058	-	-
191028_S48	-	71.185.880	-	-
191029_S49	-	92.606.826	-	-
191030_S50	-	91.386.304	-	-
191031_S51	-	96.345.410	-	-
191032_S52	-	80.658.502	-	-
191033_S53	-	91.074.520	-	-
191034_S54	-	83.631.252	-	-
191035_S55	-	101.644.908	-	-
191036_S56	-	75.707.943	-	-
191037_S57	-	99.414.861	-	-
191037_S58	-	105.839.682	-	-
191039_S59	-	115.639.715	-	-
191040_S60	-	106.154.104	-	-
191041_S61	-	97.684.193	-	-
191042_S62	-	94.763.335	-	-
191043_S63	-	98.479.467	-	-
191044_S64	-	94.490.086	-	-
Minimum	-	42.534.151,00	-	-
Median	-	81.545.795,00	-	-
Maximum	-	115.639.715,00	-	-
Mean	-	82.056.193,00	-	-
Standard deviation	-	16.245.550,12	-	-

Sample name	Not mapped reads (%)
190979_S1	-
190980_S2	-
190981_S3	-
190982_S4	-
190983_S5	-
190984_S6	-
190985_S7	-
190986_S8	-
190987_S9	-
190988_S10	-
190989_S11	-
190992_S12	-
190993_S13	-
190994_S14	-
190995_S15	-

Sample name	Not mapped reads (%)
190996_S16	-
190997_S17	-
190998_S18	-
190999_S19	-
191000_S20	-
191001_S21	-
191002_S22	-
191003_S23	-
191004_S24	-
191005_S25	-
191006_S26	-
191007_S27	-
191008_S28	-
191009_S29	-
191010_S30	-
191011_S31	-
191012_S32	-
191013_S33	-
191014_S34	-
191015_S35	-
191016_S36	-
191017_S37	-
191018_S38	-
191019_S39	-
191020_S40	-
191021_S41	-
191022_S42	-
191023_S43	-
191024_S44	-
191025_S45	-
191026_S46	-
191027_S47	-
191028_S48	-
191029_S49	-
191030_S50	-
191031_S51	-
191032_S52	-
191033_S53	-
191034_S54	-
191035_S55	-

Sample name	Not mapped reads (%)
191036_S56	-
191037_S57	-
191037_S58	-
191039_S59	-
191040_S60	-
191041_S61	-
191042_S62	-
191043_S63	-
191044_S64	-
Minimum	-
Median	-
Maximum	-
Mean	-
Standard deviation	-

1.3 Read length distribution

No data available

1.4 Mapped reads length distribution

The box plot is based on 64 samples.

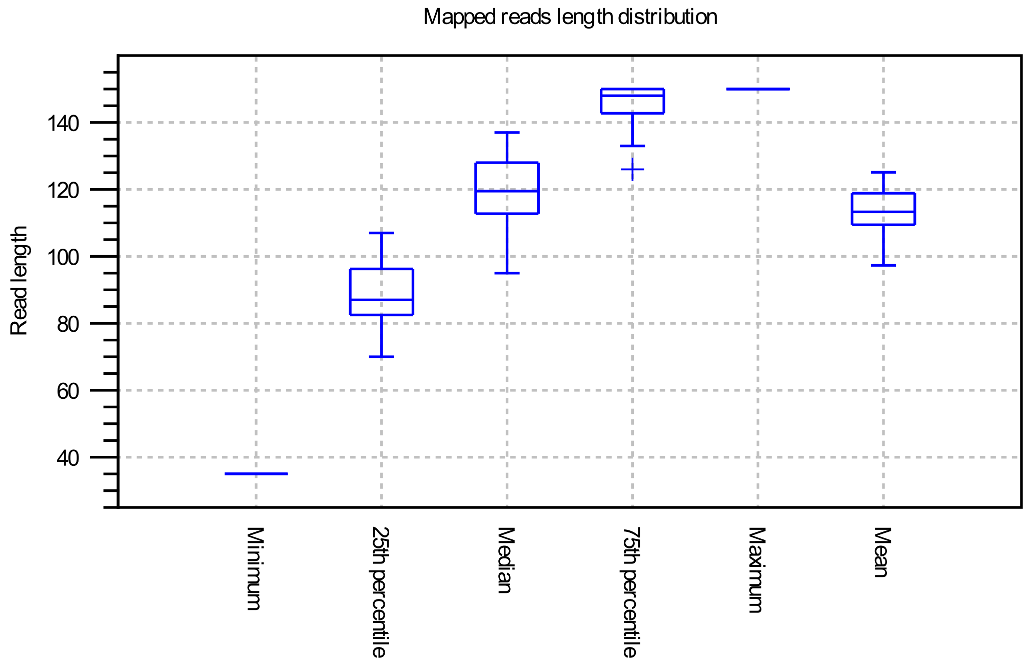

1.5 Mapped paired reads

The table is based on 64 samples.

Sample name	Paired reads (%)	Broken reads (%)	Mean distance	Standard deviation
190979_S1	98,84	1,16	1.084,83	5.315,88
190980_S2	98,61	1,39	1.183,78	5.573,32
190981_S3	98,48	1,52	1.126,44	5.287,38
190982_S4	98,39	1,61	1.180,28	5.521,88
190983_S5	98,58	1,42	1.211,85	5.587,71
190984_S6	98,57	1,43	1.313,33	5.818,52
190985_S7	98,57	1,43	1.184,25	5.359,17
190986_S8	98,93	1,07	1.137,30	5.315,24
190987_S9	97,58	2,42	1.387,65	6.204,74
190988_S10	97,35	2,65	1.490,04	6.450,54
190989_S11	97,79	2,21	1.653,77	6.685,86
190992_S12	98,02	1,98	1.444,56	6.185,95
190993_S13	98,08	1,92	1.547,66	6.435,43
190994_S14	97,95	2,05	1.466,36	6.173,27
190995_S15	98,55	1,45	1.530,00	6.364,87
190996_S16	98,25	1,75	1.459,86	6.226,26
190997_S17	98,40	1,60	1.413,84	6.072,69
190998_S18	97,86	2,14	1.574,51	6.315,71
190999_S19	97,54	2,46	1.633,34	6.490,11
191000_S20	97,47	2,53	1.562,12	6.351,89
191001_S21	98,02	1,98	1.591,10	6.565,90
191002_S22	98,06	1,94	1.529,62	6.428,42
191003_S23	97,72	2,28	1.552,54	6.467,27
191004_S24	98,14	1,86	1.426,28	6.185,36
191005_S25	97,93	2,07	1.224,49	5.594,36
191006_S26	98,31	1,69	1.338,59	5.834,14
191007_S27	98,29	1,71	1.328,80	5.868,13
191008_S28	98,09	1,91	1.208,38	5.646,69
191009_S29	98,08	1,92	1.272,12	5.858,78
191010_S30	98,06	1,94	1.383,98	6.189,34
191011_S31	98,38	1,62	1.316,30	5.881,48
191012_S32	98,21	1,79	1.245,16	5.752,98
191013_S33	98,71	1,29	1.246,87	5.525,55
191014_S34	98,69	1,31	1.304,43	5.689,92
191015_S35	98,41	1,59	1.427,66	5.982,12

Sample name	Paired reads (%)	Broken reads (%)	Mean distance	Standard deviation
191016_S36	98,66	1,34	1.211,66	5.424,73
191017_S37	98,33	1,67	1.217,44	5.510,31
191018_S38	98,38	1,62	1.359,46	5.884,08
191019_S39	98,15	1,85	1.336,45	5.776,43
191020_S40	98,25	1,75	1.314,23	5.819,73
191021_S41	98,55	1,45	1.247,35	5.517,24
191022_S42	98,60	1,40	1.296,53	5.794,26
191023_S43	98,46	1,54	1.298,72	5.717,04
191024_S44	98,52	1,48	1.319,14	5.819,38
191025_S45	98,29	1,71	1.212,68	5.576,31
191026_S46	98,40	1,60	1.191,39	5.515,09
191027_S47	98,62	1,38	1.184,09	5.554,16
191028_S48	98,52	1,48	1.124,40	5.419,61
191029_S49	98,62	1,38	1.213,17	5.387,04
191030_S50	98,60	1,40	1.369,09	5.998,49
191031_S51	99,19	0,81	1.034,38	5.030,39
191032_S52	98,47	1,53	1.333,73	5.896,69
191033_S53	98,80	1,20	1.193,91	5.408,72
191034_S54	98,68	1,32	1.249,14	5.673,00
191035_S55	98,73	1,27	1.309,38	5.555,15
191036_S56	98,48	1,52	1.285,68	5.661,94
191037_S57	98,51	1,49	1.458,56	6.112,54
191037_S58	98,57	1,43	1.357,41	5.960,78
191039_S59	98,63	1,37	1.301,63	5.808,25
191040_S60	98,11	1,89	1.534,08	6.445,45
191041_S61	98,48	1,52	1.444,83	6.135,39
191042_S62	98,76	1,24	1.204,70	5.514,45
191043_S63	98,51	1,49	1.391,22	5.867,85
191044_S64	98,33	1,67	1.392,86	6.064,18
Minimum	97,35	0,81	1.034,38	5.030,39
Median	98,40	1,60	1.315,27	5.819,56
Maximum	99,19	2,65	1.653,77	6.685,86
Mean	98,35	1,65	1.333,90	5.860,24
Standard deviation	0,36	0,36	142,69	378,18

1.6 Not mapped reads length distribution

No data available

2. QC for read mapping

2.1 Reference coverage

The table is based on 64 samples.

Sample name	Covered (%)	Minimum	Median	Mean
190979_S1	4,49	0	0	1,54
190980_S2	4,80	0	0	1,86
190981_S3	5,98	0	0	2,43
190982_S4	6,15	0	0	2,43
190983_S5	5,16	0	0	2,72
190984_S6	5,09	0	0	2,77
190985_S7	5,75	0	0	2,80
190986_S8	3,86	0	0	1,53
190987_S9	5,95	0	0	2,76
190988_S10	6,49	0	0	4,17
190989_S11	5,11	0	0	3,14
190992_S12	5,84	0	0	3,38
190993_S13	5,07	0	0	2,66
190994_S14	5,02	0	0	2,82
190995_S15	3,80	0	0	2,96
190996_S16	4,56	0	0	2,70
190997_S17	5,72	0	0	3,43
190998_S18	5,87	0	0	4,62
190999_S19	4,88	0	0	3,02
191000_S20	4,01	0	0	2,49
191001_S21	5,65	0	0	3,39
191002_S22	5,98	0	0	4,03
191003_S23	6,52	0	0	4,22
191004_S24	5,96	0	0	3,49
191005_S25	5,59	0	0	3,35
191006_S26	6,18	0	0	4,20
191007_S27	5,90	0	0	3,81
191008_S28	6,21	0	0	2,70
191009_S29	5,95	0	0	3,95
191010_S30	6,09	0	0	3,71
191011_S31	6,29	0	0	3,38
191012_S32	5,99	0	0	3,17

Sample name	Covered (%)	Minimum	Median	Mean
191013_S33	4,15	0	0	3,30
191014_S34	5,03	0	0	3,02
191015_S35	5,28	0	0	3,31
191016_S36	5,26	0	0	3,46
191017_S37	6,88	0	0	3,70
191018_S38	4,95	0	0	2,88
191019_S39	6,34	0	0	3,67
191020_S40	5,55	0	0	3,70
191021_S41	4,95	0	0	3,15
191022_S42	5,56	0	0	3,85
191023_S43	4,34	0	0	2,57
191024_S44	4,98	0	0	3,14
191025_S45	6,37	0	0	4,64
191026_S46	6,11	0	0	3,92
191027_S47	5,96	0	0	4,08
191028_S48	5,25	0	0	2,78
191029_S49	6,35	0	0	3,67
191030_S50	5,32	0	0	3,66
191031_S51	5,60	0	0	3,28
191032_S52	6,00	0	0	3,12
191033_S53	5,27	0	0	3,47
191034_S54	5,32	0	0	3,31
191035_S55	5,43	0	0	4,10
191036_S56	5,94	0	0	3,06
191037_S57	4,90	0	0	4,16
191037_S58	5,64	0	0	4,40
191039_S59	5,65	0	0	4,78
191040_S60	5,27	0	0	4,66
191041_S61	5,00	0	0	4,15
191042_S62	4,96	0	0	3,63
191043_S63	4,77	0	0	3,94
191044_S64	5,39	0	0	3,97
Minimum	3,80	0,00	0,00	1,53
Median	5,55	0,00	0,00	3,38
Maximum	6,88	0,00	0,00	4,78
Mean	5,46	0,00	0,00	3,38
Standard deviation	0,68	0,00	0,00	0,71

Sample name	Standard deviation	Maximum
190979_S1	113,88	134.860

Sample name	Standard deviation	Maximum
190980_S2	141,70	175.471
190981_S3	155,98	183.815
190982_S4	157,39	180.213
190983_S5	176,59	188.409
190984_S6	175,86	196.728
190985_S7	171,79	165.633
190986_S8	111,81	142.937
190987_S9	198,68	204.063
190988_S10	346,38	434.632
190989_S11	239,22	240.879
190992_S12	265,48	260.141
190993_S13	200,99	183.403
190994_S14	239,69	264.420
190995_S15	237,54	249.223
190996_S16	332,93	497.962
190997_S17	357,03	580.543
190998_S18	342,47	373.368
190999_S19	192,22	227.214
191000_S20	182,42	232.451
191001_S21	216,37	236.054
191002_S22	295,20	294.357
191003_S23	267,68	302.112
191004_S24	231,91	260.457
191005_S25	509,96	821.403
191006_S26	424,65	589.646
191007_S27	344,68	449.522
191008_S28	250,81	319.559
191009_S29	370,33	406.089
191010_S30	283,24	319.200
191011_S31	250,84	246.862
191012_S32	283,28	342.520
191013_S33	378,48	634.741
191014_S34	214,69	263.655
191015_S35	204,86	204.808
191016_S36	335,90	568.405
191017_S37	284,06	361.401
191018_S38	188,61	174.722
191019_S39	218,34	173.772
191020_S40	264,28	237.691
191021_S41	253,72	325.500

Sample name	Standard deviation	Maximum
191022_S42	258,06	280.166
191023_S43	230,20	288.282
191024_S44	225,71	237.603
191025_S45	381,47	380.693
191026_S46	361,26	434.543
191027_S47	360,89	455.694
191028_S48	325,47	477.602
191029_S49	265,66	379.062
191030_S50	257,81	367.281
191031_S51	256,79	437.184
191032_S52	181,45	179.689
191033_S53	238,64	273.862
191034_S54	266,51	319.415
191035_S55	294,49	406.845
191036_S56	177,06	151.643
191037_S57	259,19	286.605
191037_S58	288,64	271.858
191039_S59	519,91	970.186
191040_S60	295,37	288.297
191041_S61	369,10	488.142
191042_S62	286,09	278.335
191043_S63	272,92	267.488
191044_S64	281,20	255.300
Minimum	111,81	134.860,00
Median	258,63	279.250,50
Maximum	519,91	970.186,00
Mean	266,65	325.384,62
Standard deviation	82,76	157.642,34

2.2 Non-specific matches

The table is based on 64 samples.

Sample name	Reads (%)	Mean read length
190979_S1	5,59	87,75
190980_S2	4,98	92,33
190981_S3	4,09	90,08
190982_S4	4,07	91,13
190983_S5	5,87	95,58
190984_S6	5,08	95,88

Sample name	Reads (%)	Mean read length
190985_S7	5,95	97,48
190986_S8	9,08	92,69
190987_S9	3,38	106,50
190988_S10	3,13	112,49
190989_S11	6,16	117,31
190992_S12	6,66	113,29
190993_S13	5,77	112,62
190994_S14	7,80	114,46
190995_S15	6,29	110,21
190996_S16	13,32	114,34
190997_S17	12,02	111,93
190998_S18	7,86	114,14
190999_S19	4,73	109,38
191000_S20	4,21	106,82
191001_S21	4,30	113,24
191002_S22	6,84	115,05
191003_S23	4,13	113,62
191004_S24	4,80	110,27
191005_S25	16,14	110,46
191006_S26	9,66	111,00
191007_S27	5,71	104,74
191008_S28	7,20	102,67
191009_S29	6,99	111,22
191010_S30	3,90	108,43
191011_S31	4,50	106,97
191012_S32	5,24	102,11
191013_S33	13,47	103,97
191014_S34	7,81	101,45
191015_S35	5,27	102,25
191016_S36	11,70	103,10
191017_S37	8,16	107,28
191018_S38	5,71	102,98
191019_S39	5,49	106,04
191020_S40	7,03	101,27
191021_S41	5,05	95,23
191022_S42	4,71	94,85
191023_S43	4,99	96,12
191024_S44	4,77	95,09
191025_S45	5,28	100,59
191026_S46	6,93	102,17

Sample name	Reads (%)	Mean read length
191027_S47	9,15	101,51
191028_S48	13,31	100,38
191029_S49	8,57	102,13
191030_S50	8,14	102,92
191031_S51	9,89	88,90
191032_S52	4,66	94,82
191033_S53	7,56	98,06
191034_S54	8,27	100,93
191035_S55	7,74	103,33
191036_S56	5,53	101,65
191037_S57	6,22	106,88
191037_S58	4,72	104,44
191039_S59	11,31	108,64
191040_S60	5,53	111,00
191041_S61	9,04	109,68
191042_S62	5,80	95,87
191043_S63	5,60	100,44
191044_S64	4,94	105,15
Minimum	3,13	87,75
Median	5,84	103,22
Maximum	16,14	117,31
Mean	6,84	103,83
Standard deviation	2,74	7,33

2.3 Non-perfect matches

The table is based on 64 samples.

Sample name	Reads (%)	Mean read length
190979_S1	97,97	101,32
190980_S2	97,80	107,08
190981_S3	97,65	106,37
190982_S4	97,60	107,28
190983_S5	97,63	107,00
190984_S6	97,64	108,48
190985_S7	97,07	109,08
190986_S8	98,00	101,88
190987_S9	96,42	120,62
190988_S10	95,38	125,52
190989_S11	95,88	126,17

Sample name	Reads (%)	Mean read length
190992_S12	95,98	122,67
190993_S13	96,24	122,52
190994_S14	96,63	122,81
190995_S15	96,84	119,91
190996_S16	96,33	121,31
190997_S17	96,65	118,54
190998_S18	96,64	121,74
190999_S19	96,10	120,40
191000_S20	96,48	118,67
191001_S21	96,18	122,50
191002_S22	96,25	122,78
191003_S23	95,83	123,30
191004_S24	96,69	119,29
191005_S25	96,99	118,03
191006_S26	97,11	118,88
191007_S27	97,25	116,05
191008_S28	96,85	114,06
191009_S29	96,71	120,34
191010_S30	96,61	120,43
191011_S31	96,95	119,30
191012_S32	97,00	115,10
191013_S33	97,66	111,24
191014_S34	97,76	110,25
191015_S35	97,44	112,97
191016_S36	97,87	110,54
191017_S37	97,13	115,49
191018_S38	95,00	112,46
191019_S39	97,22	116,12
191020_S40	97,47	111,64
191021_S41	97,64	108,44
191022_S42	97,72	107,76
191023_S43	97,58	110,00
191024_S44	97,64	108,98
191025_S45	97,19	113,70
191026_S46	96,85	112,81
191027_S47	97,31	111,11
191028_S48	97,49	109,14
191029_S49	97,65	110,71
191030_S50	97,39	111,79
191031_S51	98,76	97,38

Sample name	Reads (%)	Mean read length
191032_S52	97,42	108,33
191033_S53	97,92	107,19
191034_S54	97,25	110,41
191035_S55	97,33	112,29
191036_S56	97,43	112,76
191037_S57	97,39	116,38
191037_S58	97,27	115,79
191039_S59	97,22	115,16
191040_S60	96,64	121,14
191041_S61	96,61	117,73
191042_S62	97,52	107,66
191043_S63	97,48	111,82
191044_S64	97,04	116,55
Minimum	95,00	97,38
Median	97,22	113,88
Maximum	98,76	126,17
Mean	97,07	114,30
Standard deviation	0,68	6,26

2.4 Insertion length distribution

The box plot is based on 64 samples.

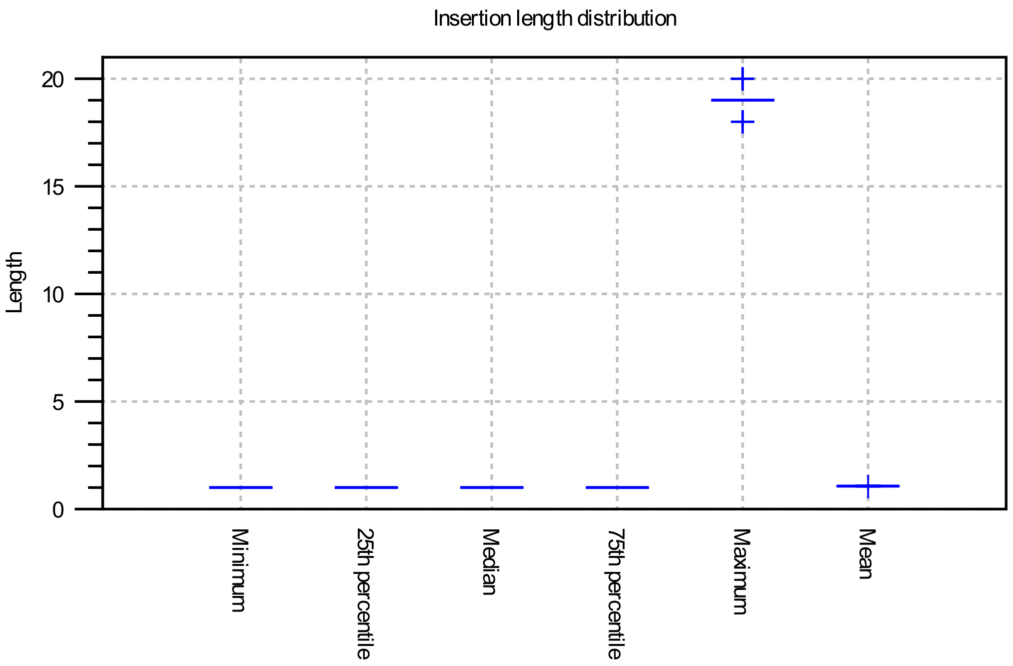

2.5 Deletion length distribution

The box plot is based on 64 samples.

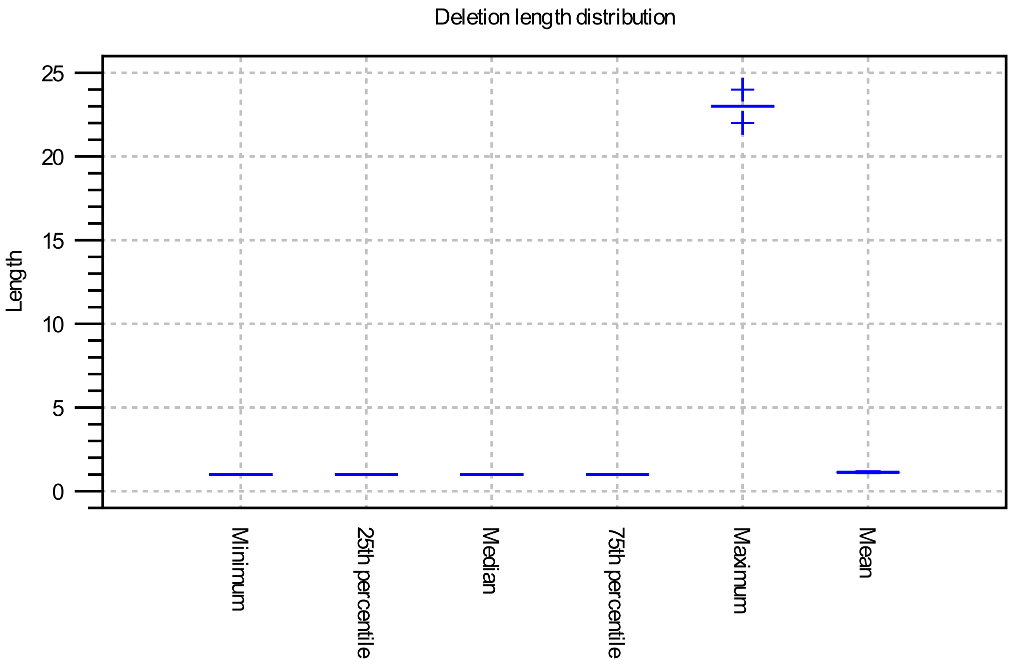

2.6 Nucleotide differences in reads relative to reference

Summarizes the percentage of read bases that differ relative to the nucleotide in reference.

The box plot is based on 64 samples.

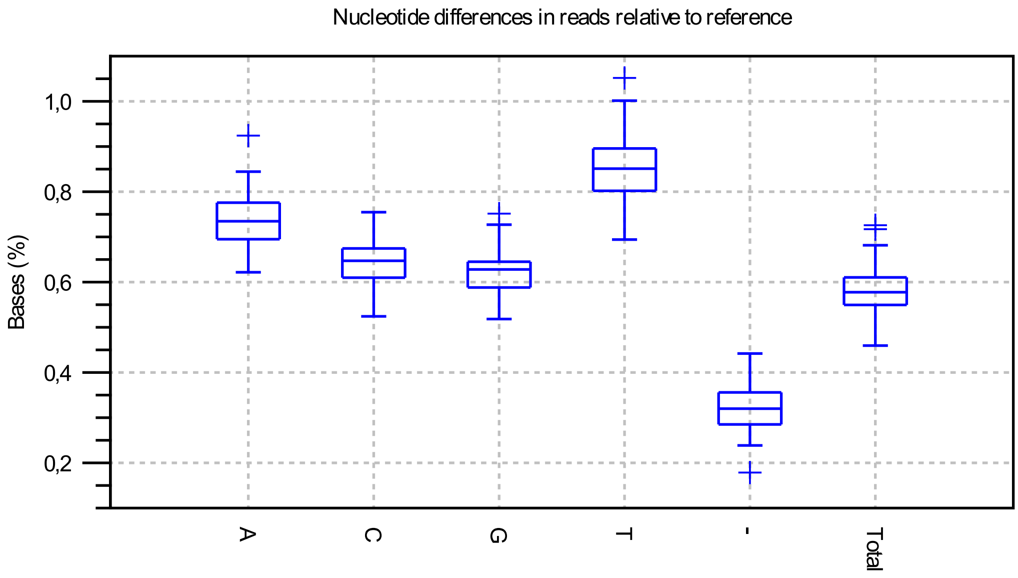

2.7 Quality for match distribution

The box plot is based on 64 samples.

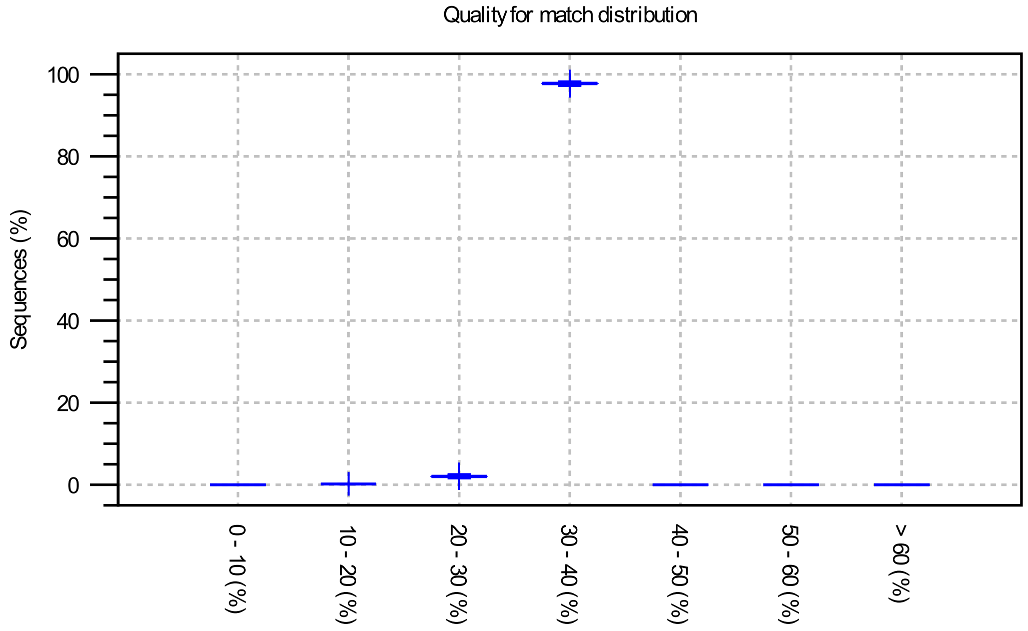

2.8 Quality for mismatch distribution

Summarizes the mean mismatch percentage observed at the base positions in the sequenced segments.

The box plot is based on 64 samples.

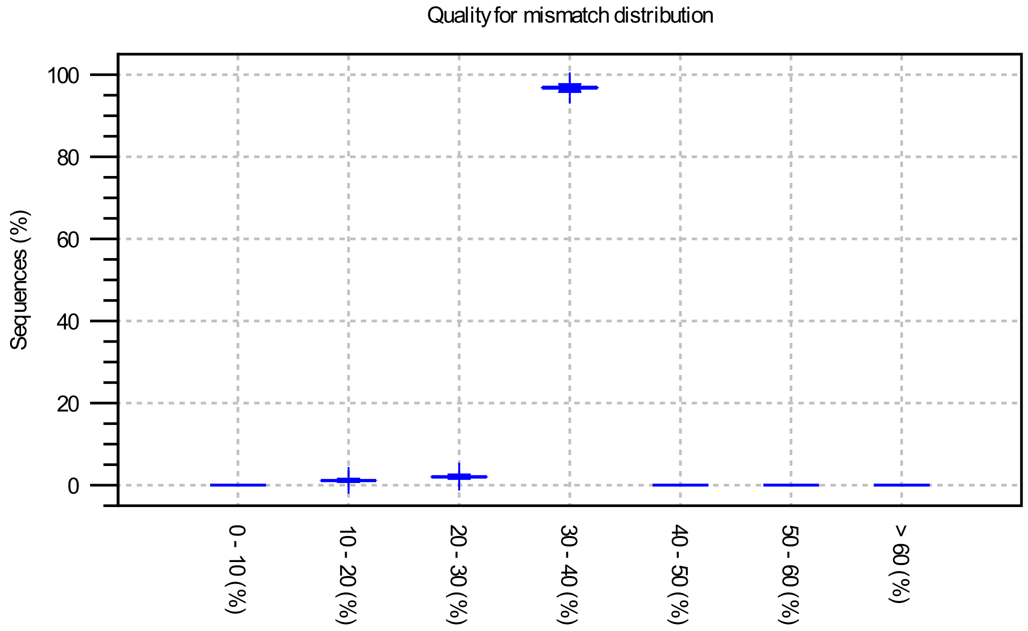

2.9 Mismatch percentage per read position

Summarizes the mean mismatch percentage observed at the base positions in the sequenced segments.

The box plot is based on 64 samples.

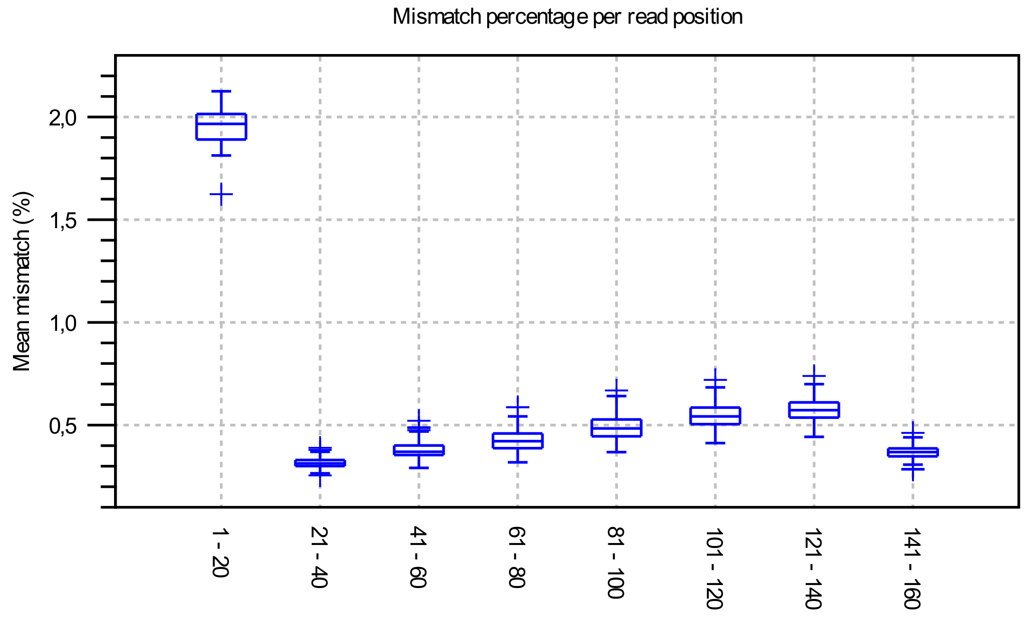

2.10 Unaligned ends

The table is based on 64 samples.

Sample name	Reads (%)	Positions covered in reference (%)	Positions covered in bases covered (%)
190979_S1	143,77	0,39	8,68
190980_S2	139,47	0,40	8,39
190981_S3	139,72	0,50	8,43
190982_S4	138,64	0,53	8,60
190983_S5	141,45	0,50	9,63
190984_S6	138,79	0,48	9,49
190985_S7	137,01	0,46	8,09
190986_S8	145,65	0,36	9,39
190987_S9	125,57	0,45	7,61
190988_S10	115,60	0,51	7,84
190989_S11	117,33	0,40	7,84
190992_S12	121,21	0,44	7,51
190993_S13	123,86	0,40	7,96
190994_S14	124,23	0,41	8,08
190995_S15	129,06	0,35	9,23
190996_S16	125,75	0,38	8,29
190997_S17	129,18	0,49	8,65
190998_S18	124,99	0,54	9,19
190999_S19	123,03	0,40	8,13
191000_S20	126,65	0,32	7,87
191001_S21	123,65	0,51	9,07
191002_S22	123,39	0,51	8,55
191003_S23	120,88	0,54	8,36
191004_S24	128,47	0,51	8,62
191005_S25	128,85	0,44	7,84
191006_S26	129,61	0,52	8,43
191007_S27	131,45	0,54	9,10
191008_S28	129,49	0,50	8,02
191009_S29	127,85	0,50	8,34
191010_S30	126,32	0,54	8,84
191011_S31	128,10	0,48	7,69
191012_S32	129,95	0,51	8,58
191013_S33	140,84	0,44	10,62
191014_S34	139,45	0,50	10,02
191015_S35	136,37	0,53	10,05

Sample name	Reads (%)	Positions covered in reference (%)	Positions covered in bases covered (%)
191016_S36	139,56	0,52	9,79
191017_S37	133,61	0,55	7,93
191018_S38	128,80	0,45	9,16
191019_S39	132,99	0,57	8,93
191020_S40	135,16	0,55	9,84
191021_S41	138,26	0,50	10,06
191022_S42	139,40	0,59	10,58
191023_S43	136,71	0,43	10,00
191024_S44	136,94	0,53	10,57
191025_S45	132,67	0,57	8,99
191026_S46	132,12	0,54	8,80
191027_S47	135,86	0,55	9,27
191028_S48	136,18	0,47	8,94
191029_S49	137,65	0,58	9,09
191030_S50	135,74	0,54	10,22
191031_S51	149,65	0,59	10,51
191032_S52	136,21	0,61	10,16
191033_S53	141,75	0,53	10,13
191034_S54	134,40	0,48	9,04
191035_S55	135,45	0,53	9,77
191036_S56	135,18	0,52	8,76
191037_S57	134,05	0,51	10,43
191037_S58	135,21	0,55	9,77
191039_S59	135,62	0,55	9,67
191040_S60	126,50	0,51	9,73
191041_S61	130,33	0,50	10,06
191042_S62	140,22	0,54	10,86
191043_S63	137,01	0,54	11,25
191044_S64	132,22	0,54	10,10
Minimum	115,60	0,32	7,51
Median	133,83	0,51	9,06
Maximum	149,65	0,61	11,25
Mean	132,67	0,50	9,12
Standard deviation	6,93	0,06	0,93

Table of contents

1. RNA-Seq analysis 3
    1.1 Read count statistics 3
    1.2 Fragment counting statistics 6
    1.3 Spike-in quality control 7
    1.4 Strand specificity 7
    1.5 Adapter read-through 9
    1.6 Distribution of biotypes 10
    1.7 Transcript length coverage 11

1. RNA-Seq analysis

1.1 Read count statistics

For paired data, there are two reads in a pair.

The table is based on 64 samples.

Sample name	Read count	Single, mapped (%)	Single, not mapped (%)	Paired, mapped pairs (%)
190979_S1	45.730.142	-	-	92,76
190980_S2	51.523.428	-	-	93,03
190981_S3	68.280.202	-	-	92,65
190982_S4	67.370.742	-	-	92,68
190983_S5	75.992.602	-	-	92,93
190984_S6	75.654.926	-	-	93,24
190985_S7	75.708.206	-	-	93,44
190986_S8	45.261.328	-	-	92,97
190987_S9	66.705.176	-	-	92,39
190988_S10	95.032.006	-	-	93,25
190989_S11	70.793.952	-	-	94,02
190992_S12	79.084.928	-	-	93,91
190993_S13	62.347.668	-	-	93,97
190994_S14	66.006.692	-	-	93,78
190995_S15	70.987.898	-	-	94,71
190996_S16	63.979.056	-	-	94,24
190997_S17	83.622.406	-	-	94,17
190998_S18	109.481.358	-	-	93,52
190999_S19	72.779.522	-	-	92,73
191000_S20	61.462.432	-	-	92,08
191001_S21	79.376.768	-	-	94,03
191002_S22	94.262.194	-	-	93,98
191003_S23	98.416.680	-	-	93,50
191004_S24	84.799.582	-	-	93,56
191005_S25	82.520.478	-	-	93,18
191006_S26	102.294.586	-	-	93,84
191007_S27	95.424.968	-	-	93,70
191008_S28	69.226.706	-	-	93,00
191009_S29	94.784.344	-	-	93,76
191010_S30	88.907.246	-	-	93,79

Sample name	Read count	Single, mapped (%)	Single, not mapped (%)	Paired, mapped pairs (%)
191011_S31	81.777.778	-	-	94,10
191012_S32	80.358.054	-	-	93,42
191013_S33	87.694.364	-	-	93,70
191014_S34	80.949.620	-	-	93,49
191015_S35	86.212.554	-	-	93,34
191016_S36	92.507.066	-	-	93,44
191017_S37	93.716.980	-	-	93,54
191018_S38	75.131.274	-	-	93,88
191019_S39	92.463.912	-	-	93,28
191020_S40	97.395.280	-	-	93,21
191021_S41	86.095.072	-	-	93,09
191022_S42	106.329.220	-	-	93,15
191023_S43	68.946.230	-	-	93,41
191024_S44	85.130.792	-	-	93,38
191025_S45	119.038.014	-	-	93,73
191026_S46	101.643.912	-	-	93,60
191027_S47	108.047.688	-	-	93,68
191028_S48	75.115.960	-	-	93,37
191029_S49	97.703.226	-	-	93,47
191030_S50	96.023.250	-	-	93,84
191031_S51	103.122.202	-	-	92,67
191032_S52	85.261.764	-	-	93,15
191033_S53	96.183.854	-	-	93,55
191034_S54	87.813.490	-	-	93,98
191035_S55	106.316.144	-	-	94,40
191036_S56	79.623.064	-	-	93,64
191037_S57	104.083.706	-	-	94,10
191038_S58	110.954.444	-	-	94,03
191039_S59	121.109.956	-	-	94,17
191040_S60	110.785.244	-	-	94,01
191041_S61	101.804.654	-	-	94,49
191042_S62	99.810.434	-	-	93,77
191043_S63	103.368.146	-	-	93,85
191044_S64	99.065.658	-	-	93,79
Minimum	45.261.328,00	-	-	92,08
Median	86.153.813,00	-	-	93,56
Maximum	121.109.956,00	-	-	94,71
Mean	86.240.644,19	-	-	93,54

Sample name	Read count	Single, mapped (%)	Single, not mapped (%)	Paired, mapped pairs (%)
Standard deviation	16.947.346,09	-	-	0,51

Sample name	Paired, broken pairs (%)	Paired, not mapped (%)
190979_S1	1,09	6,15
190980_S2	1,31	5,65
190981_S3	1,43	5,91
190982_S4	1,52	5,80
190983_S5	1,34	5,73
190984_S6	1,35	5,41
190985_S7	1,36	5,20
190986_S8	1,01	6,03
190987_S9	2,29	5,32
190988_S10	2,53	4,21
190989_S11	2,13	3,86
190992_S12	1,90	4,19
190993_S13	1,84	4,19
190994_S14	1,97	4,26
190995_S15	1,39	3,90
190996_S16	1,68	4,09
190997_S17	1,54	4,29
190998_S18	2,05	4,43
190999_S19	2,34	4,93
191000_S20	2,39	5,53
191001_S21	1,90	4,07
191002_S22	1,86	4,16
191003_S23	2,18	4,32
191004_S24	1,77	4,67
191005_S25	1,97	4,85
191006_S26	1,61	4,55
191007_S27	1,63	4,67
191008_S28	1,81	5,19
191009_S29	1,84	4,40
191010_S30	1,85	4,35
191011_S31	1,55	4,35
191012_S32	1,70	4,88
191013_S33	1,22	5,08
191014_S34	1,24	5,27
191015_S35	1,51	5,15
191016_S36	1,26	5,30

Sample name	Paired, broken pairs (%)	Paired, not mapped (%)
191017_S37	1,58	4,88
191018_S38	1,55	4,58
191019_S39	1,76	4,96
191020_S40	1,66	5,13
191021_S41	1,37	5,55
191022_S42	1,32	5,53
191023_S43	1,46	5,13
191024_S44	1,40	5,22
191025_S45	1,63	4,64
191026_S46	1,52	4,88
191027_S47	1,31	5,01
191028_S48	1,40	5,23
191029_S49	1,31	5,22
191030_S50	1,33	4,83
191031_S51	0,76	6,57
191032_S52	1,45	5,40
191033_S53	1,13	5,31
191034_S54	1,25	4,76
191035_S55	1,21	4,39
191036_S56	1,44	4,92
191037_S57	1,42	4,49
191038_S58	1,36	4,61
191039_S59	1,31	4,52
191040_S60	1,81	4,18
191041_S61	1,46	4,05
191042_S62	1,18	5,06
191043_S63	1,42	4,73
191044_S64	1,59	4,62
Minimum	0,76	3,86
Median	1,52	4,88
Maximum	2,53	6,57
Mean	1,57	4,89
Standard deviation	0,35	0,59

1.2 Fragment counting statistics

An intact pair is counted as one, broken pairs are ignored.

The box plot is based on 64 samples.

1.3 Spike-in quality control

No data available

1.4 Strand specificity

The table is based on 64 samples.

Sample name	Strand specific setting	Forward reads mapped (%)	Reverse reads mapped (%)	Ignored reads (wrong strand) (%)
190979_S1	Forward	100	0	1,91
190980_S2	Forward	100	0	1,81
190981_S3	Forward	100	0	1,88
190982_S4	Forward	100	0	1,95
190983_S5	Forward	100	0	1,93
190984_S6	Forward	100	0	1,66
190985_S7	Forward	100	0	1,77
190986_S8	Forward	100	0	1,67
190987_S9	Forward	100	0	1,95
190988_S10	Forward	100	0	1,70
190989_S11	Forward	100	0	1,37

Sample name	Strand specific setting	Forward reads mapped (%)	Reverse reads mapped (%)	Ignored reads (wrong strand) (%)
190992_S12	Forward	100	0	1,60
190993_S13	Forward	100	0	1,61
190994_S14	Forward	100	0	1,45
190995_S15	Forward	100	0	1,28
190996_S16	Forward	100	0	1,35
190997_S17	Forward	100	0	1,46
190998_S18	Forward	100	0	1,34
190999_S19	Forward	100	0	1,53
191000_S20	Forward	100	0	1,78
191001_S21	Forward	100	0	1,45
191002_S22	Forward	100	0	1,55
191003_S23	Forward	100	0	1,52
191004_S24	Forward	100	0	1,65
191005_S25	Forward	100	0	1,54
191006_S26	Forward	100	0	1,53
191007_S27	Forward	100	0	1,63
191008_S28	Forward	100	0	1,89
191009_S29	Forward	100	0	1,89
191010_S30	Forward	100	0	1,78
191011_S31	Forward	100	0	1,75
191012_S32	Forward	100	0	1,79
191013_S33	Forward	100	0	1,31
191014_S34	Forward	100	0	1,43
191015_S35	Forward	100	0	1,42
191016_S36	Forward	100	0	1,47
191017_S37	Forward	100	0	1,76
191018_S38	Forward	100	0	1,44
191019_S39	Forward	100	0	1,49
191020_S40	Forward	100	0	1,45
191021_S41	Forward	100	0	1,51
191022_S42	Forward	100	0	1,46
191023_S43	Forward	100	0	1,54
191024_S44	Forward	100	0	1,50
191025_S45	Forward	100	0	1,67
191026_S46	Forward	100	0	1,76
191027_S47	Forward	100	0	1,57
191028_S48	Forward	100	0	1,63
191029_S49	Forward	100	0	1,46
191030_S50	Forward	100	0	1,40

Sample name	Strand specific setting	Forward reads mapped (%)	Reverse reads mapped (%)	Ignored reads (wrong strand) (%)
191031_S51	Forward	100	0	1,64
191032_S52	Forward	100	0	1,57
191033_S53	Forward	100	0	1,48
191034_S54	Forward	100	0	1,68
191035_S55	Forward	100	0	1,29
191036_S56	Forward	100	0	1,62
191037_S57	Forward	100	0	1,33
191038_S58	Forward	100	0	1,68
191039_S59	Forward	100	0	1,59
191040_S60	Forward	100	0	1,64
191041_S61	Forward	100	0	1,31
191042_S62	Forward	100	0	1,55
191043_S63	Forward	100	0	1,23
191044_S64	Forward	100	0	1,57
Minimum	-	100,00	0,00	1,23
Median	-	100,00	0,00	1,57
Maximum	-	100,00	0,00	1,95
Mean	-	100,00	0,00	1,58
Standard deviation	-	0,00	0,00	0,18

1.5 Adapter read-through

The box plot is based on 64 samples.

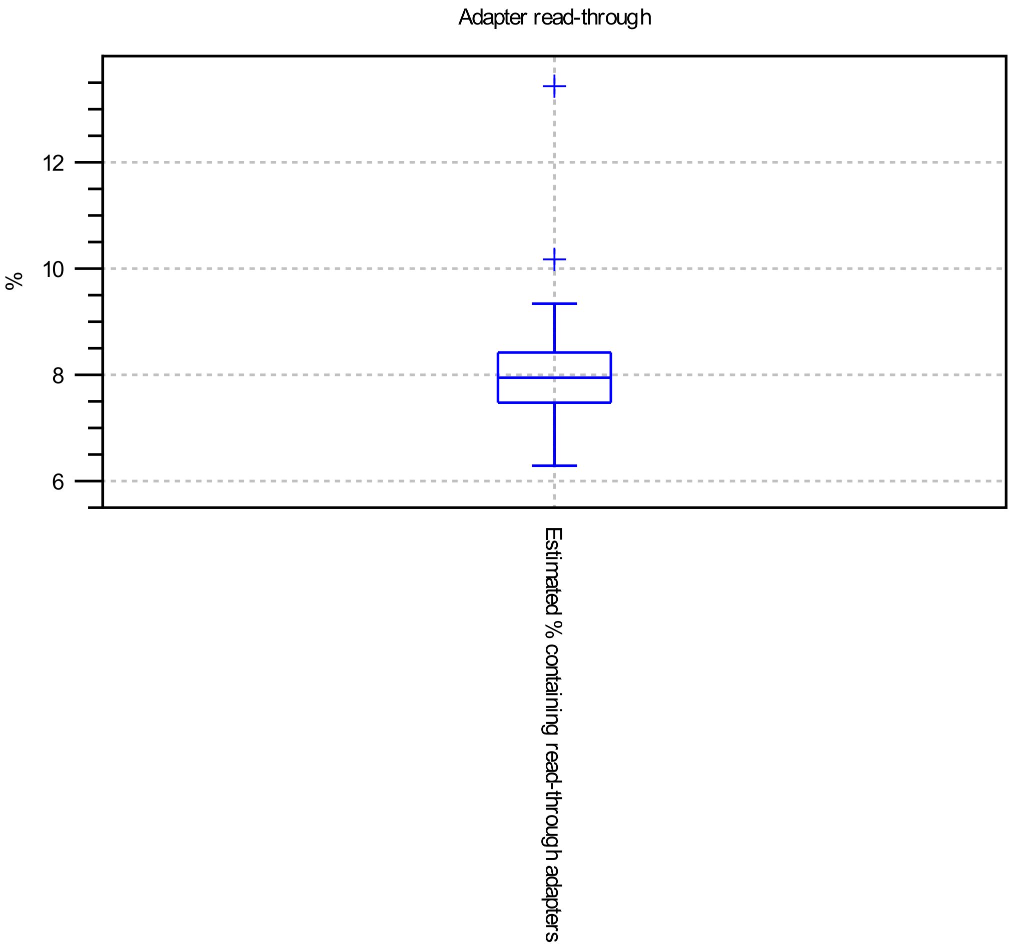

Estimated % containing read-through adapters: >10% of paired-end reads are estimated to contain read-through adapters.

- If the starts of the reads have been trimmed (5' trim), consider ignoring this warning.
- Consider removing the adapters.
- In future experiments, consider selecting fragments that are longer than the read size.

1.6 Distribution of biotypes

The box plot is based on 64 samples.

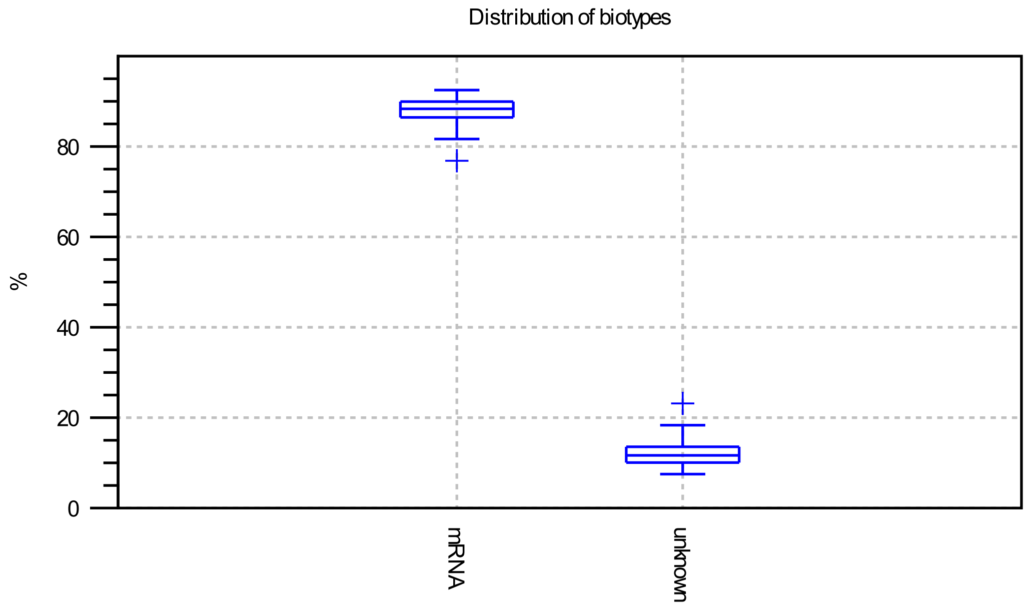

1.7 Transcript length coverage

The table is based on 64 samples.

Sample name	Expected coverage bias	Difference between average 3' and 5' normalized counts	Reads mapping to transcripts that are longer than 10,000 bp (%)
190979_S1	Unbiased	1,17	3,68
190980_S2	Unbiased	1,81	3,42
190981_S3	Unbiased	5,86	3,56
190982_S4	Unbiased	1,82	3,80
190983_S5	Unbiased	-1,12	3,57
190984_S6	Unbiased	-0,12	3,78
190985_S7	Unbiased	11,39	3,44
190986_S8	Unbiased	5,12	4,13
190987_S9	Unbiased	-2,67	3,52
190988_S10	Unbiased	-3,92	3,28
190989_S11	Unbiased	-2,27	3,44
190992_S12	Unbiased	0,27	3,51
190993_S13	Unbiased	-8,68	3,76
190994_S14	Unbiased	-0,53	3,59
190995_S15	Unbiased	-4,88	2,82
190996_S16	Unbiased	-2,35	3,93
190997_S17	Unbiased	-2,85	4,49
190998_S18	Unbiased	-3,32	4,11

Sample name	Expected coverage bias	Difference between average 3’ and 5’ normalized counts	Reads mapping to transcripts that are longer than 10,000 bp (%)
190999_S19	Unbiased	-1,89	3,77
191000_S20	Unbiased	-1,50	3,54
191001_S21	Unbiased	-6,50	3,32
191002_S22	Unbiased	-5,00	3,61
191003_S23	Unbiased	-5,39	3,37
191004_S24	Unbiased	-4,75	3,76
191005_S25	Unbiased	3,18	4,47
191006_S26	Unbiased	-1,23	3,98
191007_S27	Unbiased	-4,16	3,48
191008_S28	Unbiased	-1,34	3,94
191009_S29	Unbiased	-3,41	3,68
191010_S30	Unbiased	-6,34	3,54
191011_S31	Unbiased	0,45	3,26
191012_S32	Unbiased	-2,07	3,52
191013_S33	Unbiased	0,85	4,06
191014_S34	Unbiased	-2,20	3,66
191015_S35	Unbiased	-4,89	3,44
191016_S36	Unbiased	1,39	4,10
191017_S37	Unbiased	5,57	3,91
191018_S38	Unbiased	0,40	3,22
191019_S39	Unbiased	2,87	3,63
191020_S40	Unbiased	2,09	3,33
191021_S41	Unbiased	-2,28	2,70
191022_S42	Unbiased	-5,61	3,07
191023_S43	Unbiased	-3,11	2,94
191024_S44	Unbiased	-4,00	2,99
191025_S45	Unbiased	2,34	3,04
191026_S46	Unbiased	1,06	3,47
191027_S47	Unbiased	2,54	3,87
191028_S48	Unbiased	2,55	4,44
191029_S49	Unbiased	6,34	3,79
191030_S50	Unbiased	-2,79	3,77
191031_S51	Unbiased	-1,76	4,11
191032_S52	Unbiased	-3,09	3,63
191033_S53	Unbiased	3,75	3,86
191034_S54	Unbiased	5,85	4,01
191035_S55	Unbiased	9,17	3,28
191036_S56	Unbiased	6,80	3,78

Sample name	Expected coverage bias	Difference between average 3’ and 5’ normalized counts	Reads mapping to transcripts that are longer than 10,000 bp (%)
191037_S57	Unbiased	0,52	3,17
191038_S58	Unbiased	0,28	3,36
191039_S59	Unbiased	2,65	3,19
191040_S60	Unbiased	-0,88	3,35
191041_S61	Unbiased	1,05	3,37
191042_S62	Unbiased	2,68	3,15
191043_S63	Unbiased	0,42	2,81
191044_S64	Unbiased	0,03	3,35
Minimum	-	-8,68	2,70
Median	-	-0,33	3,55
Maximum	-	11,39	4,49
Mean	-	-0,23	3,58
Standard deviation	-	3,93	0,39
